# Immunotherapy targeting isoDGR‐protein damage extends lifespan in a mouse model of protein deamidation

**DOI:** 10.15252/emmm.202318526

**Published:** 2023-11-16

**Authors:** Pazhanichamy Kalailingam, Khalilatul‐Hanisah Mohd‐Kahliab, SoFong Cam Ngan, Ranjith Iyappan, Evelin Melekh, Tian Lu, Gan Wei Zien, Bhargy Sharma, Tiannan Guo, Adam J MacNeil, Rebecca EK MacPherson, Evangelia Litsa Tsiani, Deborah D O'Leary, Kah Leong Lim, I Hsin Su, Yong‐Gui Gao, A Mark Richards, Raj N Kalaria, Christopher P Chen, Neil E McCarthy, Siu Kwan Sze

**Affiliations:** ^1^ School of Biological Sciences Nanyang Technological University Singapore Singapore; ^2^ Department of Health Sciences, Faculty of Applied Health Sciences Brock University St. Catharines ON Canada; ^3^ iMarker Lab, Westlake Laboratory of Life Sciences and Biomedicine, Key Laboratory of Structural Biology of Zhejiang Province, School of Life Sciences Westlake University Hangzhou China; ^4^ Lee Kong Chian School of Medicine Nanyang Technological University Singapore Singapore; ^5^ Department of Cardiology National University Heart Centre Singapore Singapore; ^6^ Department of Cardiology University of Otago Christchurch New Zealand; ^7^ Institute of Neuroscience, Campus for Ageing and Vitality Newcastle University Newcastle upon Tyne UK; ^8^ Memory, Aging and Cognition Centre National University Health System Singapore Singapore; ^9^ Centre for Immunobiology, The Blizard Institute, Bart's and The London School of Medicine and Dentistry Queen Mary University of London London UK

**Keywords:** immunotherapy, inflammation, isoDGR, lifespan, Pcmt1, Immunology

## Abstract

Aging results from the accumulation of molecular damage that impairs normal biochemical processes. We previously reported that age‐linked damage to amino acid sequence NGR (Asn‐Gly‐Arg) results in “gain‐of‐function” conformational switching to isoDGR (isoAsp‐Gly‐Arg). This integrin‐binding motif activates leukocytes and promotes chronic inflammation, which are characteristic features of age‐linked cardiovascular disorders. We now report that anti‐isoDGR immunotherapy mitigates lifespan reduction of Pcmt1^−/−^ mouse. We observed extensive accumulation of isoDGR and inflammatory cytokine expression in multiple tissues from Pcmt1^−/−^ and naturally aged WT animals, which could also be induced via injection of isoDGR‐modified plasma proteins or synthetic peptides into young WT animals. However, weekly injection of anti‐isoDGR mAb (1 mg/kg) was sufficient to significantly reduce isoDGR‐protein levels in body tissues, decreased pro‐inflammatory cytokine concentrations in blood plasma, improved cognition/coordination metrics, and extended the average lifespan of Pcmt1^−/−^ mice. Mechanistically, isoDGR‐mAb mediated immune clearance of damaged isoDGR‐proteins via antibody‐dependent cellular phagocytosis (ADCP). These results indicate that immunotherapy targeting age‐linked protein damage may represent an effective intervention strategy in a range of human degenerative disorders.

## Graphical abstract

Anti‐isoDGR immunotherapy enhances the healthspan of naturally aged mice and extends the lifespan of Pcmt1 KO mice by reducing chronic inflammation. The health benefit is mediated by immune clearance of damaged proteins by antibody‐dependent cellular phagocytosis (ADCP).

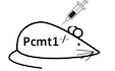

**The paper explained**

**Problem**

As people get older, their bodies accumulate harmful changes called isoDGR‐motif at a molecular level that can disrupt the normal functioning of their cells. These changes can trigger chronic inflammation in the body. This kind of inflammation is often associated with age‐related health problems. Clearing out these damaged proteins from the body may help people live longer and healthier.

**Results**

We conducted a study on mice that showed that a treatment that targets this specific isoDGR‐protein can reduce chronic inflammation and extend the lifespan/healthspan of the mice. We found that the premature aging mice and older normal mice had high levels of this protein in their tissues, which was associated with inflammation and other health problems. We also found that injecting synthetic isoDGR peptides into young mice could cause these same problems. However, when we treated the mice with a monoclonal antibody that targets and removes isoDGR from the body, it significantly reduced the amount of this protein and decreased inflammation. This treatment also improved the mice's behavior and coordination and significantly extended their average lifespan. We showed that this treatment works by helping the body's immune system clear out the damaged isoDGR proteins in tissues.

**Impact**

These findings suggest that using the immune system to target and remove proteins that are damaged during aging may be a successful way to treat age‐related health problems.

## Introduction

Aging is a complex process of time‐dependent decline in key biological functions, resulting in increased susceptibility to chronic diseases and reduced lifespan. At a molecular level, aging is thought to be underpinned by progressive biomolecular damage caused by degenerative protein modifications (DPMs), including oxidation, deamidation, glycation, and a range of other non‐enzymatic structural changes (Gallart‐Palau *et al*, 2015, 2019; Truscott *et al*, 2016; Delanghe *et al*, 2017; Hipp *et al*, 2019; Dai *et al*, 2020; Maksimovic & David, 2021; López‐Otín *et al*, 2023). We now recognize that aging is a consequence of deleterious chemical processes that damage biomolecules and impair the homeostatic functions programmed by our genomes (McKerrow & Robinson, 1974; Robinson & Robinson, 2001b; Clarke, 2003; da Costa *et al*, 2016; Gladyshev *et al*, 2021). However, it is unclear whether therapeutic targeting of these damaged biomolecules represents an effective strategy for maintaining tissue function and extending healthy lifespan.

The functional impact of DPMs depends on the mode of modification and the target molecule involved. For example, deamidation leads to the accumulation of isoaspartate residues that progressively disrupt protein integrity and alter biological activity (Geiger & Clarke, 1987; Kim *et al*, 1997, 2018; Yamamoto *et al*, 1998; Robinson & Robinson, 2001b; Adav *et al*, 2014; Qin *et al*, 2015; Adav & Sze, 2016; Hao *et al*, 2017; Juang *et al*, 2017; Park *et al*, 2021). However, “gain of function” structural changes caused by DPMs may play equally important roles in human pathology (Curnis *et al*, 2006; Spitaleri *et al*, 2008; Corti & Curnis, 2011; Cheow *et al*, 2013; Dutta *et al*, 2017; Park *et al*, 2021). Accumulation of isoaspartate residues can occur via deamidation of asparagine or isomerization of aspartic acid residues under the influence of microenvironmental stresses, flanking amino acid sequences, and genetic factors (Geiger & Clarke, 1987; Robinson & Robinson, 2001a, 2001b; Hao *et al*, 2011, 2017; Juang *et al*, 2017; Sze *et al*, 2020). Consequently, DPMs greatly increase the diversity of biomolecules present in body tissues (Smith *et al*, 2019), with a high probability of generating proteoforms capable of interacting with or binding to key biomolecules in novel ways. Indeed, we recently reported that deamidation of the amino acid sequence NGR (Asn‐Gly‐Arg) in extracellular matrix (ECM) proteins results in “gain‐of‐function” conformational switching to isoDGR (isoAsp‐Gly‐Arg) motifs (Cheow *et al*, 2013; Dutta *et al*, 2017; Park *et al*, 2021) that can bind to integrins and promote immune cell activation (Curnis *et al*, 2006; Spitaleri *et al*, 2008; Corti & Curnis, 2011; Dutta *et al*, 2017). Unlike isoaspartate‐modified proteins within cells that can be repaired by the Pcmt1 enzyme, long‐lived ECM proteins cannot be repaired by intracellular mechanisms and are thus susceptible to progressive damage over time (Geiger & Clarke, 1987; Robinson & Robinson, 2001b; Gallart‐Palau *et al*, 2015; Truscott *et al*, 2016). Accordingly, age‐linked isoDGR modifications have previously been detected in fibronectin, laminin, tenascin C, and several other ECM proteins derived from human carotid plaque tissues (Cheow *et al*, 2013; Hao *et al*, 2014; Dutta *et al*, 2017), suggesting that these molecules may be capable of enhancing leukocyte binding to the atherosclerotic matrix (Dutta *et al*, 2017; Park *et al*, 2021). While age‐associated DPMs have long been implicated in a range of chronic diseases (McKerrow & Robinson, 1974; Clarke, 2003; Gallart‐Palau *et al*, 2015, 2019; da Costa *et al*, 2016; Truscott *et al*, 2016; Delanghe *et al*, 2017; Hipp *et al*, 2019; Dai *et al*, 2020; Gladyshev *et al*, 2021; Maksimovic & David, 2021), and isoD accumulation has been identified as a “molecular clock” of aging (Robinson & Robinson, 2001b), the potential benefits of targeting these structures with specific immunotherapies remain largely unknown.

The protein l‐isoaspartate (d‐aspartate)‐*O*‐methyltransferase (Pcmt1) enzyme is expressed in all mammalian tissues and mediates the repair of age‐linked protein damage (by promoting conversion of abnormal aspartyl residues to l‐aspartyl forms) (Geiger & Clarke, 1987; Kim *et al*, 1997). Previous studies have shown that global deletion of Pcmt1 in mice leads to the accumulation of isoaspartate in all body tissues, followed by death at around 42 days (Kim *et al*, 1997, 1999; Yamamoto *et al*, 1998), modeling natural decline in protein repair function as animals age. Moreover, genetic association studies in humans have also identified a relationship between PCMT1 function and various age‐linked disorders (DeVry & Clarke, 1999; Kosugi *et al*, 2008; Desrosiers & Fanélus, 2011; D'Angelo *et al*, 2013; Adav *et al*, 2014; Biterge *et al*, 2014; Ogasawara *et al*, 2016; Juang *et al*, 2017; Kim *et al*, 2018; Warmack *et al*, 2019; Chatterjee *et al*, 2020; Lv *et al*, 2021). Having previously observed that long‐lived matrix proteins undergo age‐dependent deamidation (Cheow *et al*, 2013; Dutta *et al*, 2017), we hypothesized that isoDGR modification of ECM may represent the mechanistic link in common pathologies affecting both elderly humans and Pcmt1^−/−^ mice. We previously observed that isoDGR motifs accumulate in blood plasma and body tissues, which activates CD68^+^ macrophage to express pro‐inflammatory cytokines (Park *et al*, 2021). We also confirmed that interaction of isoDGR‐modified proteins with macrophage integrins underpinned this pathology, suggesting a potential role for this axis in the “inflammaging” characteristics of advanced age (Ferrucci & Fabbri, 2018; Franceschi *et al*, 2018; Liberale *et al*, 2020). Moreover, a (L‐isoD)GR‐specific monoclonal antibody has been successfully generated and characterized (Park *et al*, 2021). In the current study, we therefore tested whether anti‐isoDGR immunotherapy can reduce chronic inflammation and extend the healthspan of Pcmt1^−/−^ and naturally aged WT mice.

The human immune system has evolved to recognize and eliminate pathogens as well as clearing dead cell debris through both innate and adaptive responses (Mulder *et al*, 2019). Monoclonal antibody technology now allows these natural defense mechanisms to be harnessed for specific immunotherapy in cancer, autoimmunity, infectious diseases, and many other disorders (Oostindie *et al*, 2022). In addition to neutralizing harmful agents, monoclonal antibodies can interact with leukocyte FcγRs to trigger effector functions including antibody‐dependent cellular toxicity (ADCC) and antibody‐dependent cellular phagocytosis (ADCP) (Nimmerjahn *et al*, 2015; Maskalenko *et al*, 2022). ADCC is typically mediated by natural killer cells, which efficiently target and eliminate tumors. ADCP is instead carried out by macrophages or other phagocytes which engulf unwanted cells and debris, including abnormal host proteins. Monoclonal antibodies may therefore be able to induce clearance of specific protein damage from body tissues *in vivo*, thereby alleviating age‐linked pathology and potentially extending healthy lifespan.

Here we report that weekly injection of 1 mg/kg isoDGR‐specific monoclonal antibody (mAb) significantly increased body weight, improved behavior and coordination, and doubled average lifespan in a Pcmt1^−/−^ mouse model of chronic inflammation. In addition, mAb treatment decreased levels of circulating pro‐inflammatory cytokines and reduced tissue inflammation in Pcmt1^−/−^ mice, strongly suggesting that isoDGR‐modified proteins are at least partly responsible for the pathology observed in these animals. In parallel experiments using naturally aged WT mice, target‐specific mAb injection also significantly reduced circulating pro‐inflammatory cytokines and tissue inflammation in treated animals. Subsequent functional assays demonstrated that anti‐isoDGR mAb can induce immune clearance of the target motif via ADCP both *in vitro* and *in vivo*, while Western blot analysis revealed a significant reduction in isoDGR‐modified protein levels in both brain and liver tissues from isoDGR‐specific mAb treated but not isotype mAb‐injected mice. While damaged proteins are known to accumulate in body tissues with advancing age, our data suggest that immunotherapy targeting these damaged proteins may be an effective intervention in age‐linked disorders and could potentially extend healthy human lifespan.

## Results

### Anti‐isoDGR immunotherapy doubles the lifespan of Pcmt1^−/−^ mice

**Figure 1:**
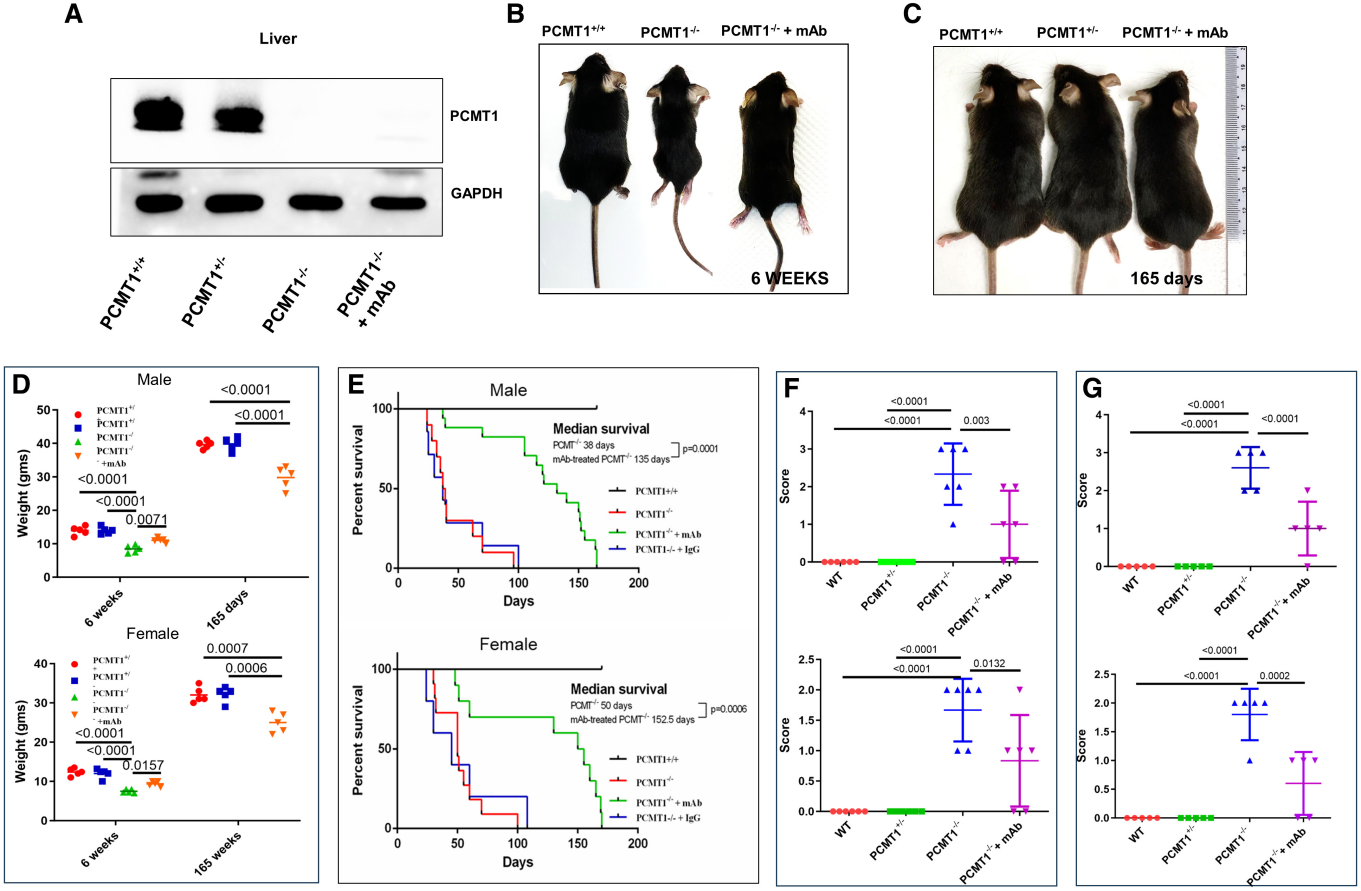
IsoDGR mAb treatment improves motor function and extends lifespan of Pcmt1^−/−^ mice AProtein lysate from liver of Pcmt1^+/+^ (left), Pcmt1^+/−^, Pcmt1^−/−^, and mAb‐treated Pcmt1^−/−^ mice (right) were subjected to Western blot using antibodies against Pcmt1 (GAPDH was used as a loading control).BRepresentative images of Pcmt1^+/+^ (left), Pcmt1^−/−^, and mAb‐treated Pcmt1^−/−^ mice (right) at 6 weeks.CPcmt1^+/+^ (left), Pcmt1^+/−^, and mAb‐treated Pcmt1^−/−^ mice (right) at 165 days of age.DDot plot shows average body weight (male or female) for each genotype at 6 weeks and 165 days of age (*n* = 5).EGraph shows percent survival after birth for Pcmt1^+/+^, Pcmt1^−/−^ and mAb‐treated Pcmt1^−/−^ mice (male or female).F, G(F) Dot plot shows quantitative analysis of hind‐limb clasping test (*n* = 6) and (G) ledge scores (*n* = 5); Pcmt1^−/−^ mice achieved higher scores than Pcmt1^+/+^ mice, while anti‐isoDGR mAb treatment reduced scores in Pcmt1^−/−^ animals at 6 weeks. Data information: In this figure, we used 5 mice per group for experiments showed in (A–D), 5–17 mice showed in (E), and 6 mice showed in (F and G). Statistical significances were determined by 1‐way ANOVA, and survival were evaluated using Mantel‐Cox log‐rank test. Results are shown as mean ± SD. Source data are available online for this figure.

Global deletion of repair enzyme Pcmt1 leads to tissue accumulation of isoaspartate residues and premature death of Pcmt1^−/−^ mice (Kim *et al*, 1997; Yamamoto *et al*, 1998), but the mechanistic basis of this pathology is not fully understood. We have previously shown that isoDGR‐modified fibronectin is a critical mediator of vascular inflammation in Pcmt1^+/−^ mice via interaction with monocyte–macrophages in an atherosclerotic CVD model (Park *et al*, 2021). We observed that isoDGR‐modified proteins positively correlated with CD68^+^ macrophage cell frequency in both intima and adventitial layers of the aorta, with isoDGR engagement of macrophage integrins playing a key role in triggering inflammatory cytokine release and progression of atherosclerotic lesions. To fully elucidate the role of isoDGR motifs in age‐linked disease, we generated global Pcmt1^−/−^ mice by crossing Pcmt1^+/−^ parents (25% of pups born were Pcmt1^−/−^ consistent with the expected Mendelian ratio). Genotypes were confirmed by PCR of tail genomic DNA and Western blot analysis of liver protein extracts (Fig 1A). Pcmt1^−/−^ neonates were viable but displayed significantly reduced body weights, chronic inflammation, and died prematurely compared to Pcmt^+/+^ mice. We hypothesized that isoDGR‐induced tissue inflammation is partly responsible for the pathology observed in Pcmt1^−/−^ mice. We therefore tested if anti‐isoDGR immunotherapy using a motif‐specific mAb could be used to reduce levels of damaged proteins in body tissues and decrease systemic inflammation in Pcmt1^−/−^ pups. Strikingly, intraperitoneal injection (i.p.) of 1 mg/kg/week anti‐isoDGR mAb significantly increased body weight and reduced inflammation in Pcmt1^−/−^ mice (Fig 1B–D), while also doubling the average lifespan of both male and female Pcmt1^−/−^ mice relative to untreated or IgG1 isotype control‐treated mice (Fig 1E). These data strongly suggested that isoDGR‐modified proteins can be targeted with specific immunotherapy to reduce tissue damage, decrease inflammation, and extend lifespan *in vivo*.

### IsoDGR‐specific antibody therapy improves motor function of Pcmt1^−/−^ mice

Deletion of Pcmt1 in mice causes severe motor dysfunction associated with seizure activity. Cytosolic content from Pcmt1‐deficient tissues contained significantly elevated levels of protein damage compared to Pcmt1^+/+^ tissues (as evidenced by 4‐ to 8‐fold higher amounts of isoaspartyl residues). Intriguingly, protein damage accumulated to particularly high levels in brain cytosol fractions from Pcmt1^−/−^ mice (Kim *et al*, 1997; Lowenson *et al*, 2001). We therefore proceeded to test the ability of isoDGR‐specific immunotherapy to preserve motor and cognitive functions in Pcmt1^−/−^ mice by using clasping and ledge behavior tests (Cahill *et al*, 2019; Schoonover *et al*, 2020; Castillo‐Mariqueo & Giménez‐Llort, 2022). Six‐week‐old Pcmt1^+/+^ and Pcmt1^+/−^ mice displayed normal extension reflex in the hind‐limbs and used body torsion when suspended in the air. In contrast, Pcmt1^−/−^ mice exhibited severe hind‐limb clasping and displayed high dysfunction scores that improved upon treatment with isoDGR‐specific mAb (Fig 1F). Ledge tests also revealed impaired motor coordination in Pcmt1^−/−^ mice, as evidenced by difficulty in paw placement and multiple limb slips during forward movement. Consequently, KO mice took longer to traverse the ledge than did Pcmt1^+/+^ and Pcmt1^+/−^ mice. However, administration of isoDGR‐specific mAb was sufficient to improve paw placement and reduce limb slip frequency during forward movement of Pcmt1^−/−^ mice (Fig 1G).

### Anti‐isoDGR mAb treatment reduces isoDGR‐modified protein levels in body tissues

**Figure 2:**
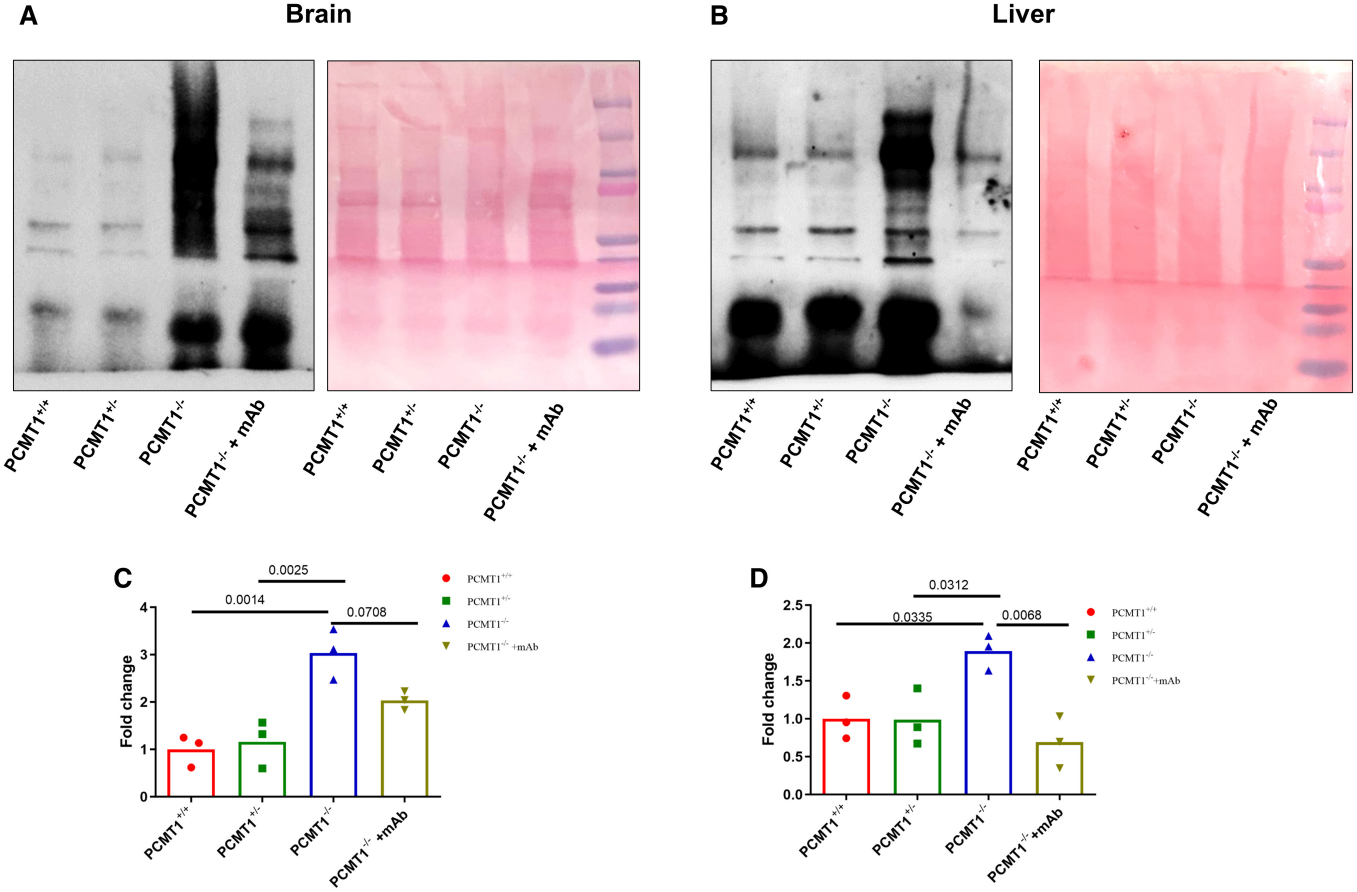
Anti‐isoDGR mAb treatment reduces motif level in brain and liver from Pcmt1^−/−^ mice A–DProtein lysates from brain (A) and liver (B) of 6‐week‐old Pcmt1^+/+^, Pcmt1^+/−^, Pcmt1^−/−^, and mAb‐treated Pcmt1^−/−^mice were subjected to Western blot using isoDGR‐specific antibody. Young Pcmt1^+/−^ mice have isoDGR levels comparable to Pcmt1^+/+^ mice because they possess one copy of the Pcmt1 allele, allowing them to express the functional repair enzyme. Protein loadings were visualized by Ponceau S. Graphs show quantification of isoDGR‐modified protein levels in mouse brain (C) and liver (D), individual points represent individual mice. Data information: In this figure, we used 3 mice per group for all experiments. We compared four groups of mice and assessed statistical significance through one‐way ANOVA. Source data are available online for this figure.

To understand the mechanism underlying these observed phenotype changes, we next proceeded to test whether the therapeutic effects of anti‐isoDGR antibody could be attributed to antigen neutralization or antibody‐dependent effector functions mediated via leukocyte Fc receptors (Biburger *et al*, 2014; Pinto *et al*, 2022). If anti‐isoDGR mAb simply neutralized the target motif *in vivo*, the overall abundance of isoDGR‐damaged proteins in tissues should remain unchanged. Alternatively, if the beneficial effects of treatment were mediated by antibody‐induced leukocyte clearance of isoDGR‐damaged proteins, motif levels should be reduced in body tissues. We therefore assessed isoDGR‐modified protein levels by Western blot analysis of whole brain and liver lysates from 6‐week‐old Pcmt1^−/−^ and naturally aged WT mice that had been treated or not with anti‐isoDGR mAb. This analysis indicated that Pcmt1^−/−^ and old WT mice accumulated large quantities of isoDGR‐damaged proteins in both liver and brain relative to young Pcmt1^+/+^ and Pcmt1^+/−^ mice (Fig 2A and B), whereas isoDGR levels were significantly reduced by specific mAb treatment (Fig 2C and D). While the blood–brain barrier can limit antibody penetration into brain tissue, we nonetheless observed a substantial reduction of isoDGR‐modified proteins in mice treated with target‐specific mAb (Fig 2C). These data resemble the distribution of FDA‐approved therapeutic mAbs used to promote immune clearance of beta‐amyloid deposits from the brain in Alzheimer's disease (Sevigny *et al*, 2017; Shcherbinin *et al*, 2022; van Dyck *et al*, 2023).

### IsoDGR mAb reduces macrophage infiltration and inflammation of Pcmt1^−/−^ liver

**Figure 3:**
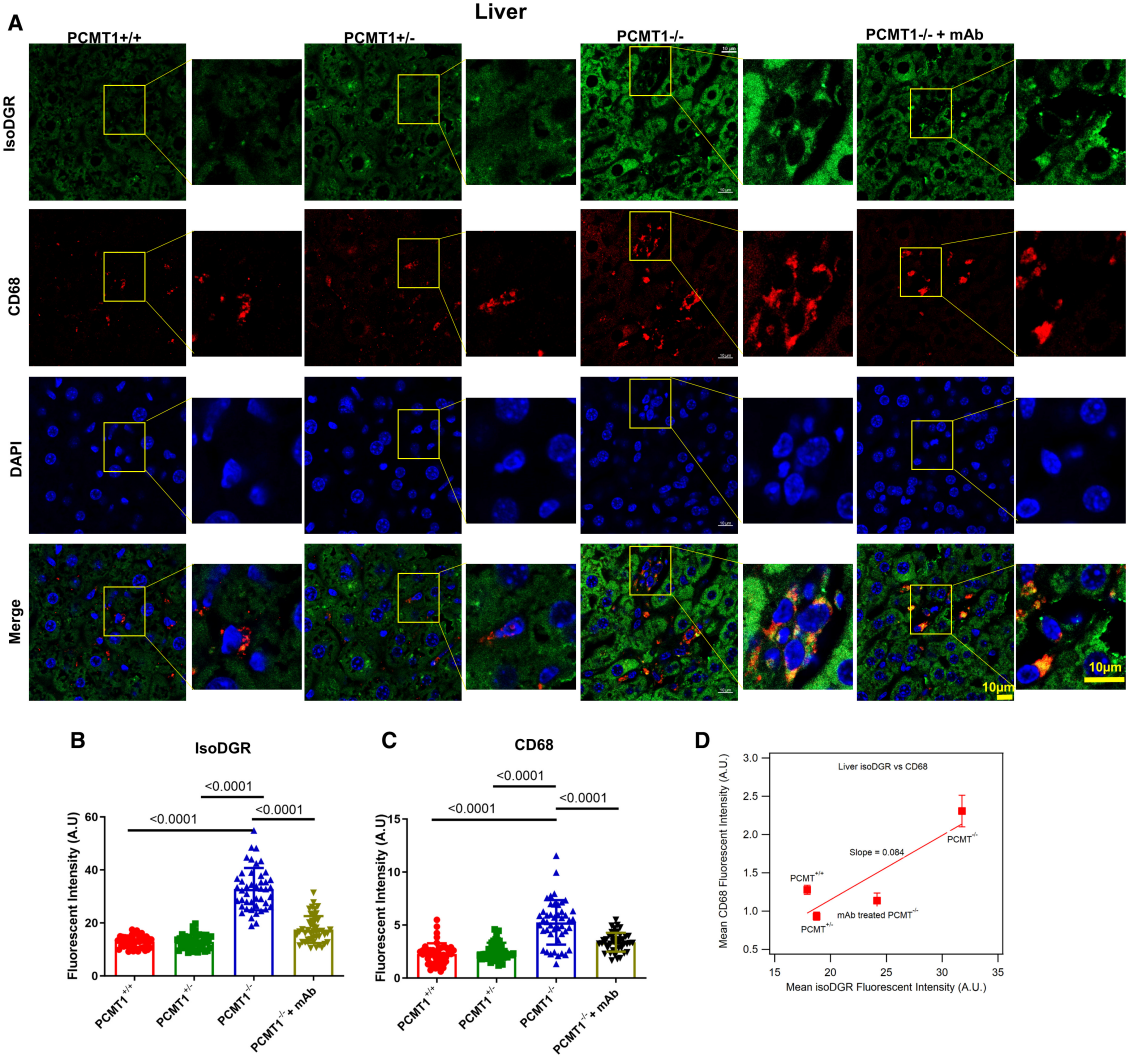
IsoDGR correlation with CD68^+^ monocyte–macrophages in liver of Pcmt1^−/−^ mice in response to anti‐isoDGR treatment ARepresentative immunostaining images showing isoDGR distribution and correlation with CD68^+^ macrophages in cryosectioned liver tissue (Pcmt1^+/+^, Pcmt1^+/−^ Pcmt1^−/−^, and mAb‐treated Pcmt1^−/−^ mice at 6 weeks; *n* = 6).B, C(B) IsoDGR or (C) CD68 fluorescence in 50 randomized regions from 3 images of 6 independent liver sections for each genotype were quantified using image J (graphs show averaged values for the same region from 6 images).DPlot showing the CD68 is proportionally increased with isoDGR accumulation, the slope indicates the sensitivity of the isoDGR‐induced CD68^+^ cells infiltration (average of 6 mice from B and C). Data information: In this figure, we utilized 6 mice per condition for all experiments. We randomly selected 50 regions from 6 images for statistical analysis. Statistical significance was assessed using the Kruskal–Wallis test, and the results are presented as mean values with standard errors (SE). Source data are available online for this figure.

We previously identified that patients with atherosclerotic CVD display marked isoDGR accumulation in ECM components of the vascular wall (Cheow *et al*, 2013; Dutta *et al*, 2017; Park *et al*, 2021), which was also observed in aortic tissues from 8‐month‐old Pcmt1^+/+^ and Pcmt1^+/−^ mice. Immunofluorescent imaging revealed that isoDGR motifs positively correlated with CD68^+^ macrophage infiltration of the vessel wall (Park *et al*, 2021), and this interaction was later confirmed to be mediated by integrin binding. Importantly, isoDGR‐macrophage interactions triggered the secretion of several pro‐inflammatory cytokines/chemokines including MCP1 and TNFα, thereby promoting vascular inflammation. Given our new finding that isoDGR‐damaged proteins also accumulate in the liver and brain of Pcmt1^−/−^ mice, we next used immunofluorescent imaging to detect isoDGR‐modified proteins and assessed CD68^+^ macrophage distribution across multiple organs. Consistent with the Western blot results, we observed that Pcmt1^−/−^ mice display substantial accumulation of isoDGR in the liver (Fig 3), spleen (Appendix Figs S1 and S2), and thymus tissue (Appendix Fig S3), that was significantly decreased by mAb treatment (Fig 3A and B). In line with our hypothesis, isoDGR positively correlated with marked infiltration of CD68^+^ monocytes‐macrophages into each of the tissues analyzed (Fig 3C). Indeed, frequency of CD68^+^ and F4/80^+^ monocyte–macrophages (Appendix Fig S2) increased proportionally with isoDGR levels across multiple tissues (Fig 3D, Appendix Figs S1D and S3D), whereas mAb treatment reduced F4/80^+^ monocyte–macrophage frequency in spleen from Pcmt1^−/−^ mice (6 weeks old) (Appendix Fig S2). Intriguingly, the lymphoid organs (spleen: slope = 0.55, Appendix Fig S1D; thymus: slope = 0.20, Appendix Fig S3D) are more sensitive to isoDGR‐induced CD68^+^ cells infiltration than the liver (slope = 0.086, Fig 3D), suggesting that aging damaged isoDGR‐proteins have a larger deleterious effect on these immune organs than the liver. Together, these results suggest that accumulation of isoDGR‐proteins may contribute to inflammaging in a Pcmt1^−/−^ mouse model, whereas treatment with 1 mg/kg/week anti‐isoDGR mAb was able to reduce isoDGR levels across all body tissues analyzed, leading to a concomitant decline in CD68^+^ macrophage infiltration (Fig 3A and C).

### IsoDGR accumulation induces both local and systemic inflammation in Pcmt1^−/−^ mice

**Figure 4:**
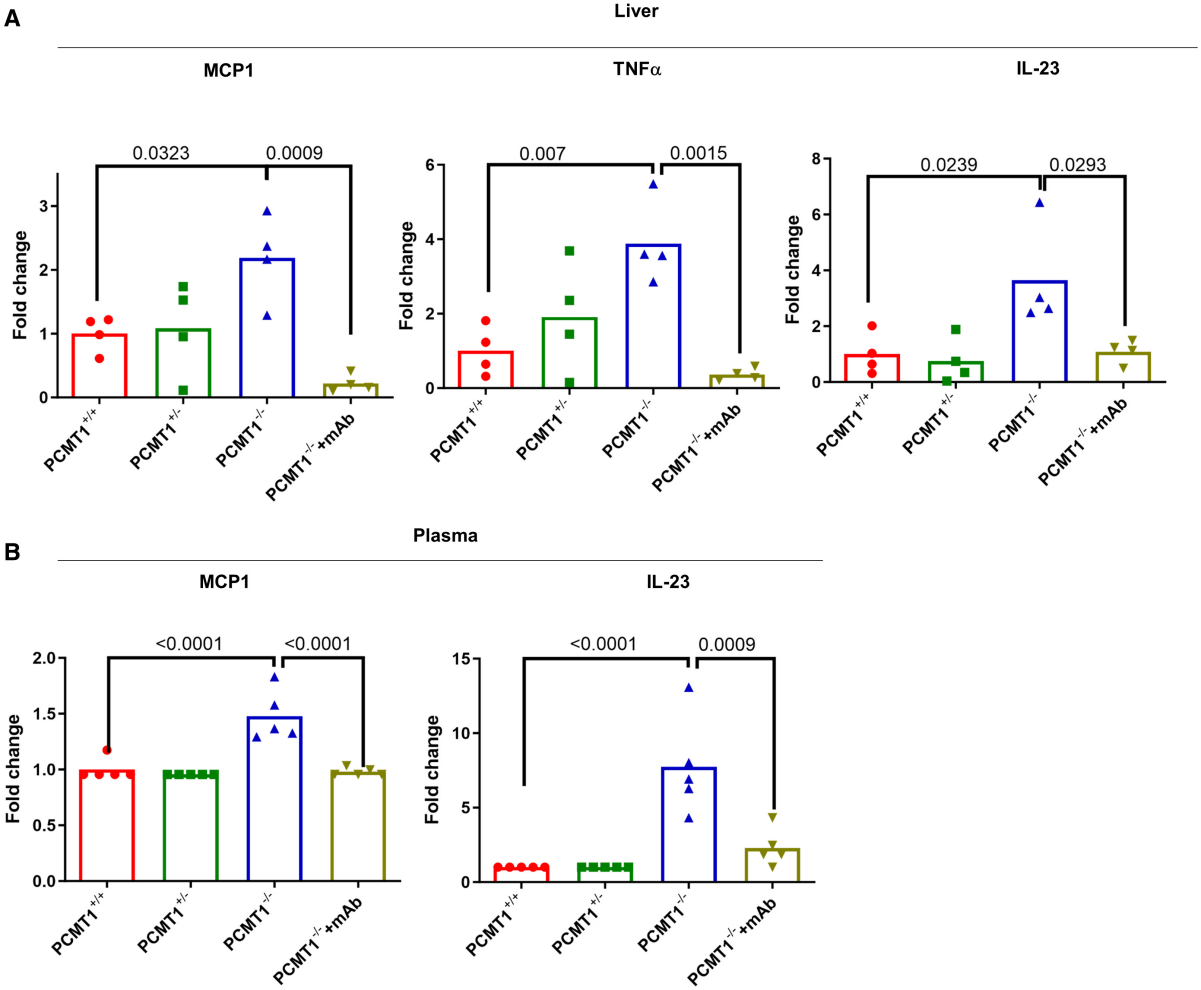
Elevated levels of local and systemic inflammation in Pcmt1^−/−^ mice Graph shows quantitative PCR analysis of pro‐inflammatory cytokines in liver tissue from Pcmt1^+/+^, Pcmt1^+/−^, Pcmt1^−/−^, and mAb‐treated Pcmt1^−/−^ mice at 6 weeks (*n* = 4).Graph shows cytokine quantification by multiplex bead array in blood plasma from Pcmt1^+/+^, Pcmt1^+/−^, Pcmt1^−/−^, and mAb‐treated Pcmt1^−/−^ mice at 6 weeks (*n* = 5). Data information: In this figure, we used 4–5 mice per group for all experiments. We compared four groups of mice and assessed statistical significance through one‐way ANOVA. The results are presented as mean values with standard errors (SE). Source data are available online for this figure.

IsoDGR‐damaged proteins are recognized by macrophage integrins and trigger pro‐inflammatory cytokine release and cytotoxic functions, suggesting a potential role in the inflammaging characteristic of older individuals. Our previous findings also indicated that positive correlation of isoDGR‐modified proteins with CD68^+^ macrophage frequency may be involved in chronic inflammation of the aorta in aged Pcmt1^+/−^ mice (Park *et al*, 2021). In earlier work, we observed that elevated levels of isoDGR‐modified fibronectin in Pcmt1^−/−^ mice can activate macrophages to secrete proinflammatory cytokines/chemokines that recruit blood monocytes into vascular tissues. To determine whether these effects also impact other tissues, in the current study we assessed expression levels of inflammatory cytokines via RT–qPCR analysis of total liver RNA from 6‐week‐old Pcmt1^+/−^, Pcmt1^−/−^, mAb‐treated Pcmt1^−/−^, and Pcmt1^+/+^ mice. These results revealed significantly higher expression of pro‐inflammatory factors including MCP1, TNFα, and IL23 in liver from Pcmt1^−/−^ mice, whereas mAb treatment effectively reduced expression of these mediators (Fig 4A). To test whether the pro‐inflammatory effects of isoDGR accumulation were local or systemic, we next assessed cytokine levels in blood plasma from Pcmt1^+/+^, Pcmt1^+/−^, Pcmt1^−/−^, and mAb‐treated Pcmt1^−/−^ mice, as well as naturally aged WT mice that were treated or not with isoDGR‐mAb. Multiplex bead arrays confirmed that elevated plasma cytokine concentrations in Pcmt1^−/−^ and naturally aged WT mice could be reversed by anti‐isoDGR immunotherapy. Individual mice with more extensive isoDGR accumulation (Figs 2 and 3, Appendix Figs S1–S3) also displayed corresponding higher levels of circulating pro‐inflammatory cytokines (Fig 4B).

### IsoDGR‐modified proteins induce inflammation in young WT mice

To determine whether isoDGR can directly induce inflammation *in vivo*, we next injected WT C57BL/6 mice with isoDGR‐modified plasma proteins (generated by incubating at pH 9 overnight to induce deamidation). After dialysis with 1× PBS, isoDGR‐modified plasma proteins and unmodified WT control plasma were injected intravenously into 6‐week‐old WT mice. After 24 h, we observed significantly higher levels of pro‐inflammatory cytokines in the circulation of mice treated with isoDGR‐plasma relative to WT plasma (Appendix Fig S4A). Further analysis by immunostaining revealed that mice injected with deamidated plasma proteins also displayed increased levels of isoDGR‐modified proteins in blood vessel walls that were positively correlated with CD68^+^ monocyte‐macrophage infiltration (Appendix Fig S4B). To further validate these findings, we next injected synthetic isoDGR peptide (Ac‐GC(isoD)GRCGK) or PBS control into WT mice and collected blood plasma for assessment of pro‐inflammatory cytokine levels 24 h later. Again, we observed a significant elevation of plasma MCP1, TNFα, IL‐1α, and IL‐6 concentrations in mice injected with isoDGR‐peptide (Appendix Fig S5). Together, these results suggest that isoDGR accumulation in blood plasma and body tissues can activate macrophages via integrin receptors to trigger the pathological release of pro‐inflammatory cytokines and chemokines.

### Accumulation of isoDGR modified proteins in liver during aging

**Figure 5:**
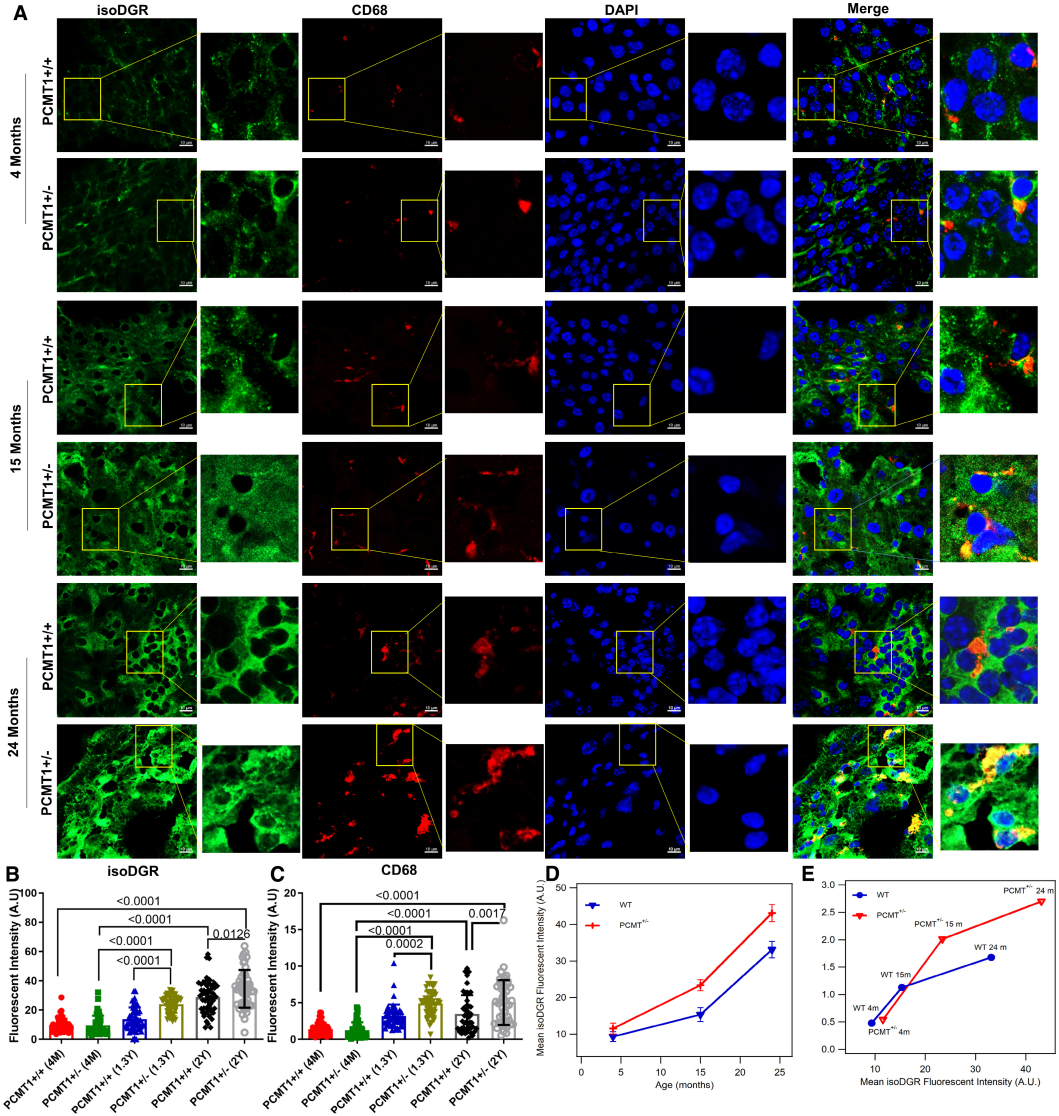
Age‐induced accumulation of isoDGR positively correlates with CD68^+^ monocyte–macrophage infiltration of liver from Pcmt1^+/+^ and Pcmt1^+/−^ mice ARepresentative immunostaining of isoDGR protein distribution and correlation with CD68^+^ macrophages in cryosectioned liver tissue from Pcmt1^+/+^ and Pcmt1^+/−^ mice at 4, 15 and 24 months.B, CIsoDGR (B) or CD68 (C) fluorescent intensities in a set of 50 random regions taken from 6 images representing 6 independent liver sections per genotype (quantified by Image J software with average values plotted in the dot/bar graph).DPlot showing the accumulation of isoDGR‐motif with age in both Pcmt1^+/+^ and Pcmt1^+/−^ animals. Results indicate that isoDGR accumulation is accelerated in older animal of both WT and HET mice (average of 6 mice from B).ECD68^+^ cells infiltration to tissues are proportional to the isoDGR levels (average of 6 mice from B and C). Data information: In this figure, we utilized 6 mice per condition for all experiments. We randomly selected 50 regions from 6 images for statistical analysis. Statistical significance was assessed using the Kruskal–Wallis test, and the results are presented as mean values with standard errors (SE). Source data are available online for this figure.

We next sought to confirm that isoDGR can naturally accumulate in body tissues with advancing age due to the declining activity of Pcmt1 enzyme. To do this, we used immunohistochemistry to interrogate isoDGR and CD68^+^ macrophage distribution in liver tissue from both Pcmt1^+/+^ and Pcmt1^+/−^ mice at 4, 15, and 24 months (Fig 5A–C) (Pcmt1^−/−^ mice could not be assessed due to premature death of these animals). The results showed that isoDGR accumulates in the liver with advancing age, and to a greater extent in Pcmt1^+/−^ mice relative to Pcmt1^+/+^ animals (Fig 5B and D). Pcmt1^+/−^ mice also displayed more extensive liver infiltration of CD68^+^ macrophages than Pcmt1^+/+^ mice (Fig 5C and E), likely due to more rapid decline in Pcmt1 function. Intriguingly, the accumulation of isoDGR‐proteins was also faster (steeper slope from 15 to 24 months, Fig 5D) in older mice (both Pcmt1^+/+^ and Pcmt1^+/−^), suggesting that age exerts a major influence on isoDGR‐dependent pathology. Next, we analyzed inflammatory cytokine levels in plasma from 2‐year‐old Pcmt1^+/+^ and Pcmt1^+/−^ mice. We detected significantly elevated concentrations of pro‐inflammatory mediators in plasma from Pcmt1^+/−^ mice compared to Pcmt1^+/+^ animals (Appendix Fig S6). Together, these results indicate that age‐linked increase in isoDGR‐modified protein levels promotes cytokine release and likely contributes to chronic tissue inflammation in the elderly (Franceschi *et al*, 2018).

### Immune clearance of isoDGR via antibody‐dependent cellular phagocytosis (ADCP)

**Figure 6:**
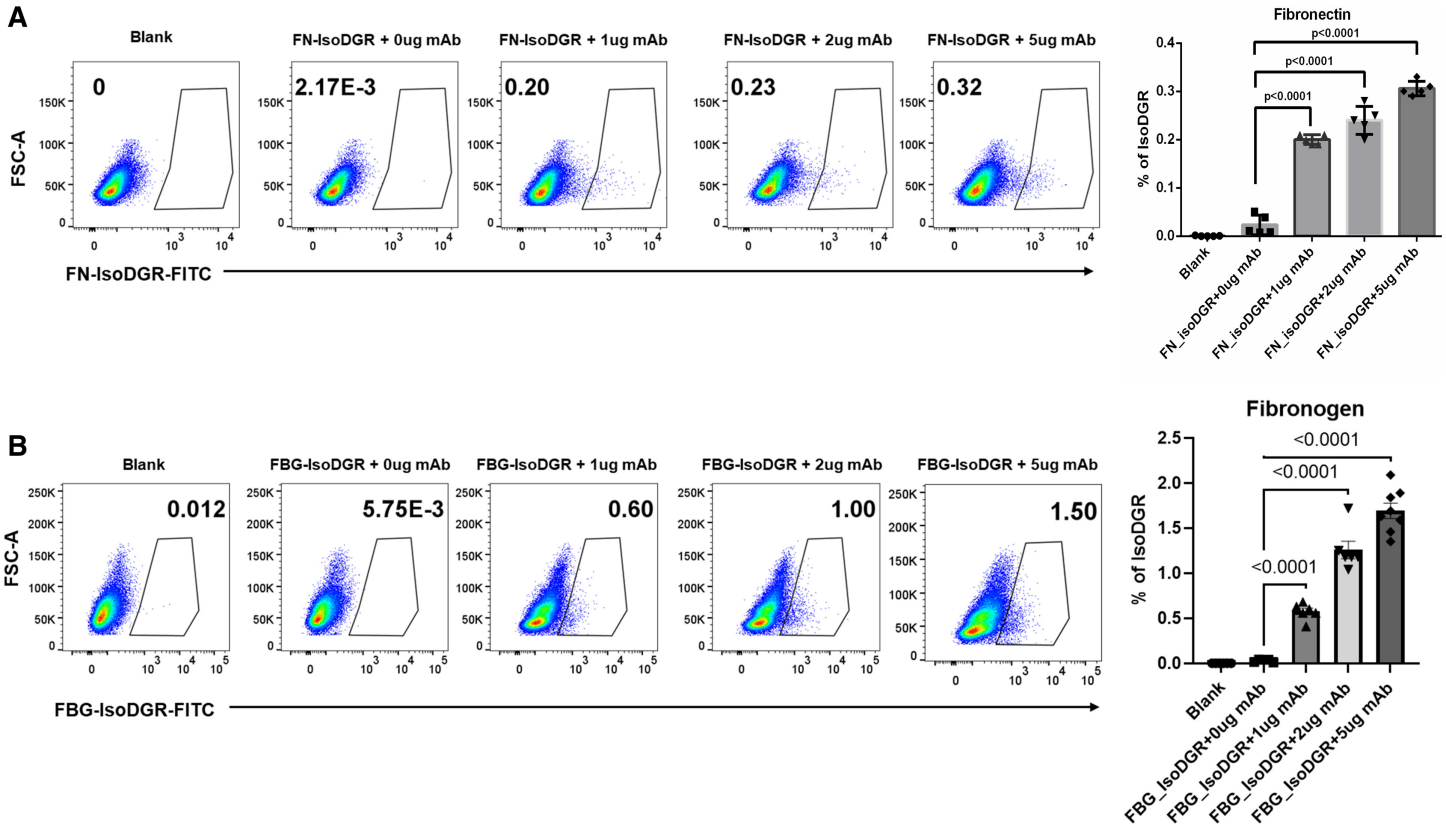
isoDGR‐specific mAb promotes ADCP of isoDGR‐modified fibronectin and fibrinogen Gating strategy used to quantify the number of phagocytic cells containing isoDGR‐modified‐fibronectin‐FITC in the presence of varying concentration mAb treatment (1, 2 or 5 μg/ml). Histogram and bar graph represent the average number of phagocytic RAW macrophages (*n* = 5).Gating strategy used to quantify the number of phagocytic cells containing isoDGR‐modified‐fibrinogen‐FITC in the presence of varying concentration mAb treatment (1, 2 or 5 μg/ml). Histogram and bar graph represent the average number of phagocytic RAW macrophages (*n* = 7). Data information: For all experiments in this figure, *n* = 5–7 independent experiments per condition were performed. We compared five conditions and Kruskal–Wallis test was used to assess statistical significances. Results shown are mean values ± SE. Source data are available online for this figure.

Weekly injection of isoDGR‐specific mAb significantly extended the average lifespan of Pcmt1^−/−^ mice by reducing chronic inflammation. To investigate the mechanism underpinning the therapeutic effects of isoDGR‐mAb, we next used FACS and immunofluorescent imaging to assess whether isoDGR‐mAb could induce immune clearance of damaged proteins by RAW murine macrophages. RAW macrophages were seeded into 24‐well plates and treated for 24 h with 5 μg/ml FITC‐labeled isoDGR‐fibronectin (isoDGR‐FN‐FITC), or native FN‐FITC control, in the presence or absence of isoDGR‐specific mAb (0–5 μg/ml). After 45 min incubation, excess isoDGR‐FN‐FITC was removed by washing with 1× PBS and quenched using trypan blue. FITC‐positive phagocytic cells were then analyzed and quantified by FACS. This analysis revealed a clear mAb dose‐dependent increase in phagocytic activity that was not observed with native FN‐FITC control or in the absence of specific mAb (Fig 6A), thus strongly indicating immune clearance of isoDGR‐antigens via ADCP. Accumulation of isoDGR‐damaged fibrinogen (isoDGR‐FBG) has also been identified in both atherosclerotic tissues and plasma from CVD patients (Dutta *et al*, 2017; Park *et al*, 2021), and we observed a similar mAb dose‐dependent clearance when testing isoDGR‐FBG antigen (Fig 6B). These data indicate that the immune clearance observed was directed against isoDGR rather than being determined by protein identity. Indeed, we were also able to confirm mAb dose‐dependent macrophage clearance of both isoDGR‐FN and isoDGR‐FBG using fluorescence microscopy (Appendix Fig S7).

To confirm that ADCP also contributes to the therapeutic effects of isoDGR‐mAb *in vivo*, we next examined the clearance of FITC‐labeled isoDGR peptide from the lungs of mice treated with motif‐specific antibody or isotype antibody control. We administered FITC‐isoDGR‐peptides intranasally into 12‐week‐old C57BL/6J mice, followed by injection of isoDGR‐mAb or IgG1 isotype control. After 10 min, cells from broncho‐alveolar lavage (BAL) fluid were collected for FACS analysis, which revealed a significantly higher number of F4/80^+^ phagocytic cells in mAb‐treated animals relative to untreated mice or those that received IgG1 isotype control injection (Appendix Fig S8A). To further validate these results, we performed further ADCP assays directly *ex vivo* using 1 × 10^5^ BAL cells seeded into 24‐well plates with 10% RPMI media. The BAL cells were treated with 5 μg/ml FITC‐labeled isoDGR‐fibronectin (isoDGR‐FN‐FITC), then supplemented with isoDGR‐mAb (1 or 5 μg/ml), and incubated for 10 min. In these experiments, we consistently observed a mAb dose‐dependent increase in phagocytic activity that was not detected among cells treated with IgG1 isotype control or in the absence of mAb (Appendix Fig S8B), thus strongly indicating immune clearance of isoDGR‐antigens via ADCP. Taken together, these data strongly suggest that motif‐specific mAb treatment stimulates macrophage clearance of isoDGR‐modified proteins by ADCP, thereby reducing inflammatory cytokine expression in affected body tissues and extending the lifespan of Pcmt1^−/−^ animals.

### Immune clearance of isoDGR reduces inflammatory phenotype of naturally aged C57BL/6 mice

**Figure 7:**
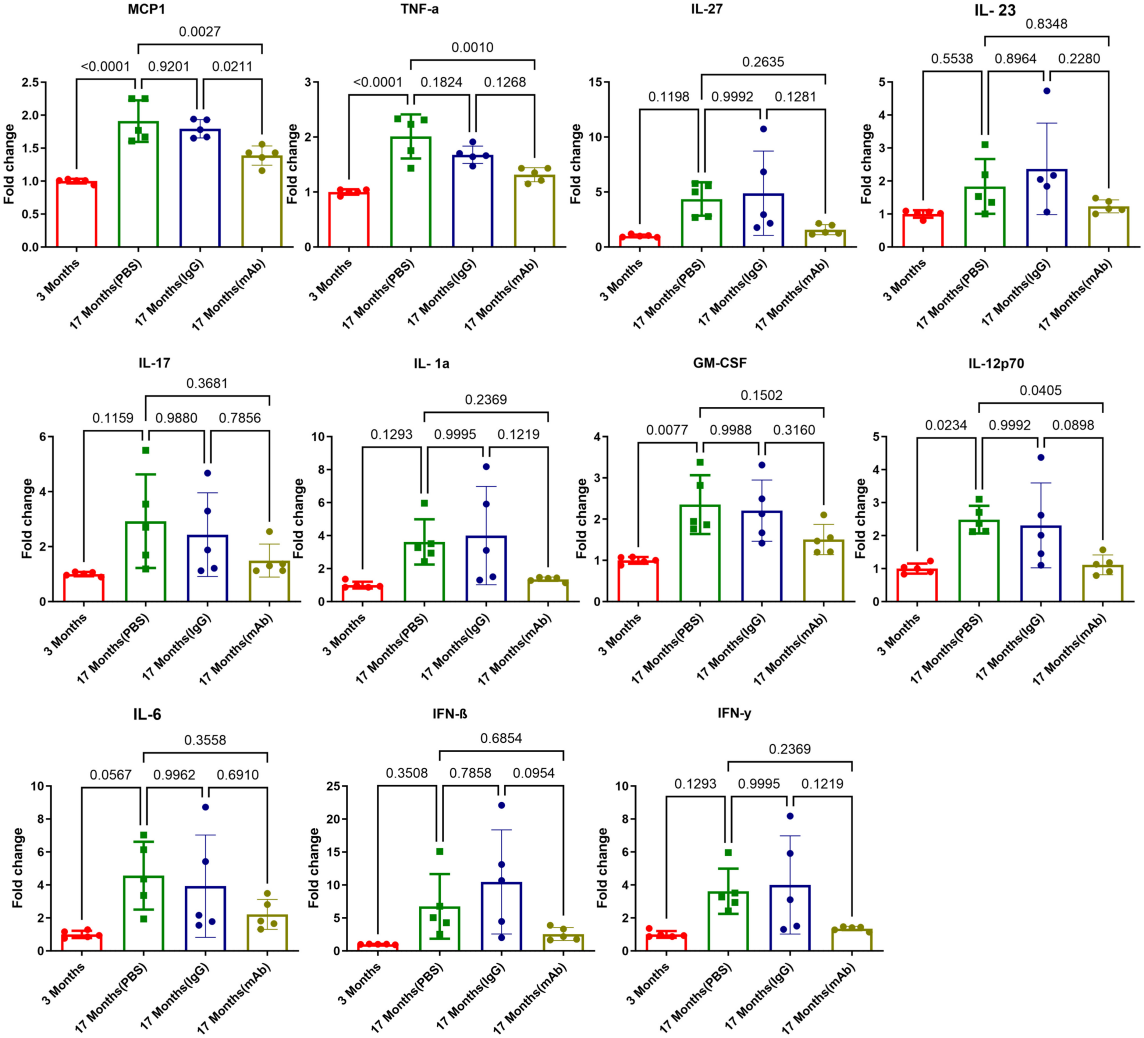
Immune clearance of isoDGR from naturally aging C57BL/6 mice reduced systemic inflammatory cytokines Graph shows quantification of inflammatory cytokines in plasma from 3‐month‐old, 17‐month‐old (PBS), 17‐month‐old (IgG1), 17‐month‐old (mAb) mice (*n* = 5). Data information: In this figure, we used 5 mice per group for all experiments. We compared four groups of mice and assessed statistical significance through one‐way ANOVA. The results are presented as mean values with standard errors (SE). Source data are available online for this figure.

To assess the role of isoDGR in natural age‐induced inflammation, we next compared 3‐ and 17‐month‐old WT C57BL/6J mice treated with isoDGR‐mAb, PBS, or IgG1 isotype control (1 mg/kg specific mAb/isotype or 50 μl PBS was administered weekly for 2 months). Mouse phenotypes were then assessed via plasma cytokine bead assay (Fig 7), tissue isoDGR protein measurement by WB (Appendix Fig S9A) and analyzing immune infiltration of various tissues by immunohistochemistry (Appendix Fig S10–S12). As expected, higher levels of inflammatory cytokines were observed in plasma from 17‐month‐old mice compared to 3‐month‐old mice. Strikingly, pro‐inflammatory cytokine levels in blood plasma from 17‐month‐old mice were markedly reduced upon isoDGR‐mAb treatment, but not following administration of IgG1 isotype control or PBS only (Fig 7, MCP1, TNF‐α, IL‐27, IL‐23, IL‐17, IFN‐β, GM‐CSF, IL‐12p70, IL‐6, IL‐1β, IFN‐γ). These results correlated with isoDGR protein levels detected in body tissues by WB, with significantly higher levels of isoDGR damage being observed in liver from 17‐month‐old mice compared to 3‐month‐old mice. Accordingly, mAb treatment reduced levels of isoDGR‐damaged proteins in liver tissue from 17‐month‐old mice, whereas IgG1 isotype control or PBS injection were unable to replicate these effects (Appendix Fig S9A). Together, these data clearly indicate that mAb treatment reduces isoDGR levels in body tissues, which is further associated with a marked decrease in systemic inflammatory cytokines.

Next, we performed immunohistochemistry for phagocytic cell types in liver and brain tissues from aged mice treated or not with isoDGR‐mAb. We observed that mAb treatment significantly reduced isoDGR protein levels in both liver and brain tissues from 17‐month‐old mice compared to control mice. These results were positively correlated with levels of CD68^+^ (Appendix Fig S10) and F4/80 monocytes‐macrophages infiltrating liver (Appendix Fig S11), and Iba1^+^ microglia in the brain (Appendix Fig S12). Together, these findings suggest that isoDGR‐mAb treatment clears damaged proteins from body tissues which in turn reduces interaction with immune cells, leading to a reduction in inflammatory cytokine levels and decreased tissue inflammation in aged mice.

## Discussion

Tissue aging is underpinned by progressive biomolecular damage that impairs the routine biochemical activities programmed by our genomes (Clarke, 2003; da Costa *et al*, 2016; Hipp *et al*, 2019). Molecular damage to body tissues often results from spontaneous chemical processes, environmental stress, and the generation of reactive metabolites. In particular, non‐enzymatic alterations to protein side chains have been strongly linked with the impairment of routine physiological processes (Gallart‐Palau *et al*, 2015, 2019; Adav & Sze, 2016; Truscott *et al*, 2016). These degenerative protein modifications (DPMs) greatly increase the diversity of biomolecules/proteoforms present in body tissues, including the generation of species capable of mediating novel interactions, rather than simple loss of function (Curnis *et al*, 2006; Corti & Curnis, 2011; Dutta *et al*, 2017). Among these different types of DPM, spontaneous protein deamidation has been proposed to represent “a molecular clock” of tissue aging (Robinson & Robinson, 2001b), but the underlying mechanisms have remained unclear and thus limited the development of interventions that target this DPM.

We previously developed custom proteomic techniques to enable detailed study of global protein deamidation in complex biosamples (Hao *et al*, 2011, 2017; Sze *et al*, 2020). These techniques were used to investigate protein deamidation in vessel walls and plasma from patients with cardiovascular diseases (CVD) and cerebrovascular disease (CeVD). Intriguingly, we found that deamidation of extracellular matrix (ECM) proteins in these tissues confers paradoxical “gain‐of‐function” changes by generating an isoDGR integrin binding sequence, thereby promoting leukocyte adhesion and pro‐inflammatory cytokine expression (Cheow *et al*, 2013; Dutta *et al*, 2017; Park *et al*, 2021). Proteome‐wide sequence analysis also showed that many ECM components are highly enriched in NGR sequences that are susceptible to isoDGR modification. Thus, the risk of isoDGR‐linked diseases will increase exponentially as protein repair functions decline with advancing age.

In the current study, we tested the concept that target‐specific immunotherapy can promote immune clearance of isoDGR‐modified proteins *in vivo*, thereby decreasing tissue inflammation and extending the lifespan of Pcmt1^−/−^ mice (which lack the corresponding isoDGR repair enzyme). To this end, we generated hybridoma cells that produce isoDGR‐specific mAb (Park *et al*, 2021) for use in immunoassays and to investigate the functional consequences of motif accumulation/antibody targeting in both Pcmt1^−/−^ and WT mice. In this study, we observed a marked accumulation of isoDGR and elevated expression of pro‐inflammatory cytokines in multiple body tissues from Pcmt1^−/−^ and naturally aged WT mice. To confirm that isoDGR is responsible for these effects, we also induced deamidation of plasma proteins *in vitro* and showed that these could recapitulate pathology upon injection into young WT mice. Similar data were also obtained when injecting synthetic isoDGR‐peptides into young WT mice. Conversely, weekly injection of 1 mg/kg isoDGR‐mAb was sufficient to induce immune clearance of isoDGR‐modified proteins via ADCP both *in vitro* and *in vivo*. Treatment with isoDGR‐mAb not only doubled lifespan but also preserved behavior/coordination functions, and reduced pro‐inflammatory cytokine levels in the circulation and body tissues. Together, these results indicate that mAb targeting of age‐damaged proteins is a viable immunotherapeutic approach that could have wide‐ranging implications for the treatment of chronic diseases prevalent in elderly populations.

Time‐dependent biomolecular damage caused by DPMs (e.g. oxidation, deamidation, glycation, carbamylation) is well‐established to mediate human aging and promote degenerative diseases. To date, the majority of research into combating age‐related molecular damage has focused on the overexpression of specific repair enzymes or restricting caloric intake to decrease levels of reactive metabolites. Our study clearly indicates that immunotherapeutic targeting of specific DPMs can trigger immune clearance of age‐damaged proteins to reduce pathology and extend lifespan. We anticipate that future research in this area will uncover additional DPM‐induced mechanisms of biological aging, as well as stimulate the development of novel immune‐mediated and other therapies that target DPMs to increase human healthy lifespan.

## Materials and Methods

### Animal experiments

All mouse procedures were conducted in a humane manner and were approved by the NTU Institutional Animal Care and Use Committee (IACUC protocol # ARF‐SBS/NIE/LKC‐A18016, A19029, A18059) or the Animal Care Committee at Brock University (AUP # 22‐08‐04). The experiments strictly adhered to the international guiding principles for animal research and followed guidelines on the care and use of laboratory animals for scientific purposes. In this study, animals were randomly assigned to treatment and control groups. However, blinding of animal groups was not implemented as the experimental design did not involve blinding procedures.

Mice deficient in deamidation repair enzyme Pcmt1 were previously generated by Clarke and co‐worker (Kim *et al*, 1999). We obtained Pcmt1^+/−^ (C57BL/6 background) mice from the Jackson Laboratory (B6;129S4‐Pcmt1tm1Scl/J; Strain #:023343; RRID:IMSR_JAX:023343). Pcmt1^+/−^ (male and female) mice were bred to yield litters comprising Pcmt1^+/+^, Pcmt1^+/−^, and Pcmt1^−/−^ offspring in the expected Mendelian ratios. Mouse genotype was confirmed by PCR using the primers as shown in Table S1. Mice were maintained on normal chow diets and housed with regular light/dark cycles.

### Cell culture

The RAW 264.7 (ATCC® TIB‐71™) cell line was acquired from ATCC. Cells were maintained in complete DMEM high glucose medium supplemented with 10% FBS and 1% penicillin/streptomycin, in an atmosphere of 5% CO2 and 95% humidity at 37°C. Cells were passaged when they reached 90% confluence, detached with a cell scraper, and subcultured at a 1:4 ratio in T‐75 flasks. Cells were continuously cultured from the 2^nd^ passage until passage number 13. Cells were frozen every fifth passage starting from the third passage. The phagocytosis experiment has been done within 5–13 passages. Regular testing for Mycoplasma contamination was conducted. To maintain consistency in cell maintenance and phagocytosis assay done by a single individual.

### Anti‐isoDGR immunotherapy

The monoclonal antibody specifically developed to target (L‐isoD)GR motif had been previously reported (Park *et al*, 2021). Pcmt1^−/−^ pups were intraperitoneally (i.p.) injected with 1 mg/kg/week mAb starting from 1 week old until use in the study. Age‐matched control animals were administered an equal volume of 1× PBS only. All mice were fed a normal laboratory diet (Altromin 1320M, Germany) from weaning onwards, body weight was measured weekly, and all animals were included in the survival analyses. Motor functional (hind‐limb clasping test and ledge test) and behavioral functions were analyzed at 6 weeks. Blood was collected to prepare plasma for cytokine measurement.

### Anti‐isoDGR immunotherapy in naturally aged WT C57BL/6J mice

WT C57BL/6J mice at 17 months of age (catalog number: 000664) were obtained from the Jackson Laboratory in Bar Harbor, ME, USA. Animals were housed under specific pathogen‐free (SPF) conditions in isolator cages for 2 weeks prior to the start of the experiments. The animal house maintained a room temperature of 24°C with 12 h light/dark schedule. Mice were provided access to Purina rodent chow diet and tap water *ad libitum*.

The mice were divided into four study groups, each containing a minimum of 5 animals. Group 1 consisted of 3‐month‐old young mice (bred in‐house), whereas groups 2–4 were 17‐month‐old mice. Group 2 received an i.p. injection of 50 μl PBS only, group 3 received an i.p. injection of 1 mg/kg/50 μl isoDGR‐mAb, and group 4 received an i.p. injection of 1 mg/kg/50 μl IgG1 isotype control. The isoDGR‐mAb, IgG1, or PBS injections were administered once per week to the respective groups for 2 months.

After the 2‐month treatment period with mAb or controls (IgG1 and PBS), blood samples were collected from each group. The blood was centrifuged at 1,500 x *g* to separate the plasma, which was then analyzed to evaluate the effect of mAb on systemic inflammatory cytokines using a LEGENDplex™ multiplex bead assay. Following these experiments, mice were deep anesthetized using ketamine/xylazine and then perfused with 20 ml of 1× PBS to remove blood from body tissues. The liver and brain were collected from all groups of mice for subsequent immunohistochemistry and Western blot analysis.

### Hind‐limb clasping test

Mice were suspended by their tails and the extent of hindlimb clasping was observed for 30 s. If both hind‐limbs were splayed outward away from the abdomen with spread toes, a score of 0 was given. If one hind‐limb was fully retracted or both hind‐limbs partially retracted without touching the abdomen and with toes spread, a score of 1 was assigned. If both hind‐limbs were partially retracted and in contact with the abdomen but without touching each other, a score of 2 was given. If both hind‐limbs were fully clasped and touching the abdomen, a score of 3 was assigned.

### Ledge test

The ledge test for evaluating balance and coordination was carried out as previously described, with a minor modification. Mice were placed on a ledge (90 cm long, 0.5 cm wide, 20 cm high), and paw placement and forward movement were observed. Time taken to cross the ledge and the number of paw slips were recorded. Mice that left the ledge were excluded.

### IsoDGR‐antigen uptake/ADCP assays

Antibody‐dependent cellular phagocytosis assays were performed by fluorescence microscopy and FACS. A total of 5 × 10^5^ murine RAW macrophages were seeded into 24‐well plates and treated with 5 μg/ml FITC‐labeled isoDGR‐fibronectin (isoDGR‐FN‐FITC), FITC‐labeled isoDGR fibrinogen (isoDGR‐FG‐FITC), or native FN‐FITC/FG‐FITC control for 24 h (Dutta *et al*, 2017). After 45 min incubation with varying doses of isoDGR‐mAb (0–5 μg/ml), excess fluorescence was quenched by adding trypan blue and incubating for 10 min. The cells were then washed 3 times and re‐suspended in PBS. The mAb dose‐dependent uptake of isoDGR‐antigen by RAW cells was determined by FACS using FlowJo software to quantify phagocytic cells. To confirm the FACS results, cells were also examined by fluorescent imaging using a Zeiss LSM710 confocal microscope. The fluorescence intensity of each cell was calculated using Image J software.

### Histological assessment and immunostaining of liver and brain tissues

Tissues from *Pcmt1* ^+/+^, *Pcmt1* ^+/−^, *Pcmt1* ^−/−^, and mAb‐treated *Pcmt1* ^−/−^ mice were collected and fixed with 4% PFA at 4°C for 24 h. The tissues were washed with 1× PBS and transferred into 15% sucrose, followed by 30% sucrose, then stored at 4°C. Tissue was embedded in OCT compound with dry ice and cut into 10 μm sections using a Leica CM3060S Cryostat. The sections were mounted onto Fisherbrand Superfrost Plus microscope slides and kept in warm PBS for 20 min to remove OCT prior to staining with hematoxylin and eosin. For immunostaining, slides were permeabilized with 0.5% PBST for 2–3 h and then incubated with blocking buffer (2.5% normal goat serum, 1% BSA in 0.5% PBST) for 1 h at RT prior to the addition of primary antibodies (isoDGR [1:200] and CD68 [1:200, Abcam, ab283654]) overnight at 4 °C. Slides were next washed with PBS (3×) for 5 min then incubated with secondary antibodies conjugated to AlexaFluor 488 and 594 (1:500) for 1 h at RT. The slides were again washed with PBS (3×) for 5 min, then incubated with DAPI for 15 min to visualize cell nuclei. After staining, slides were washed with 1× PBS and mounted with aqueous mounting media. Images were acquired using a Zeiss LSM710 confocal microscope. The readings were acquired by Image J software using images from *n* = 5 mice. The software randomly samples 50 readings in different locations per image.

### Measurement of plasma cytokine levels

The LEGENDplex™ multiplex bead assay/mouse inflammation panel (Biolegend, San Diego, CA), was used to measure 13 different cytokines (IL23, IL1α, IL1β, IL6, IL10, IL12p70, IL17A, IL23, IL27, MCP1, IFNβ, IFNγ, TNFα, and GMCSF) in blood plasma from 6‐week‐old Pcmt1^+/+^, Pcmt1^+/−^, Pcmt1^−/−^ and mAb‐treated Pcmt1^−/−^ mice as well as 17‐week‐old WT mice treated or not with isoDGR‐mAb (assessed by LSRII flow cytometer according to the manufacturer's protocol).

### Real‐time PCR

Total RNA isolated from tissues was treated with DNase and reverse‐transcribed using a first‐strand DNA synthesis kit from Invitrogen. The PCR was performed on an ABI Fast 7500 System (Applied Biosystems, Foster City, CA). TaqMan probes for the respective genes were custom‐generated by Applied Biosystems based on the sequences in the Illumina array and used as per the manufacturer's instructions. Expression levels of target genes were determined in triplicate from the standard curve and normalized to GAPDH mRNA level. The primer sets used are showed in Table S2.

### Western blot analysis

Western blot analysis was performed using standard methods. Primary antibodies and dilutions were as follows: isoDGR (mouse monoclonal 1:1,000), Pcmt1 (rabbit polyclonal, Abcam 1:1,000), GAPDH (Invitrogen 1:1,000).

### Statistics

Statistical analyses were performed using GraphPad Prism v9.0 (GraphPad Software, Inc., San Diego, CA). Kaplan–Meier survival differences between experimental groups were evaluated using Mantel‐Cox log‐rank test. Data were tested for normality using D'Agostino & Pearson, or Shapiro–Wilk tests. For normally distributed data, differences between groups were assessed either by two‐tailed unpaired Student's *t*‐test or one‐way ANOVA for multiple group analyses, followed by Tukey's multiple comparisons test. Otherwise, Kruskal–Wallis test with Dunn's multiple comparisons *post hoc* test was used. *P* < 0.05 was considered significant.

## Author contributions

**Siu Kwan Sze:** Conceptualization; resources; supervision; funding acquisition; writing – original draft; project administration; writing – review and editing. **Pazhanichamy Kalailingam:** Data curation; formal analysis; validation; investigation; visualization; methodology; writing – original draft; writing – review and editing. **Khalilatul‐Hanisah Mohd‐Kahliab:** Data curation; formal analysis; methodology. **SoFong Cam Ngan:** Data curation; methodology. **Ranjith Iyappan:** Data curation; methodology. **Evelin Melekh:** Methodology. **Tian Lu:** Methodology. **Gan Wei Zien:** Data curation; methodology. **Bhargy Sharma:** Data curation; methodology. **Tiannan Guo:** Resources; software. **Adam J MacNeil:** Resources; software. **Rebecca EK MacPherson:** Resources; software; funding acquisition. **Evangelia Litsa Tsiani:** Resources; funding acquisition. **Deborah D O'Leary:** Resources; funding acquisition; writing – review and editing. **Kah Leong Lim:** Resources; funding acquisition. **I Hsin Su:** Resources; software; writing – review and editing. **Yong‐Gui Gao:** Resources; funding acquisition; writing – review and editing. **A Mark Richards:** Resources; software; writing – review and editing. **Raj N Kalaria:** Resources; funding acquisition; writing – review and editing. **Christopher P Chen:** Resources; software; funding acquisition; writing – review and editing. **Neil E McCarthy:** Resources; software; funding acquisition; writing – review and editing.

## Disclosure and competing interests statement

The authors declare that they have no conflict of interest.

## For more information

- https://brocku.ca/applied‐health‐sciences/health‐sciences/faculty‐research/faculty‐directory/newman‐sze/.
- https://scholar.google.com/citations?user=Y7qo7dMAAAAJ&hl=en.

## Supporting information



Appendix

Source Data for Appendix

Source Data for Figure 1

Source Data for Figure 2

Source Data for Figure 3

Source Data for Figure 4

Source Data for Figure 5

Source Data for Figure 6

Source Data for Figure 7

## Data Availability

All data presented in this study are included in the main text or in the appendix. This study includes no data deposited in external repositories.
