# Supplementary material for: Immunotherapy targeting isoDGR‐protein damage extends lifespan in a mouse model of protein deamidation
Source: EMBO Mol Med. 2023 Nov 16;15(12):e18526. doi: 10.15252/emmm.202318526 (PMC10701600; doi:10.15252/emmm.202318526)
Supplement: Supplementary file 1 — Appendix [file EMMM-15-e18526-s001.pdf]

Appendix

Antibody Targeting of Age-Linked isoDGR Protein Damage Mitigates Lifespan Reduction in a Mouse Model of Chronic Inflammation

Pazhanichamy Kalailingam, Khalilatul-Hanisah Mohd-Kahliab, SoFong Cam Ngan, Ranjith Iyappan, Evelin Melekh, Tian Lu, Gan Wei Zien, Bhargy Sharma, Tiannan Guo, Adam J. MacNeil, Rebecca E. K. Macpherson, Evangelia Litsa Tsiani, Deborah D. O'Leary, Kah Leong Lim, I Hsin Su, Yong-Gui Gao, A Mark Richard, Raj N. Kalaria, Christopher P. Chen, Neil E. McCarthy and Siu Kwan Sze

Table of Contents

Appendix Figure S1: Increased isoDGR levels and correlation with CD68+ macrophages in Pcmt1-/- mouse spleen in response to mAb treatment. 2

Appendix Figure S2: Increased isoDGR levels and correlation with F4/80+ macrophages in Pcmt1-/- mouse spleen in response to mAb treatment. 3

Appendix Figure S3: Increased isoDGR levels/co-localization with CD68+ macrophages in Pcmt1-/- mouse thymus in response to mAb treatment. 4

Appendix Figure S4: IsoDGR-modified plasma proteins induce systemic inflammation in young wild-type C57BL/6J mice. 5

Appendix Figure S5: IsoDGR-peptides promote inflammatory cytokine release in young wild-type C57BL/6J mice. 6

Appendix Figure S6: Partial deletion of Pcmt1 leads to systemic elevation of proinflammatory cytokines in old mice. 7

Appendix Figure S7: Target-specific mAb enhances phagocytosis of isoDGR-modified fibronectin. 8

Appendix Figure S8: isoDGR-specific mAb promotes in vivo and ex vivo ADCP of isoDGR peptides 9

Appendix Figure S9: Anti-isoDGR mAb treatment reduces motif level in brain and liver from Pcmt1-/- mice. 10

Appendix Figure S10: IsoDGR positive correlation with CD68+ monocyte-macrophages in liver from 17 month C57BL/6J mice. 11

Appendix Figure S11: mAb treatment reduces the F4/80 macrophages in liver of aged 17 month C57BL/6J mice. 12

Appendix Figure S12: mAb treatment reduces the size of cellular body of microglia in aged WT mice brain. 13

Appendix Figure S13: Full WB images for Figure 1A. 14

Table S1 Primers used in genotyping mouse Pcmt1 gene. 15

Table S2 Primers used for quantitative RT-PCR analysis of mouse genes, related to Figure 4A. 15

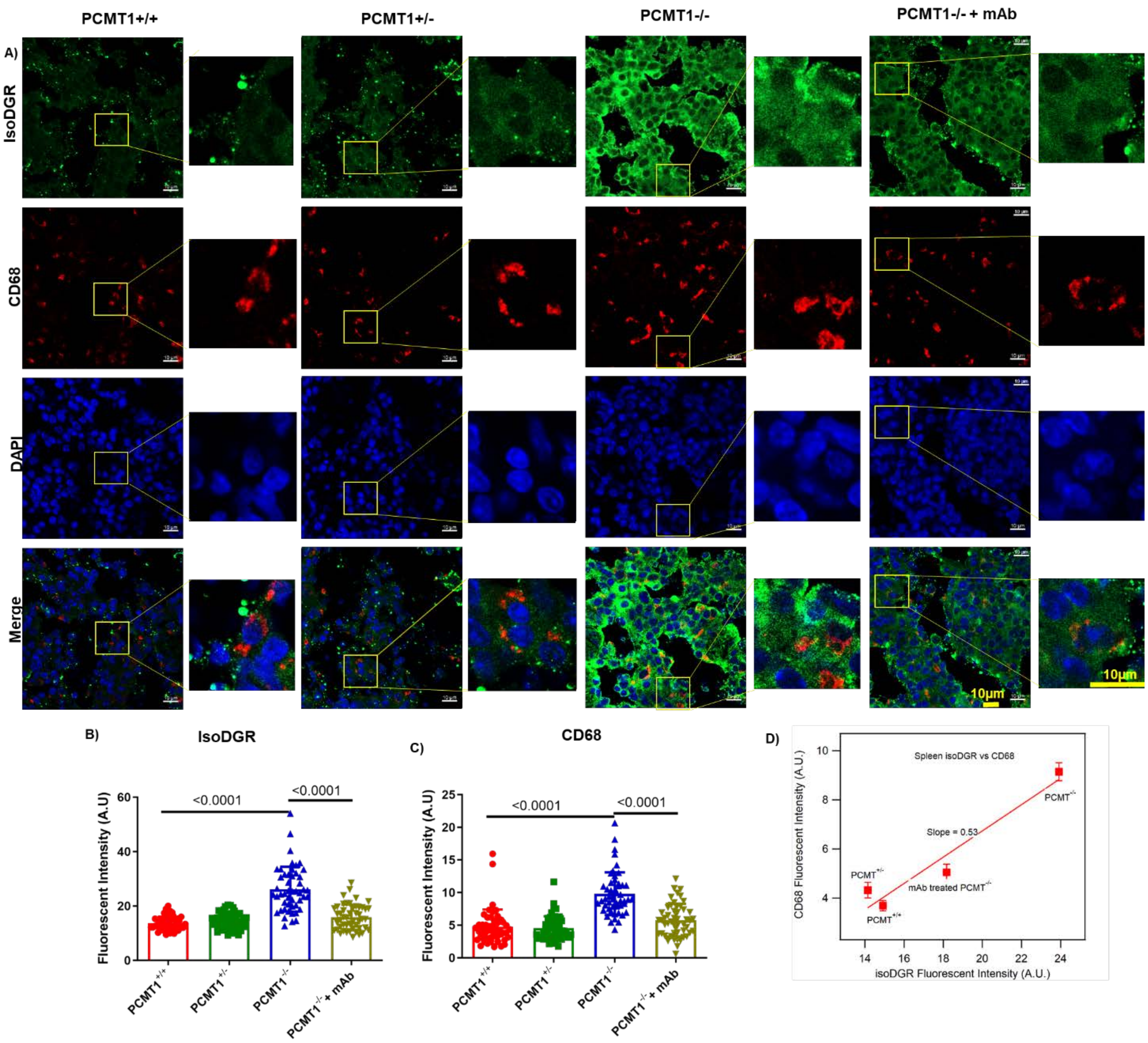

Appendix Figure S1: Increased isoDGR levels and correlation with CD68+ macrophages in Pcmt1-/- mouse spleen in response to mAb treatment.

(A) Representative immunostaining images showing isoDGR distribution and positive correlation with CD68+ macrophages in cryosectioned spleen tissue (Pcmt1$^{+/+}$, Pcmt1$^{+/-}$, Pcmt1$^{-/-}$, and mAb-treated Pcmt1$^{-/-}$ mice at 6 weeks). (B) IsoDGR or (C) CD68 fluorescence in 50 randomized regions from 6 images of 6 independent spleen sections for each genotype were quantified using image J (graphs shows averaged values for the same region from 3 images). (D)Plot showing the CD68 is proportionally increased with isoDGR accumulation with slope=0.55, suggesting spleen is highly sensitive to aging-damaged isoDGR-molecules.

Data information: In this figure, we utilized 6 mice per condition for all experiments. We randomly selected 50 regions from 6 images for statistical analysis. Statistical significance was assessed using the one-way ANOVA. Results shown are mean values ± SE.

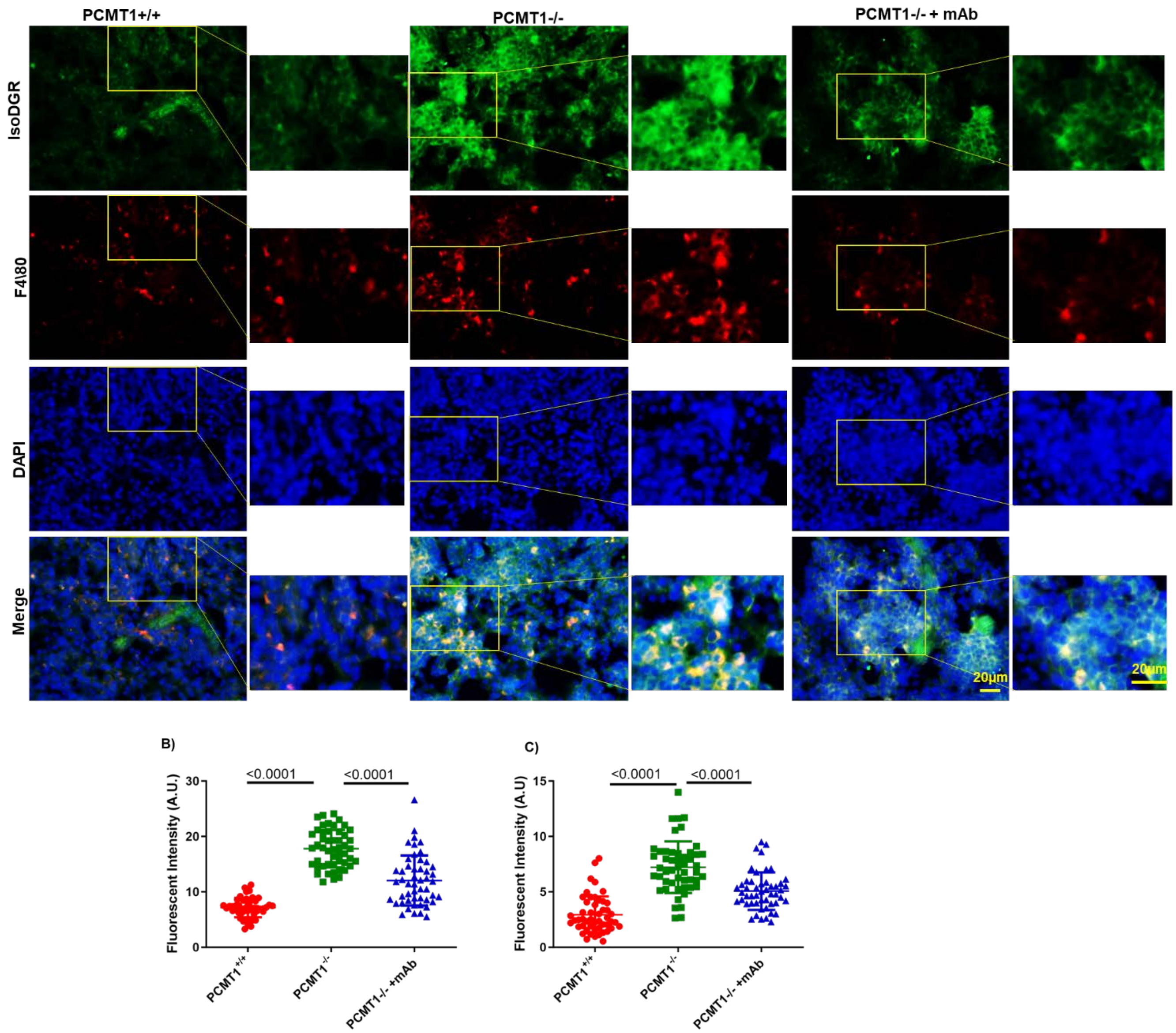

Appendix Figure S2: Increased isoDGR levels and correlation with F4/80+ macrophages in Pcmt1-/- mouse spleen in response to mAb treatment.

(A) Representative immunostaining images showing isoDGR distribution and co-localisation with F4/80+ macrophages in cryosectioned spleen tissue (Pcmt1$^{+/+}$, Pcmt1$^{-/-}$, and mAb-treated Pcmt1$^{-/-}$ mice at 6 weeks). (B) IsoDGR or (C) F4/80+ fluorescence in 50 randomized regions from 6 images of 6 independent spleen sections for each genotype were quantified using image J (graphs shows averaged values for the same region from 3 images).

Data information: In this figure, we utilized 6 mice per condition for all experiments. We randomly selected 50 regions from 6 images for statistical analysis. Statistical significance was assessed using the one-way ANOVA. Results shown are mean values ± SE.

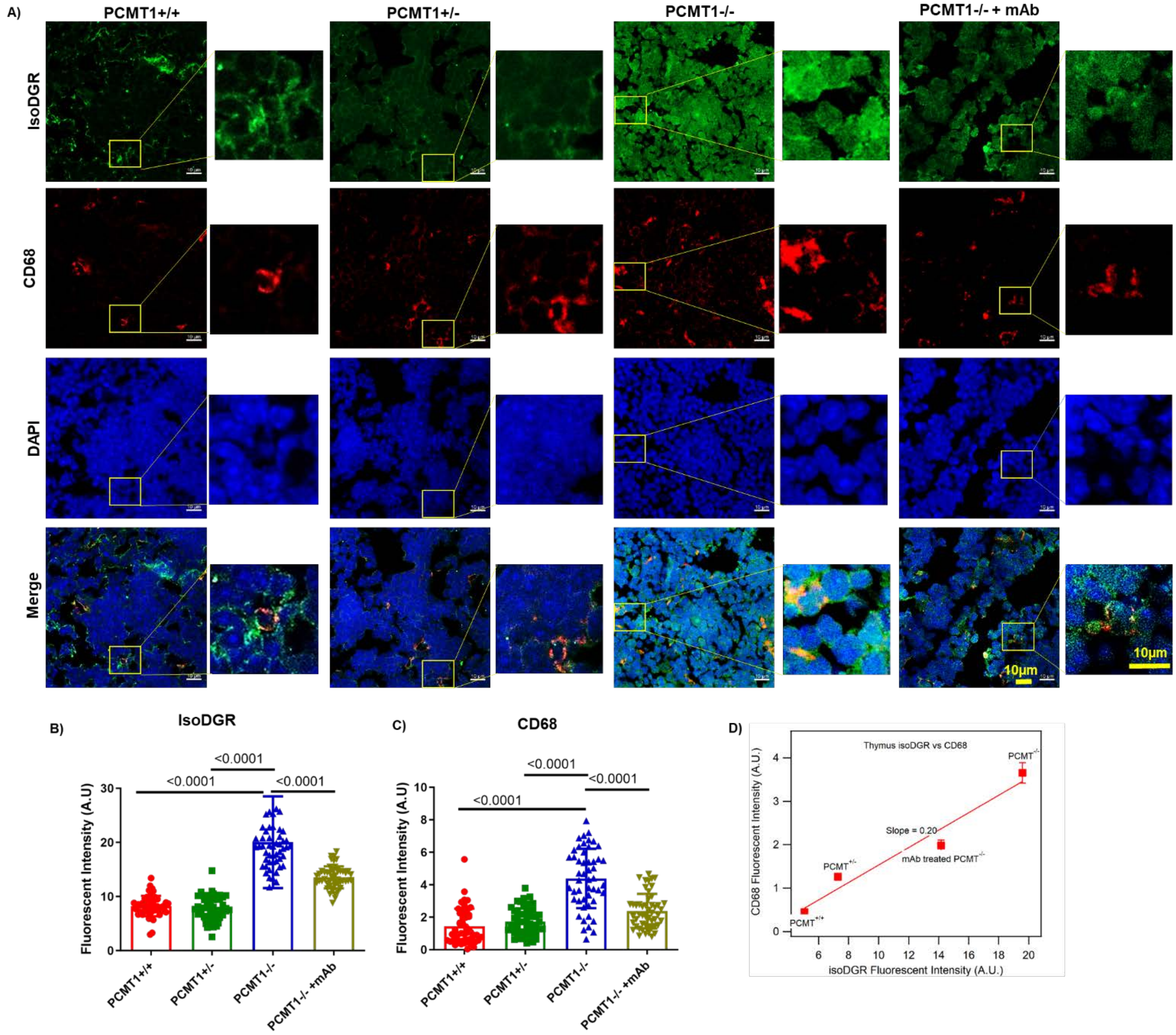

Appendix Figure S3: Increased isoDGR levels/co-localization with CD68+ macrophages in Pcmt1-/- mouse thymus in response to mAb treatment.

(A) Representative immunostaining images showing isoDGR distribution and positive correlation with CD68+ macrophages in cryosectioned thymus tissue (Pcmt1$^{+/+}$, Pcmt1$^{+/-}$, Pcmt1$^{-/-}$, and mAb-treated Pcmt1$^{-/-}$ mice at 6 weeks). (B) IsoDGR or (C) CD68 fluorescence in 50 randomized regions from 5 images of 5 independent thymus sections for each genotype were quantified using image J (graphs shows averaged values for the same region from 3 images). (D)Plot showing the CD68 is proportionally increased with isoDGR accumulation with slope=0.20.

Data information: In this figure, we utilized 5 mice per condition for all experiments. We randomly selected 50 regions from 5 images for statistical analysis. Statistical significance was assessed using the Kruskal-Wallis test. Results shown are mean values ± SE.

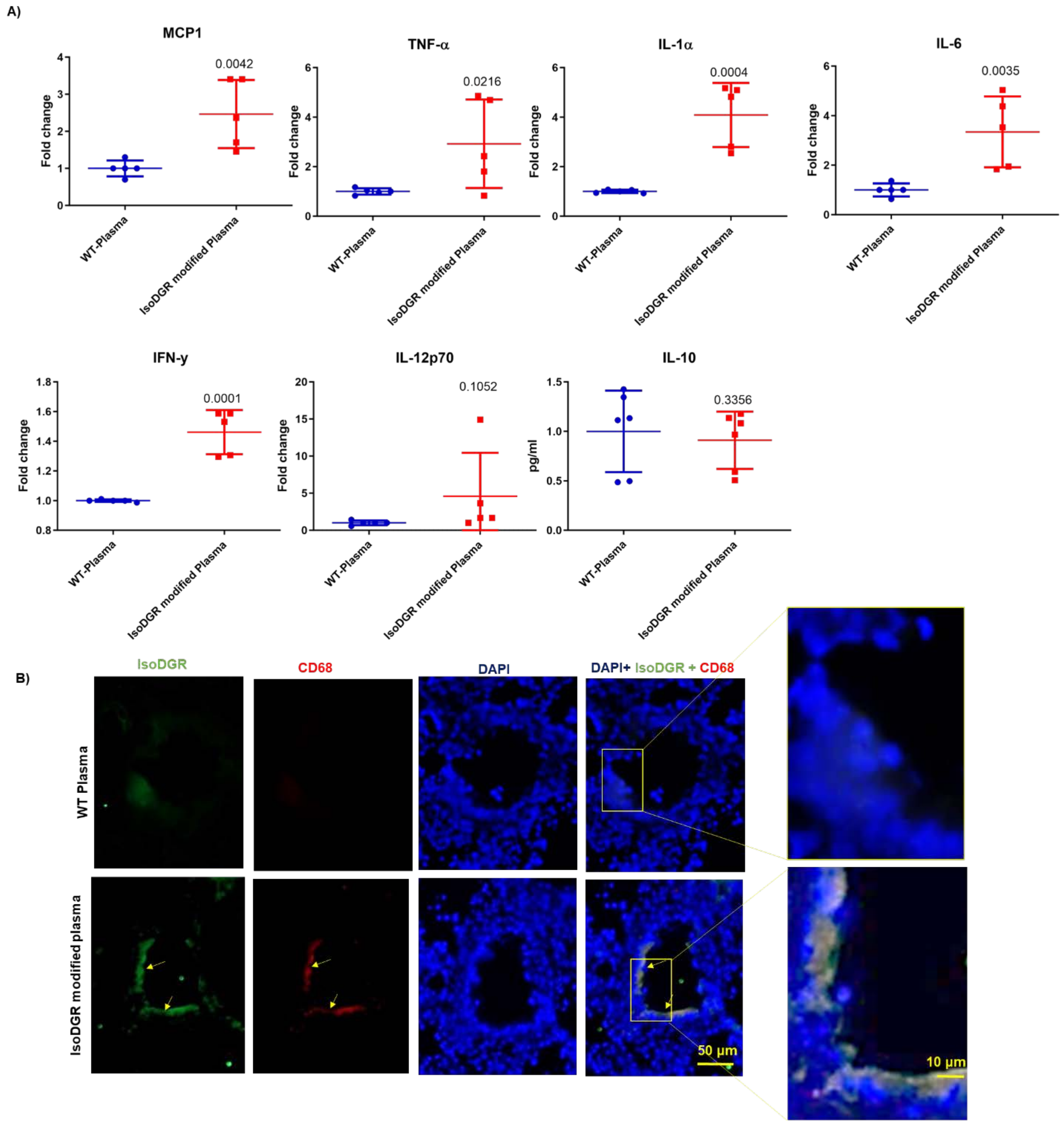

Appendix Figure S4: IsoDGR-modified plasma proteins induce systemic inflammation in young wild-type C57BL/6J mice.

(A) Graph shows quantitation of blood plasma cytokines in 6-week-old WT mice injected with unmodified plasma or isoDGR-modified plasma (n=6). (B) Representative immunostaining images showing isoDGR protein distribution and co-localisation with CD68+ macrophages in cryosectioned lung small vessels from WT mice treated with WT or isoDGR-modified plasma.
Data information: In this figure, we used 5 mice per group. We compared two groups of mice and assessed statistical significance using unpaired, one-tailed Student's t-test. The results are presented as mean values with standard errors (SE).

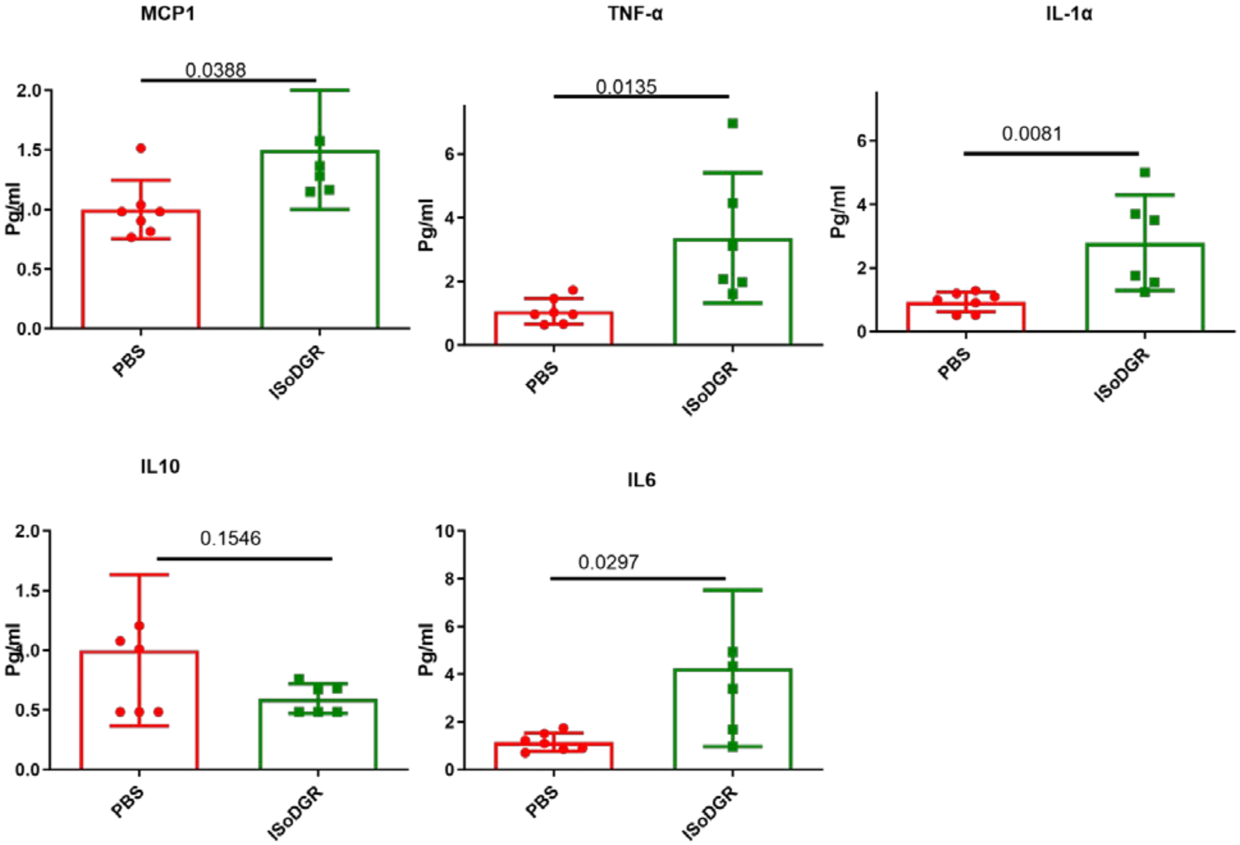

Appendix Figure S5: IsoDGR-peptides promote inflammatory cytokine release in young wild-type C57BL/6J mice.

Graph shows quantitation of blood plasma chemokines / cytokines MCP1, TNF-α, IL-1α, IL-10 and IL-6 in 6-week-old C57BL/6J mice treated with either isoDGR synthetic peptide(n=6) or PBS-only control (n=7).

Data information: Statistical significances were calculated by one-way ANOVA. Results shown are mean values ± SE.

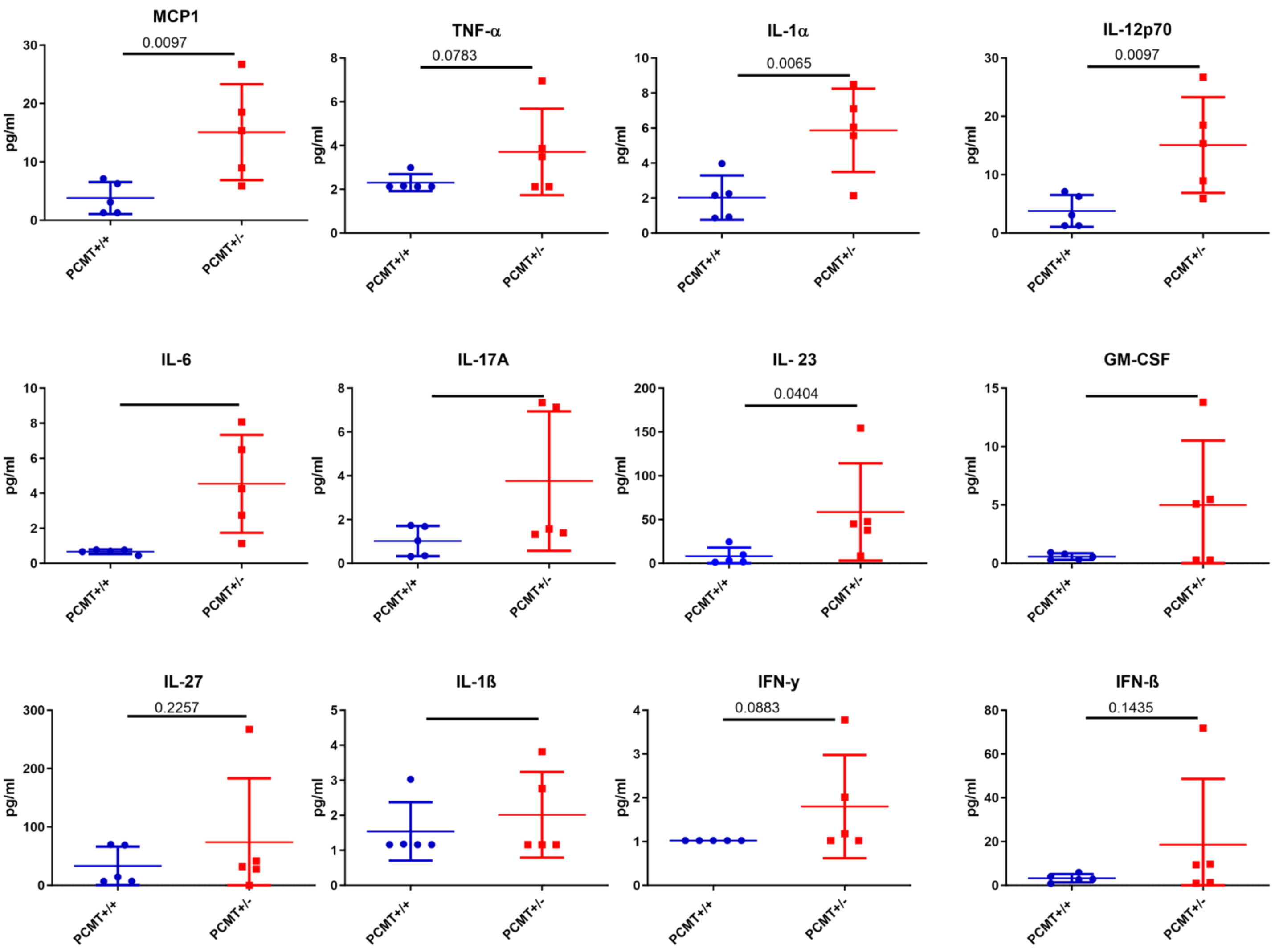

Appendix Figure S6: Partial deletion of Pcmt1 leads to systemic elevation of proinflammatory cytokines in old mice.

Graph shows quantification of inflammatory cytokines in plasma from 2-year-old Pcmt1$^{+/+}$ and Pcmt1$^{+/-}$ mice (n=5).

Data information: Statistical significances were assessed by one-way ANOVA. Results shown are mean values ± SE.

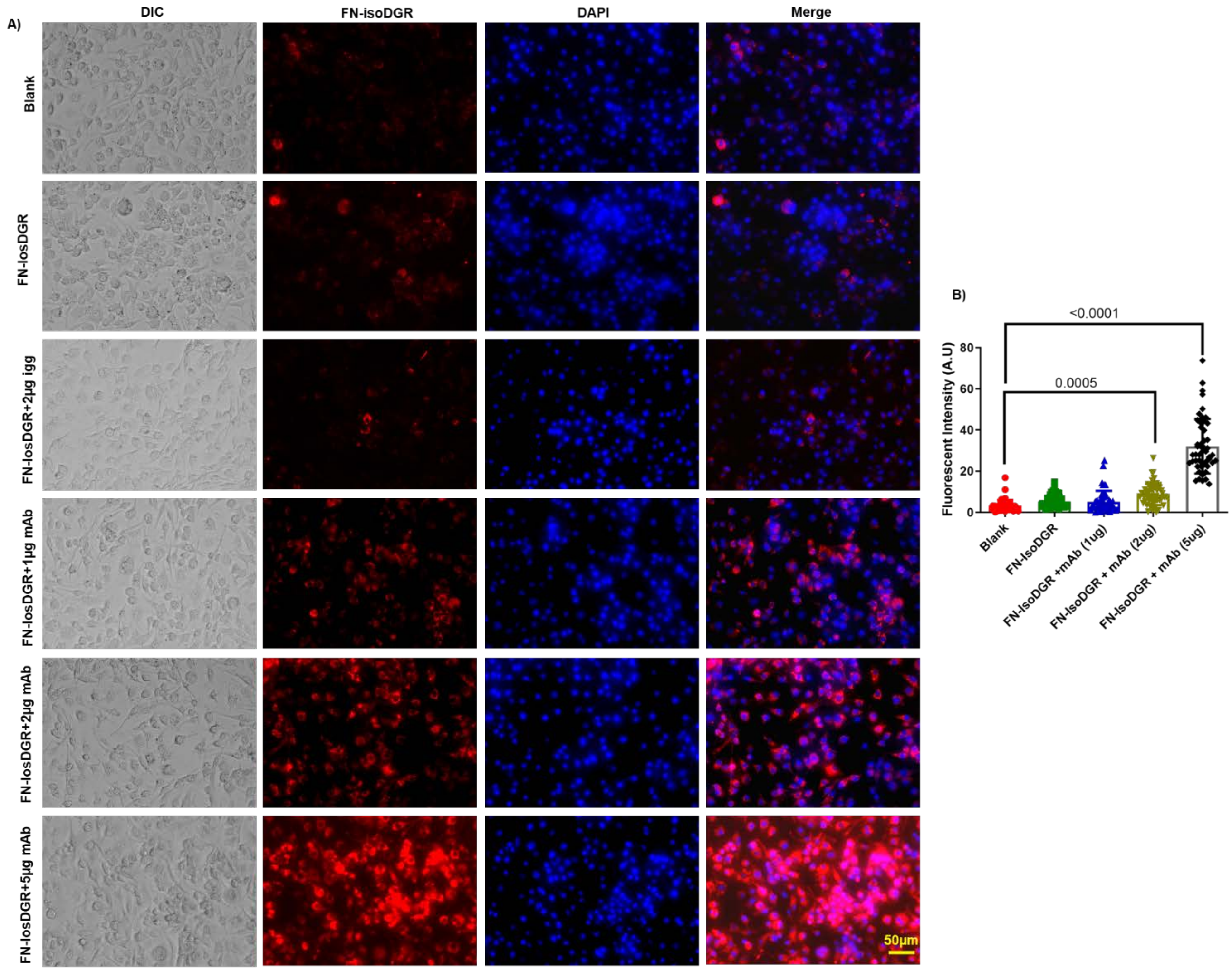

Appendix Figure S7: Target-specific mAb enhances phagocytosis of isoDGR-modified fibronectin.

Representative immunostaining images showing that phagocytosis of isoDGR-FN by RAW macrophages increases with motif-specific mAb concentration (1-5µg/ml). Bar graph shows average number of phagocytic RAW macrophages.

Data information: Fluorescent signal in 50 random regions taken from 5 images in 5independent experiments were quantified by image J software (graph shows averaged values). Kruskal-Wallis test was used to calculate statistical significances. Results shown are mean values ± SE.

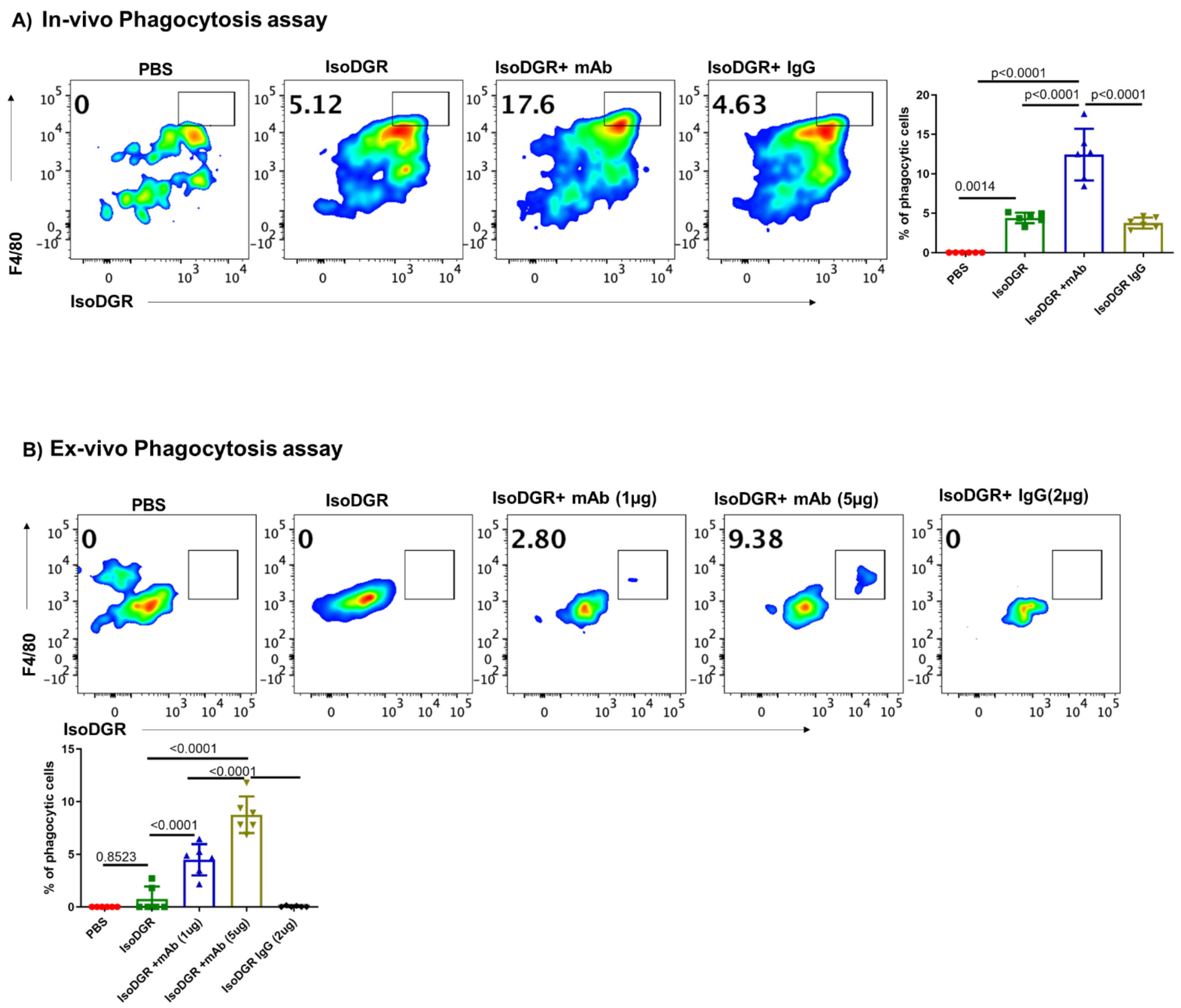

Appendix Figure S8: isoDGR-specific mAb promotes in vivo and ex vivo ADCP of isoDGR peptides.

(A) Gating strategy used to quantify the number of phagocytic cells containing FITC-peptides with isoDGR-mAb treatment in the lung broncho-alveolar lavage fluids. Histogram and bar graph represent the average number of phagocytic F4/80+ macrophages (n=6). (B) Gating strategy used to quantify the number of phagocytic cells containing isoDGR-modified-fibrinogen-FITC in the presence of varying concentration mAb treatment (1 or 5µg/ml).

Data information: Histogram and bar graph represent the average number of phagocytic F4/80+ macrophages (n=6). Kruskal-Wallis test was used to assess statistical significances. Results shown are mean values ± SE.

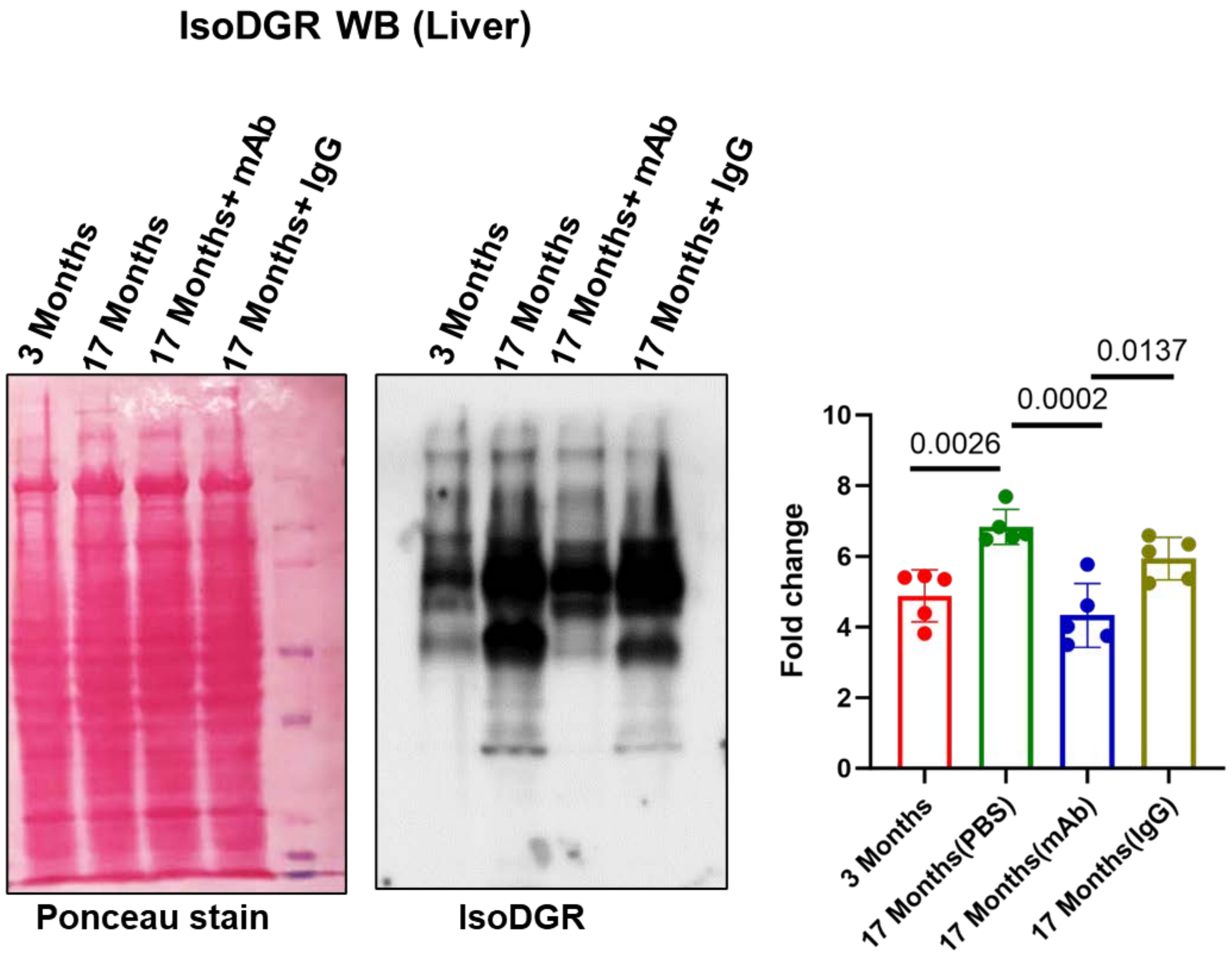

Appendix Figure S9: Anti-isoDGR mAb treatment reduces motif level in brain and liver from Pcmt1-/- mice.

Protein lysates from liver of 3 month, 17 months(PBS), 17 months(mAb) 17 months(IgG) mice were subjected to western blot using isoDGR-specific antibody. Protein loadings were visualized by Ponceau S.

Data information: Graphs show quantification of isoDGR-modified protein levels in mouse liver. Statistical significances were determined by 1-way ANOVA.

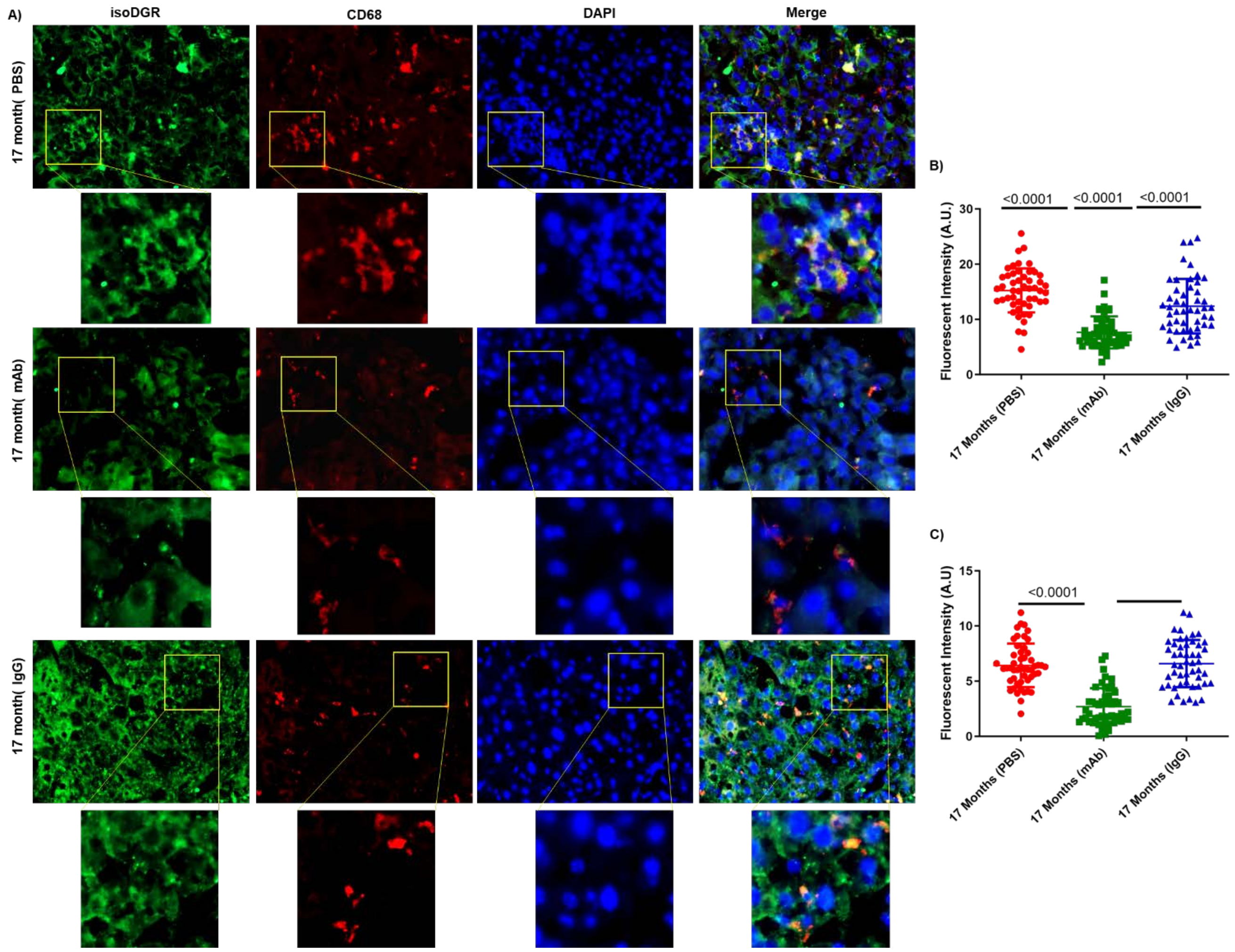

Appendix Figure S10: IsoDGR positive correlation with CD68+ monocyte-macrophages in liver from 17 month C57BL/6J mice.

(A) Representative immunostaining images showing isoDGR distribution and correlation with CD68+ macrophages in cryosectioned liver tissue (17months(PBS), 17months(mAb) 17months(IgG), n=5). (B) IsoDGR or (C) CD68.

Data information: Fluorescent signal in 50 randomized regions from 5 images of 5 independent spleen sections for each genotype were quantified using image J (graphs show averaged values for the same region from 5 images). Statistical significances were determined by Kruskal-Wallis test. Results shown are mean values ± SE.

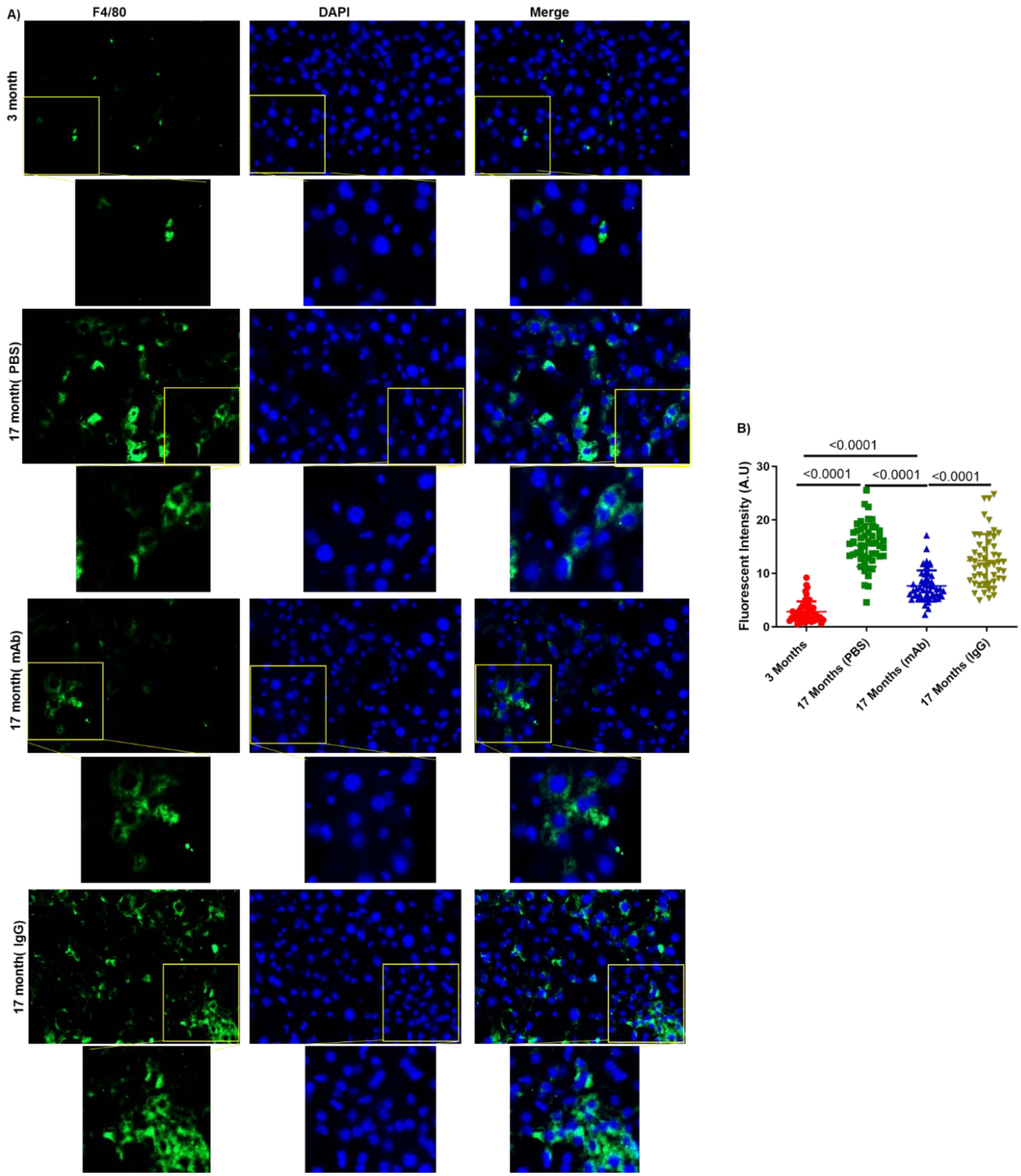

Appendix Figure S11: mAb treatment reduces the F4/80 macrophages in liver of aged 17 month C57BL/6J mice.

(A) Representative immunostaining images showing F4/80+ macrophages in cryosectioned liver tissue (3 month, 17months(PBS), 17months(mAb) 17months(IgG), n=5). (B) F4/80 fluorescence in 50 randomized regions from 5 images of 5 independent liver sections for each genotype were quantified using image J (graphs show averaged values for the same region from 5 images).

Data information: Statistical significances were determined by Kruskal-Wallis test. Results shown are mean values ± SE.

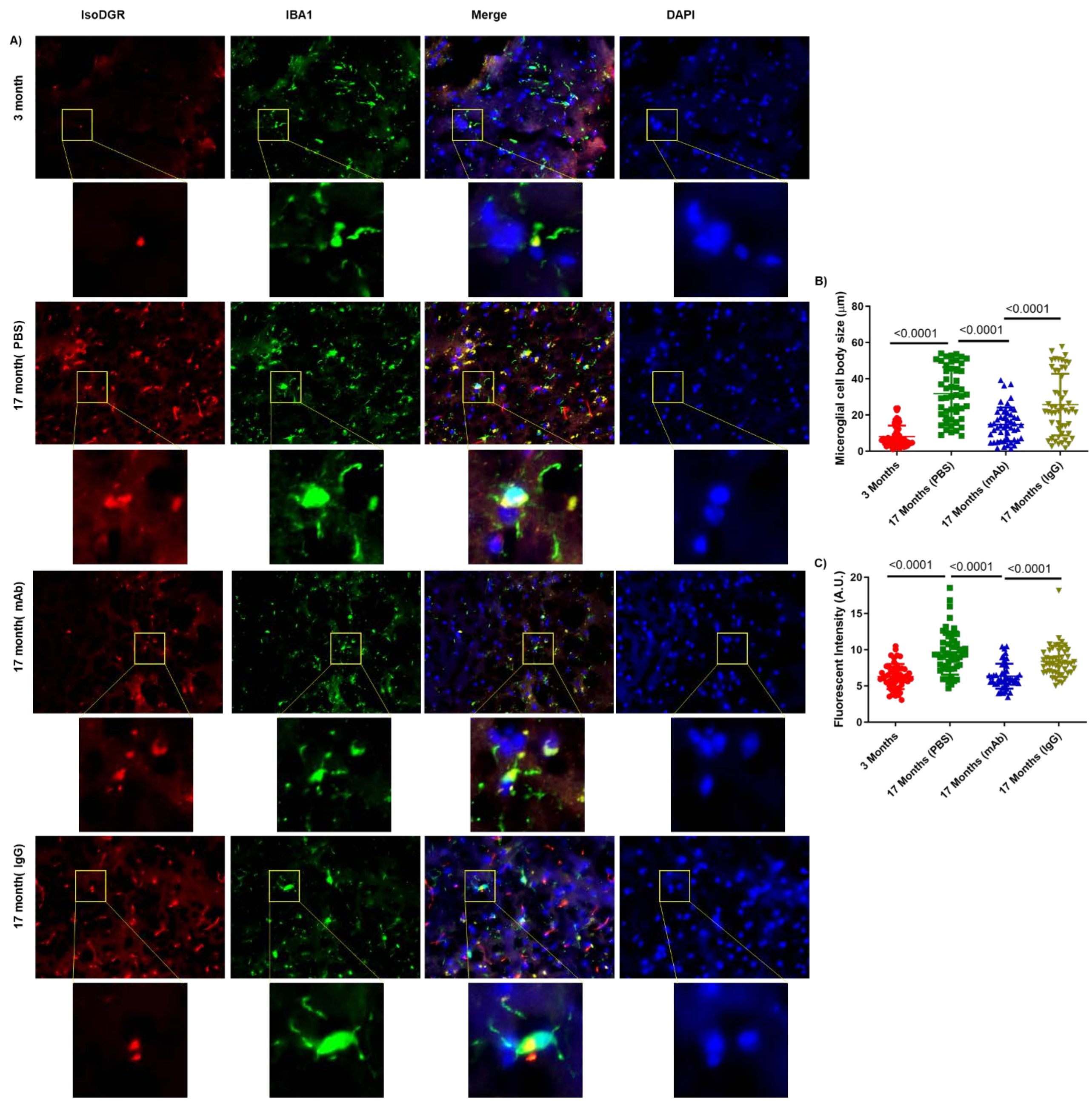

Appendix Figure S12: mAb treatment reduces the size of cellular body of microglia in aged WT mice brain.

(A) Representative immunostaining images showing isoDGR distribution and Iba1+ microglial cells in cryosectioned brain tissue (3 month, 17months (PBS), 17months (mAb) 17months(IgG), n=5). (B)The graph representing size of cellular body of microglia. (C)IsoDGR fluorescence in 50 randomized regions from 5 images of 5 independent brain sections for each genotype were quantified using image J (graphs show averaged values for the same region from 5 images).

Data information: Statistical significances were determined by Kruskal-Wallis test. Results shown are mean values ± SE.

Full WB images of Fig. 1A

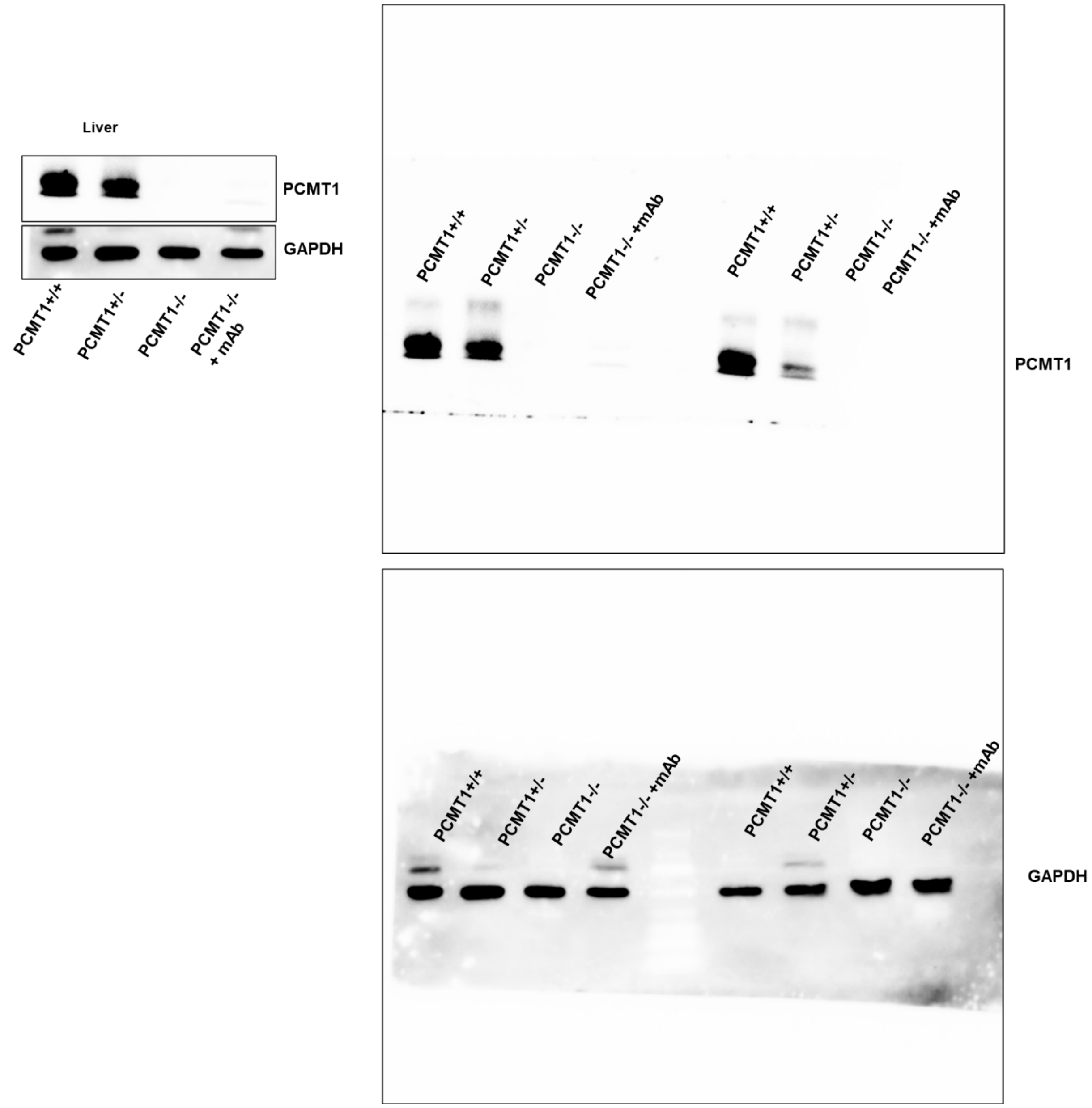

Appendix Figure S13: Full WB images for Figure 1A.

Table S1 Primers used in genotyping mouse Pcmt1 gene.

Primer Name	Genotyping Primer
oIMR108	CGG CTG CAT ACG CTT GAT C
oIMR1081	CGA CAA GAC CGG CTT CCA T
oIMR1544	CAC GTG GGC TCC AGC ATT
oIMR3580	TCA CCA GTC ATT TCT GCC TTT G

Table S2 Primers used for quantitative RT-PCR analysis of mouse genes, related to Figure 4A.

Gene Name	Forward Primer	Reverse primer
MCP1	TGATCCCAATGAGTAGGCTGGAG	ATGTCTGGACCCATTCCTTCTTG
TNF-α	5′-GCCTCTTCTCATTCCTGCTTG-3′	5′-CTGATGAGAGGGAGGCCATT-3
IL-23	CTGAGAAGCAGGCAACAAG	GTGAAGATGTCCGAGTCCAG
GAPDH	CAAGGTCATCCATGACAACTTTG	GTCCACCACCCTGTTGCTGTAG
