# Supplementary figures and images for: Immunotherapy targeting isoDGR‐protein damage extends lifespan in a mouse model of protein deamidation (part 2 of 2)
Source: EMBO Mol Med. 2023 Nov 16;15(12):e18526. doi: 10.15252/emmm.202318526 (PMC10701600; doi:10.15252/emmm.202318526)

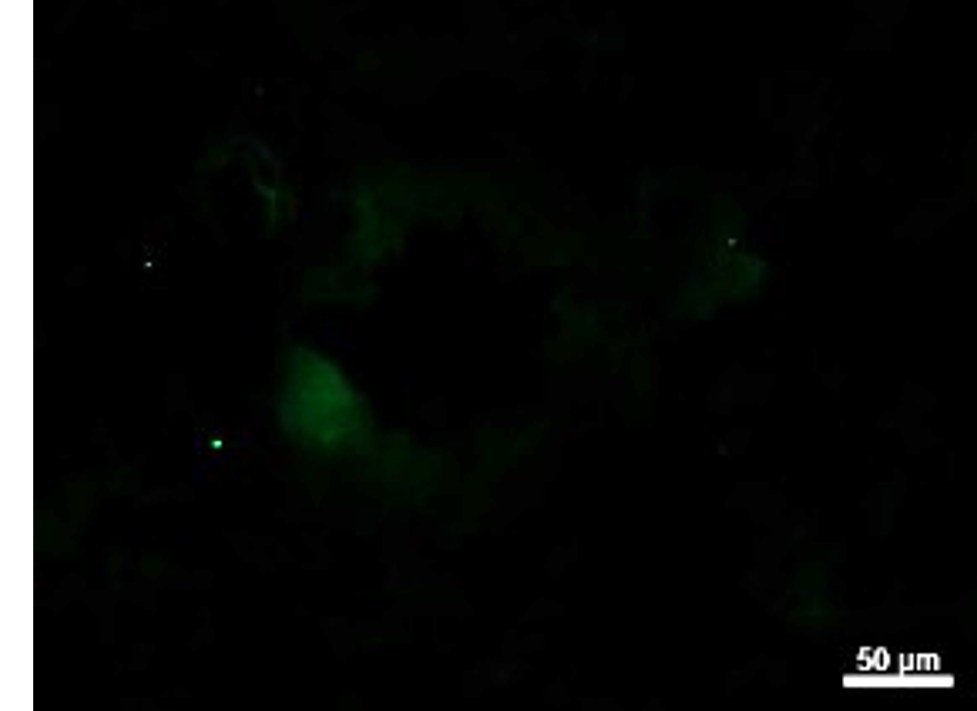

Supplement: Supplementary file 2 — Source Data for Appendix [file EMMM-15-e18526-s002.zip › Fig.S4/Fig.S4B/Fig.4B_WT_plasma/Fig.S4B_IsoDGR.tif]

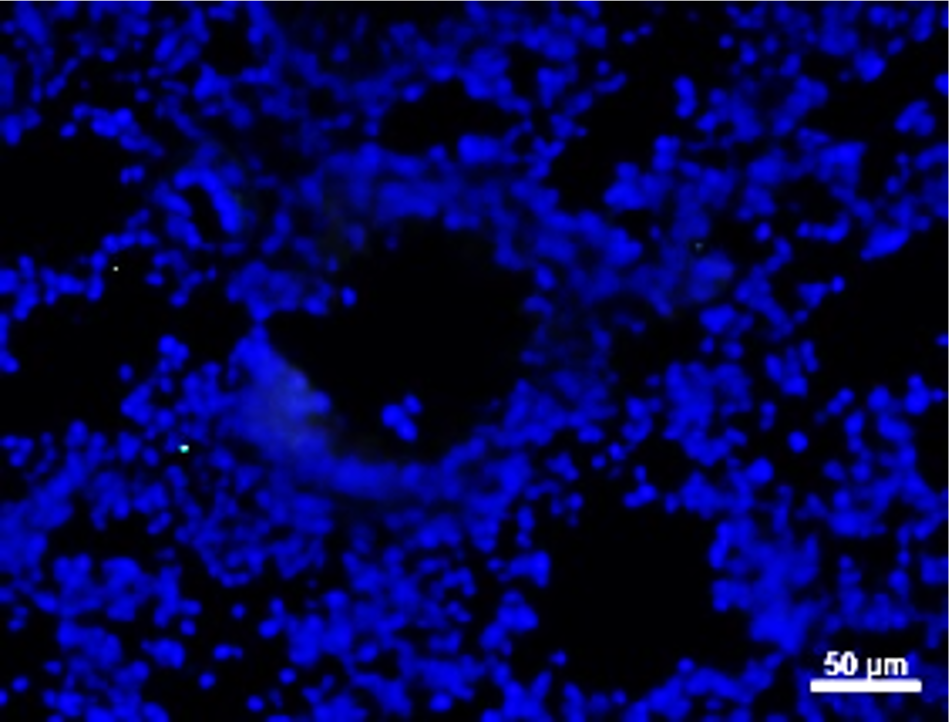

Supplement: Supplementary file 2 — Source Data for Appendix [file EMMM-15-e18526-s002.zip › Fig.S4/Fig.S4B/Fig.4B_WT_plasma/Fig.S4B_Merge.tif]

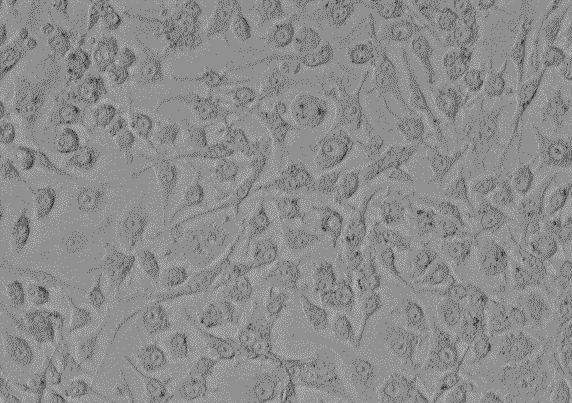

Supplement: Supplementary file 2 — Source Data for Appendix [file EMMM-15-e18526-s002.zip › Fig.S7/Fig.S7A/Blank/Fig.S7_Blank.tif]

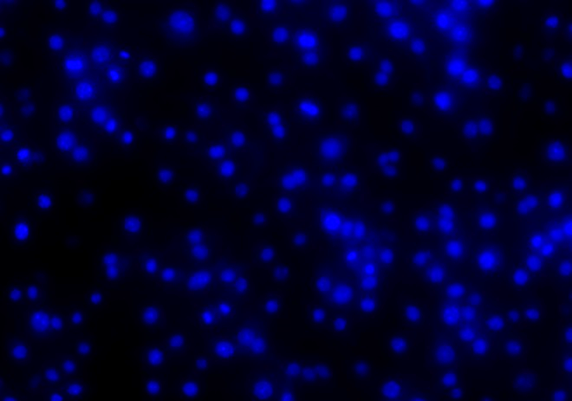

Supplement: Supplementary file 2 — Source Data for Appendix [file EMMM-15-e18526-s002.zip › Fig.S7/Fig.S7A/Blank/Fig.S7_DAPI.tif]

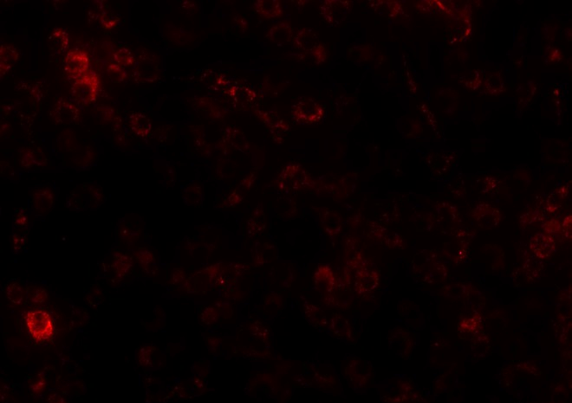

Supplement: Supplementary file 2 — Source Data for Appendix [file EMMM-15-e18526-s002.zip › Fig.S7/Fig.S7A/Blank/Fig.S7_FN-IsoDGR.tif]

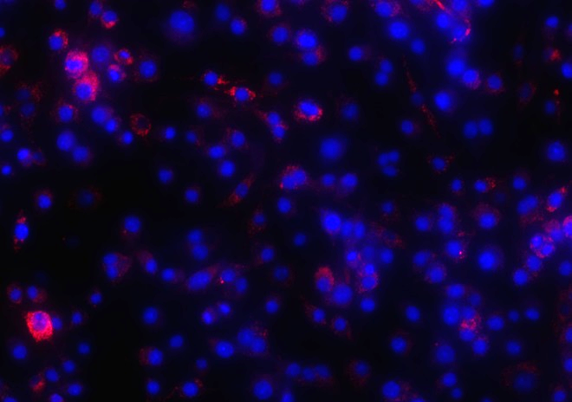

Supplement: Supplementary file 2 — Source Data for Appendix [file EMMM-15-e18526-s002.zip › Fig.S7/Fig.S7A/Blank/Fig.S7_Merge.tif]

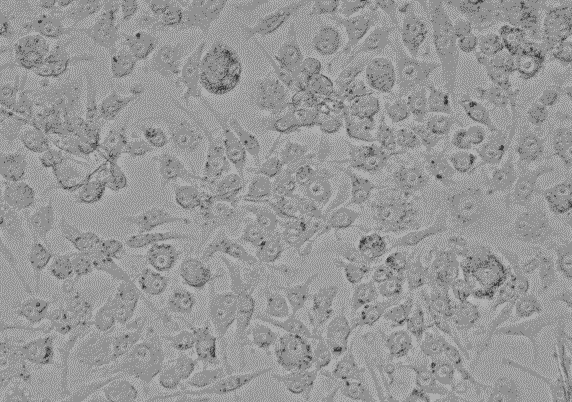

Supplement: Supplementary file 2 — Source Data for Appendix [file EMMM-15-e18526-s002.zip › Fig.S7/Fig.S7A/FN-IsoDGR/Fig.S7_Blank.tif]

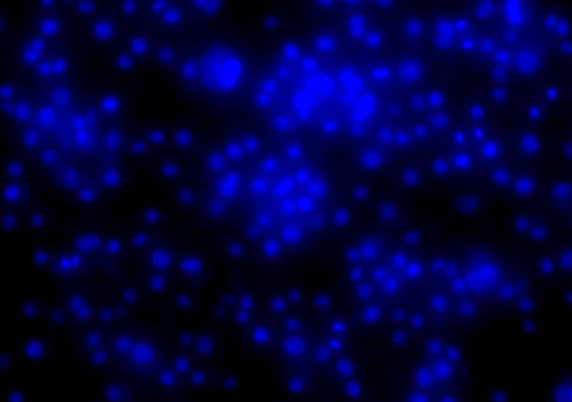

Supplement: Supplementary file 2 — Source Data for Appendix [file EMMM-15-e18526-s002.zip › Fig.S7/Fig.S7A/FN-IsoDGR/Fig.S7_DAPI.tif]

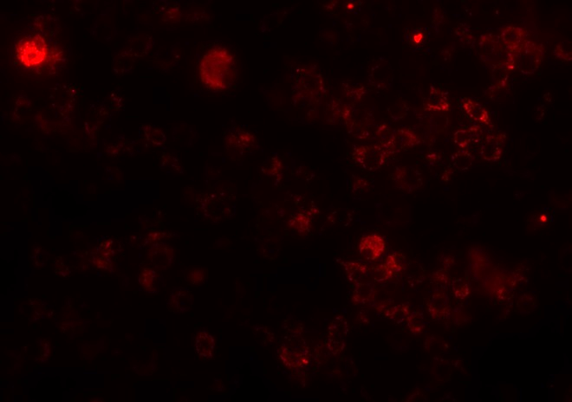

Supplement: Supplementary file 2 — Source Data for Appendix [file EMMM-15-e18526-s002.zip › Fig.S7/Fig.S7A/FN-IsoDGR/Fig.S7_FN_IsoDGR.tif]

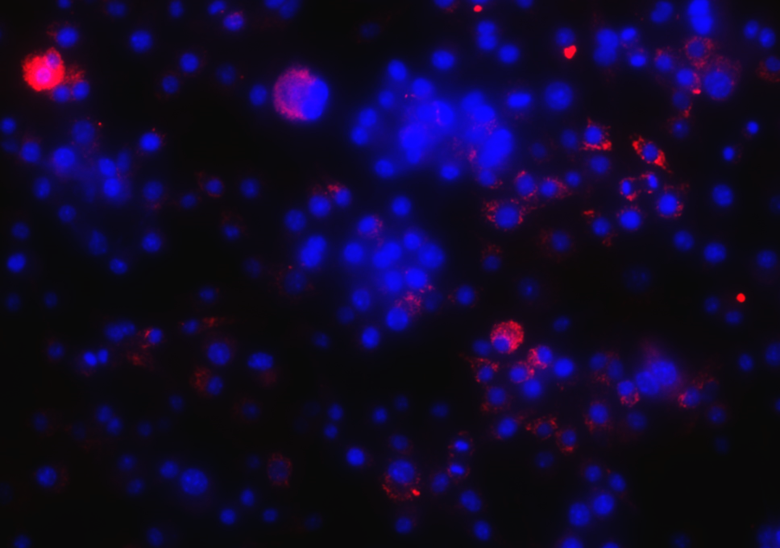

Supplement: Supplementary file 2 — Source Data for Appendix [file EMMM-15-e18526-s002.zip › Fig.S7/Fig.S7A/FN-IsoDGR/Fig.S7_Merge.tif]

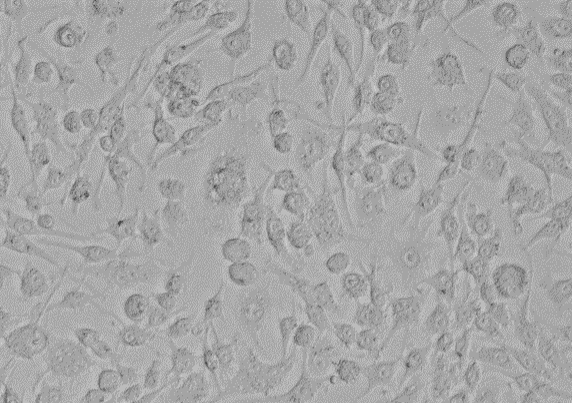

Supplement: Supplementary file 2 — Source Data for Appendix [file EMMM-15-e18526-s002.zip › Fig.S7/Fig.S7A/FN-isoDGR+1ug_mAb/Fig.S7_Blank.tif]

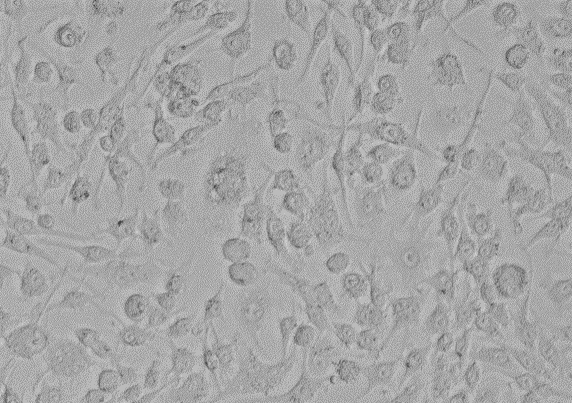

Supplement: Supplementary file 2 — Source Data for Appendix [file EMMM-15-e18526-s002.zip › Fig.S7/Fig.S7A/FN-isoDGR+1ug_mAb/Fig.S7_Blank.tif.png]

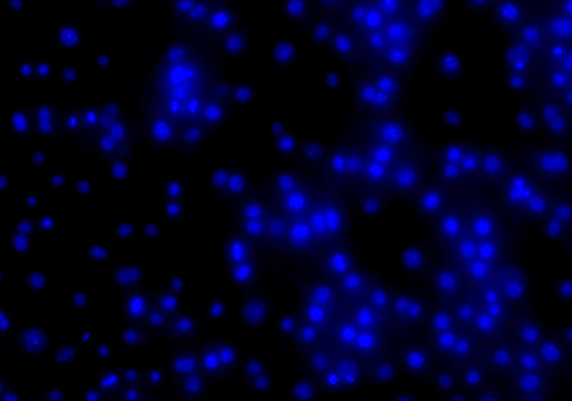

Supplement: Supplementary file 2 — Source Data for Appendix [file EMMM-15-e18526-s002.zip › Fig.S7/Fig.S7A/FN-isoDGR+1ug_mAb/Fig.S7_DAPI.tif]

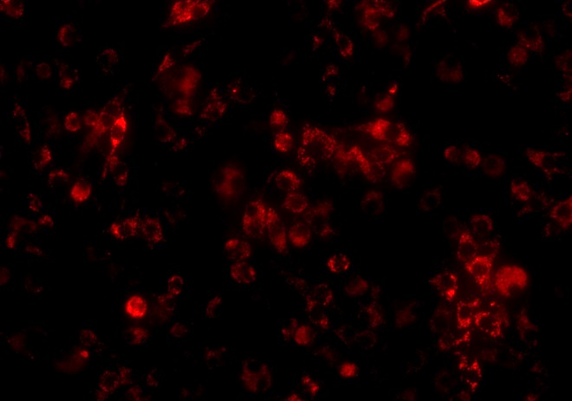

Supplement: Supplementary file 2 — Source Data for Appendix [file EMMM-15-e18526-s002.zip › Fig.S7/Fig.S7A/FN-isoDGR+1ug_mAb/Fig.S7_FNIsoDGR.tif]

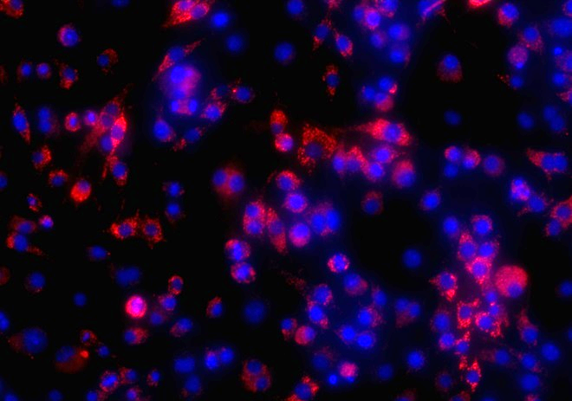

Supplement: Supplementary file 2 — Source Data for Appendix [file EMMM-15-e18526-s002.zip › Fig.S7/Fig.S7A/FN-isoDGR+1ug_mAb/Fig.S7_Merge.tif]

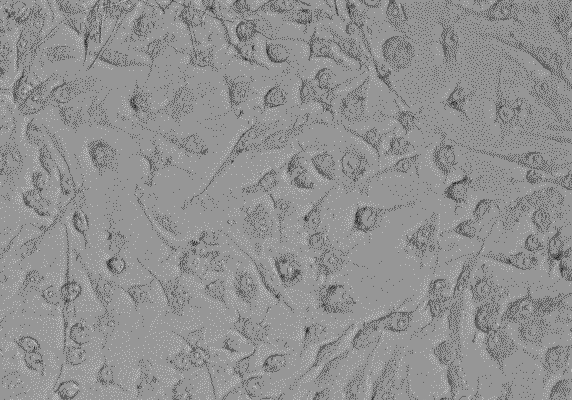

Supplement: Supplementary file 2 — Source Data for Appendix [file EMMM-15-e18526-s002.zip › Fig.S7/Fig.S7A/FN-IsoDGR+2ug_mAb/Fig.S7_Blank.tif]

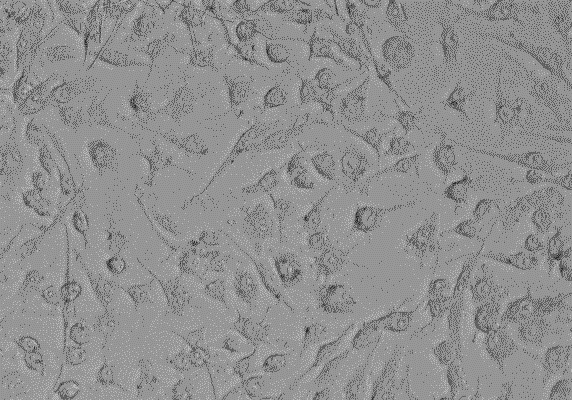

Supplement: Supplementary file 2 — Source Data for Appendix [file EMMM-15-e18526-s002.zip › Fig.S7/Fig.S7A/FN-IsoDGR+2ug_mAb/Fig.S7_Blank.tif.png]

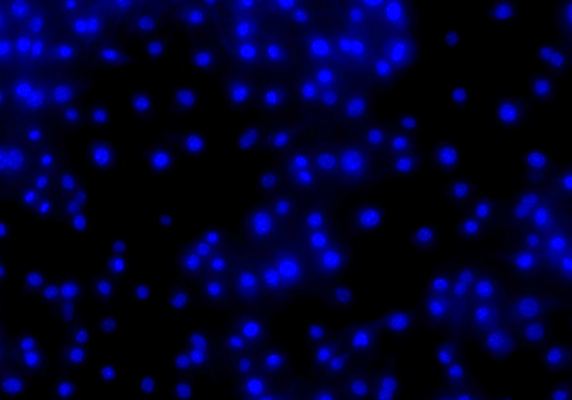

Supplement: Supplementary file 2 — Source Data for Appendix [file EMMM-15-e18526-s002.zip › Fig.S7/Fig.S7A/FN-IsoDGR+2ug_mAb/Fig.S7_DAPI.tif]

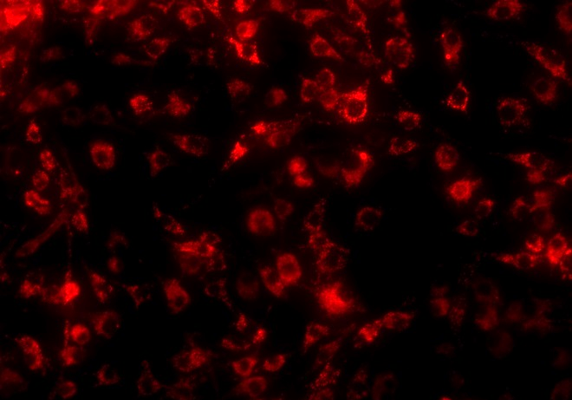

Supplement: Supplementary file 2 — Source Data for Appendix [file EMMM-15-e18526-s002.zip › Fig.S7/Fig.S7A/FN-IsoDGR+2ug_mAb/Fig.S7_FN_IsoDGR.tif]

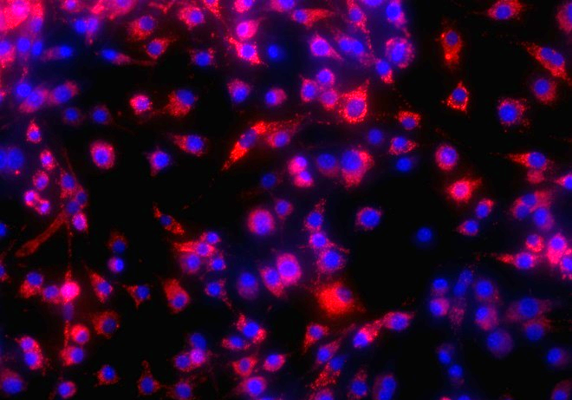

Supplement: Supplementary file 2 — Source Data for Appendix [file EMMM-15-e18526-s002.zip › Fig.S7/Fig.S7A/FN-IsoDGR+2ug_mAb/Fig.S7_Merge.tif]

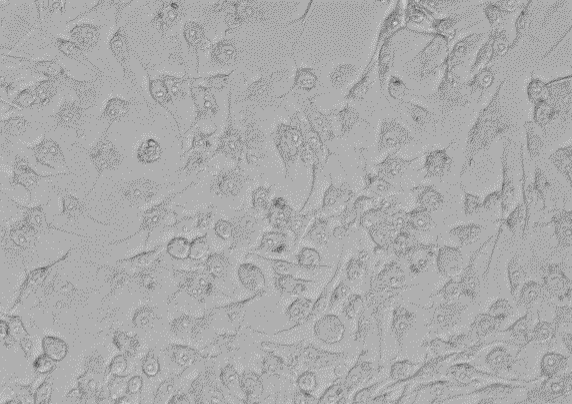

Supplement: Supplementary file 2 — Source Data for Appendix [file EMMM-15-e18526-s002.zip › Fig.S7/Fig.S7A/FN-IsoDGR+_2ug_Igg/Fig.S7_Blank.tif]

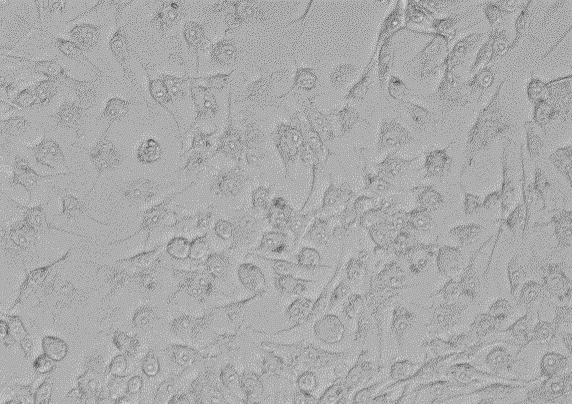

Supplement: Supplementary file 2 — Source Data for Appendix [file EMMM-15-e18526-s002.zip › Fig.S7/Fig.S7A/FN-IsoDGR+_2ug_Igg/Fig.S7_Blank.tif.png]

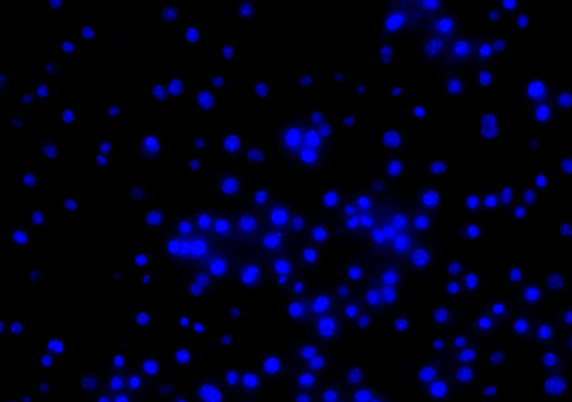

Supplement: Supplementary file 2 — Source Data for Appendix [file EMMM-15-e18526-s002.zip › Fig.S7/Fig.S7A/FN-IsoDGR+_2ug_Igg/Fig.S7_DAPI.tif]

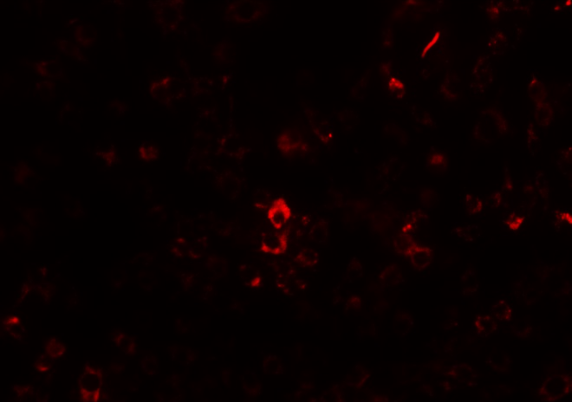

Supplement: Supplementary file 2 — Source Data for Appendix [file EMMM-15-e18526-s002.zip › Fig.S7/Fig.S7A/FN-IsoDGR+_2ug_Igg/Fig.S7_FNIsoDGR.tif]

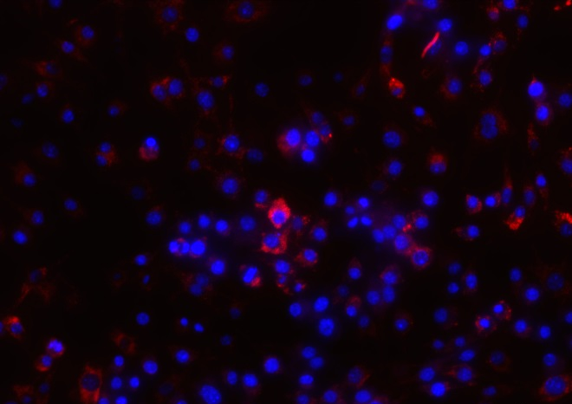

Supplement: Supplementary file 2 — Source Data for Appendix [file EMMM-15-e18526-s002.zip › Fig.S7/Fig.S7A/FN-IsoDGR+_2ug_Igg/Fig.S7_Merge.tif]

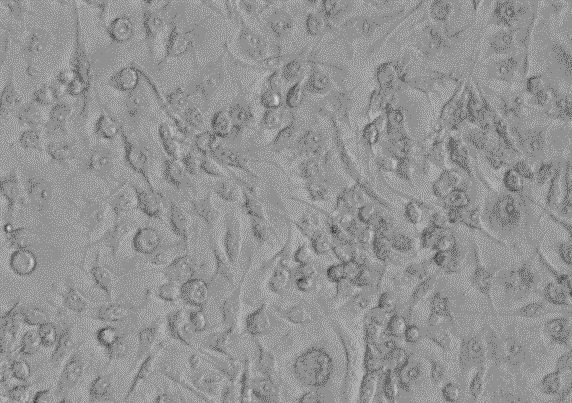

Supplement: Supplementary file 2 — Source Data for Appendix [file EMMM-15-e18526-s002.zip › Fig.S7/Fig.S7A/FN_IsoDGR+5ug_mAb/Fig.S7_Blank.tif]

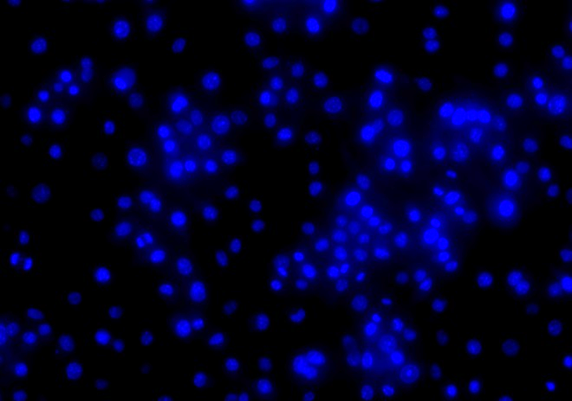

Supplement: Supplementary file 2 — Source Data for Appendix [file EMMM-15-e18526-s002.zip › Fig.S7/Fig.S7A/FN_IsoDGR+5ug_mAb/Fig.S7_DAPI.tif]

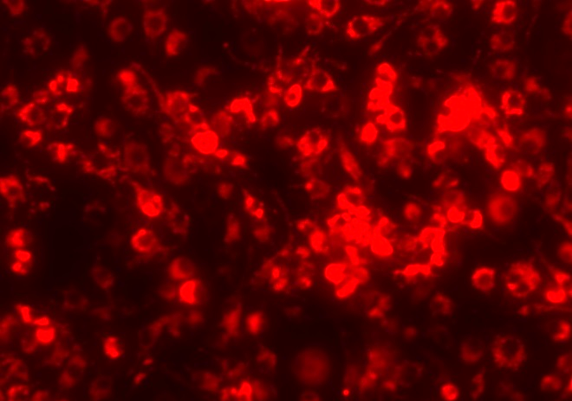

Supplement: Supplementary file 2 — Source Data for Appendix [file EMMM-15-e18526-s002.zip › Fig.S7/Fig.S7A/FN_IsoDGR+5ug_mAb/Fig.S7_FN_IsoDGR.tif]

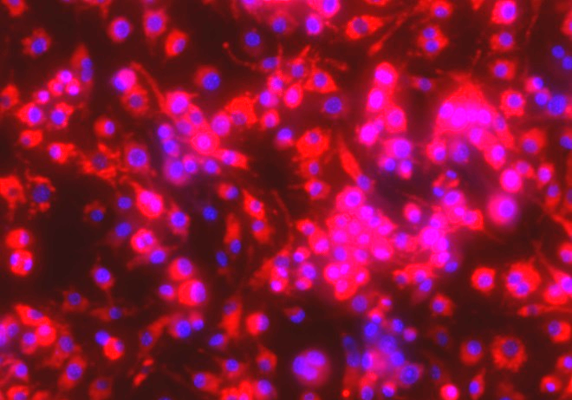

Supplement: Supplementary file 2 — Source Data for Appendix [file EMMM-15-e18526-s002.zip › Fig.S7/Fig.S7A/FN_IsoDGR+5ug_mAb/Fig.S7_Merge.tif]

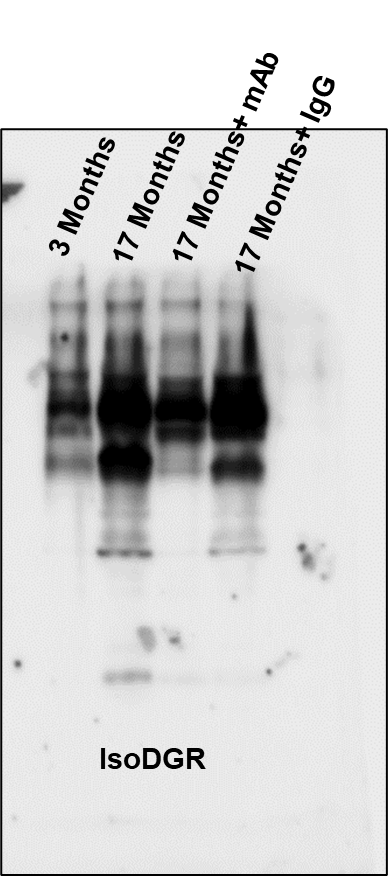

Supplement: Supplementary file 2 — Source Data for Appendix [file EMMM-15-e18526-s002.zip › Fig.S9/Fig.S9_IsoDGR.tif]

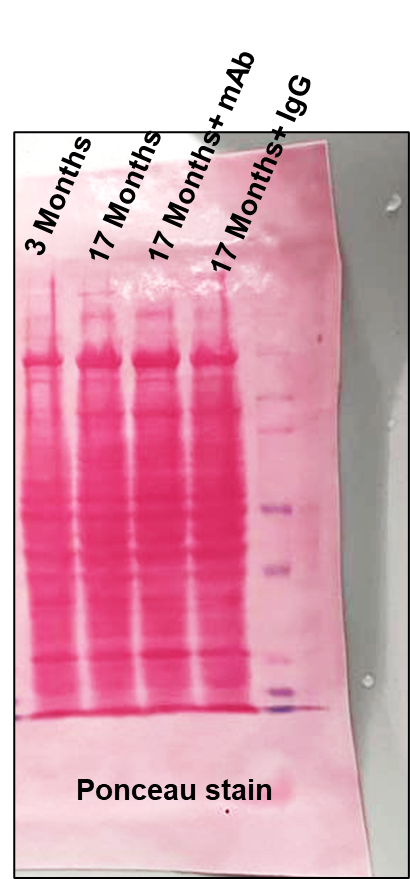

Supplement: Supplementary file 2 — Source Data for Appendix [file EMMM-15-e18526-s002.zip › Fig.S9/Fig.S9_Ponceau_Stain.tif]

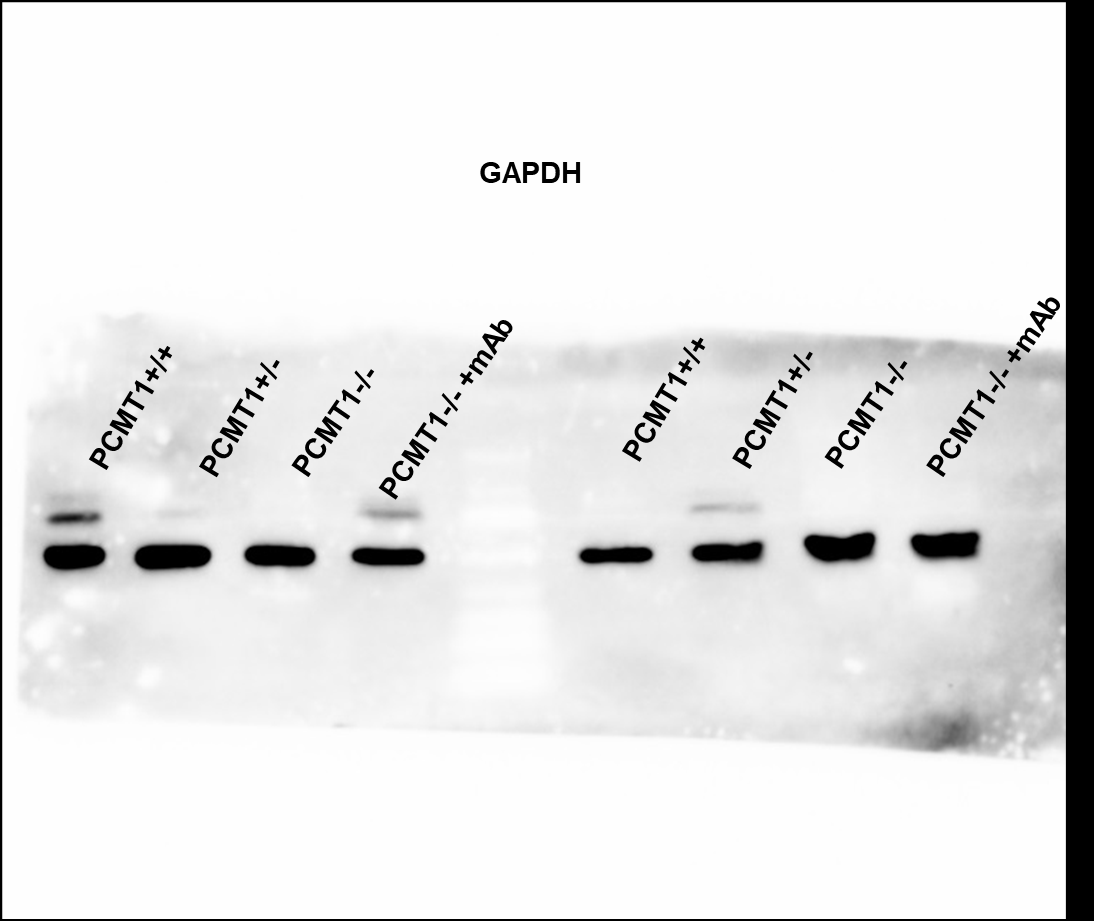

Supplement: Supplementary file 3 — Source Data for Figure 1 [file EMMM-15-e18526-s004.zip › Fig.1/Fig.1A/Fig.1A_GAPDH.tif]

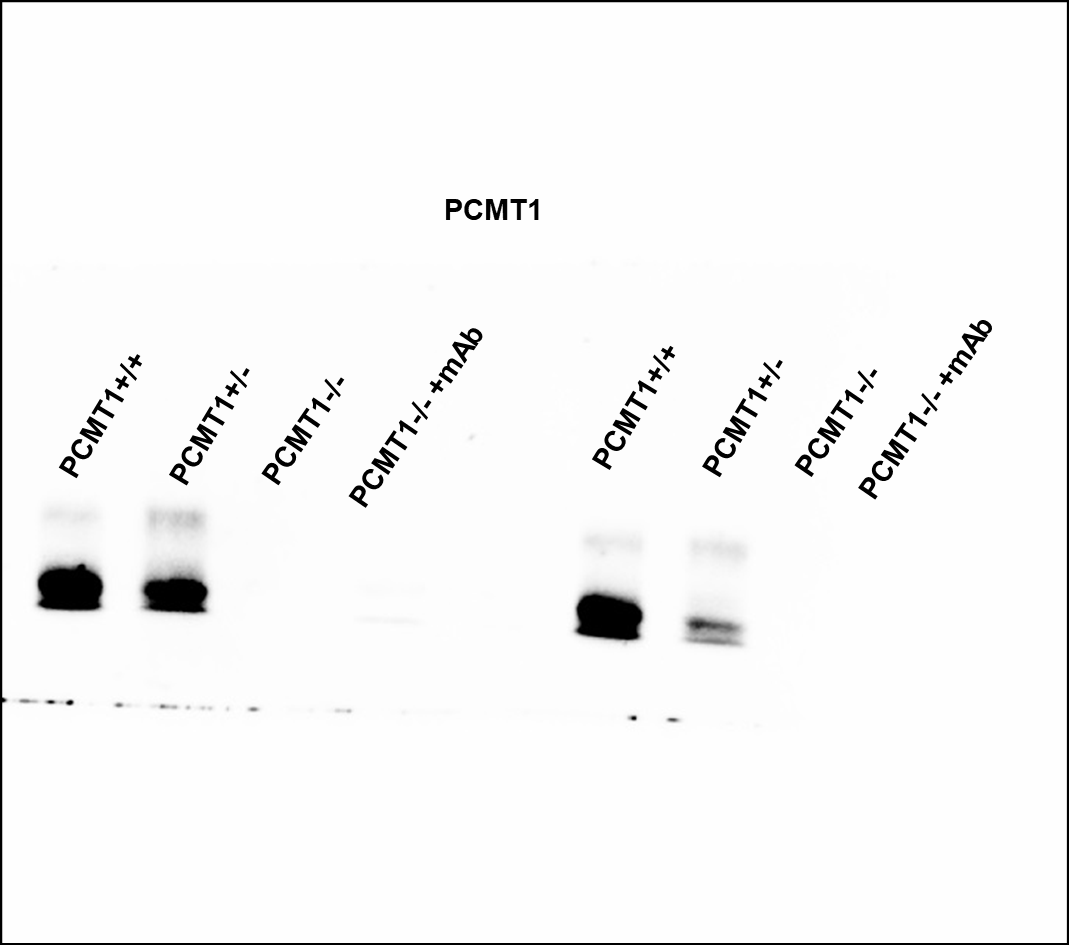

Supplement: Supplementary file 3 — Source Data for Figure 1 [file EMMM-15-e18526-s004.zip › Fig.1/Fig.1A/Fig.1A_PCMT1.tif]

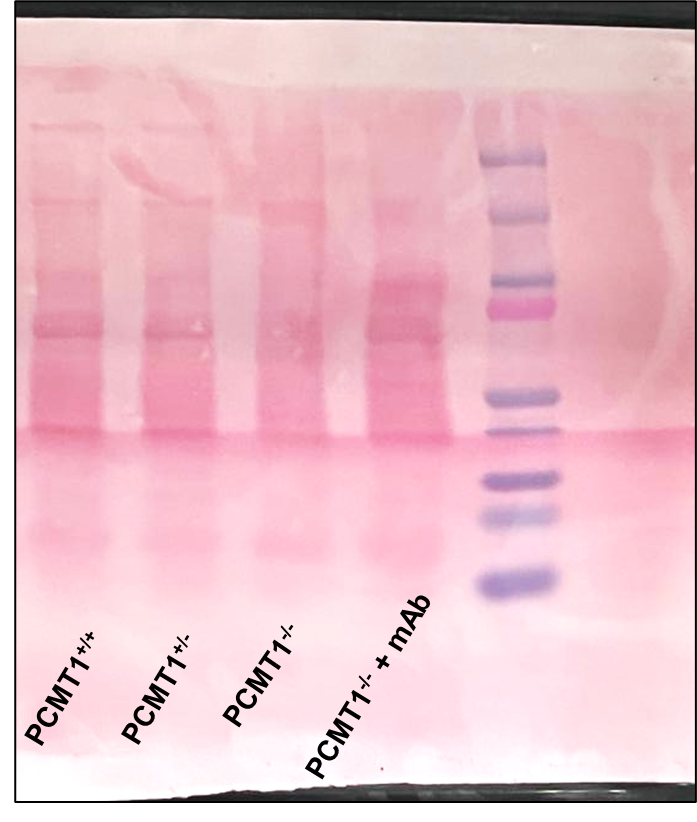

Supplement: Supplementary file 4 — Source Data for Figure 2 [file EMMM-15-e18526-s006.zip › Fig.2/Fig.2A/Fig.2A..tif]

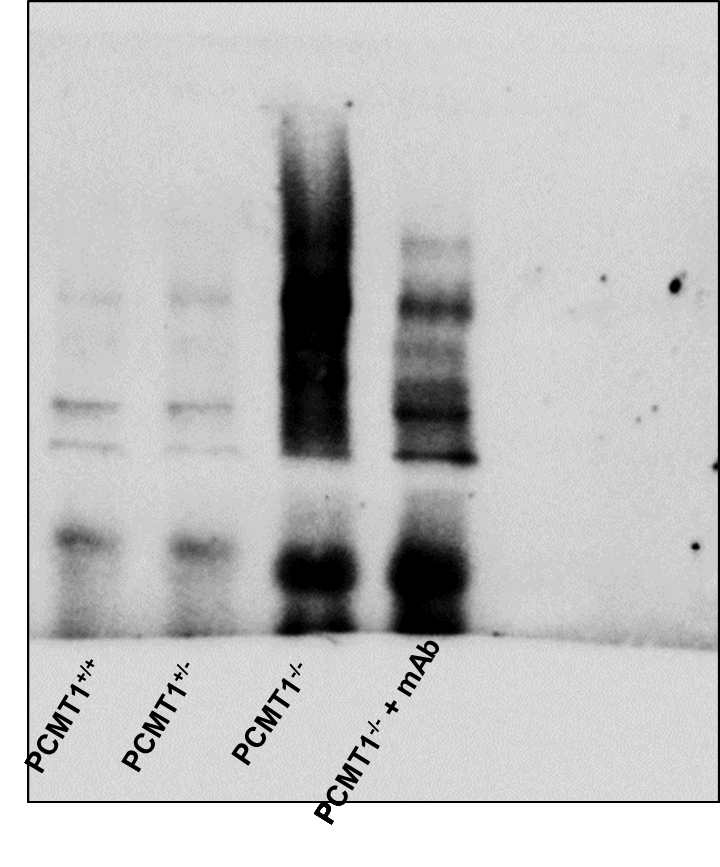

Supplement: Supplementary file 4 — Source Data for Figure 2 [file EMMM-15-e18526-s006.zip › Fig.2/Fig.2A/Fig.2A.tif]

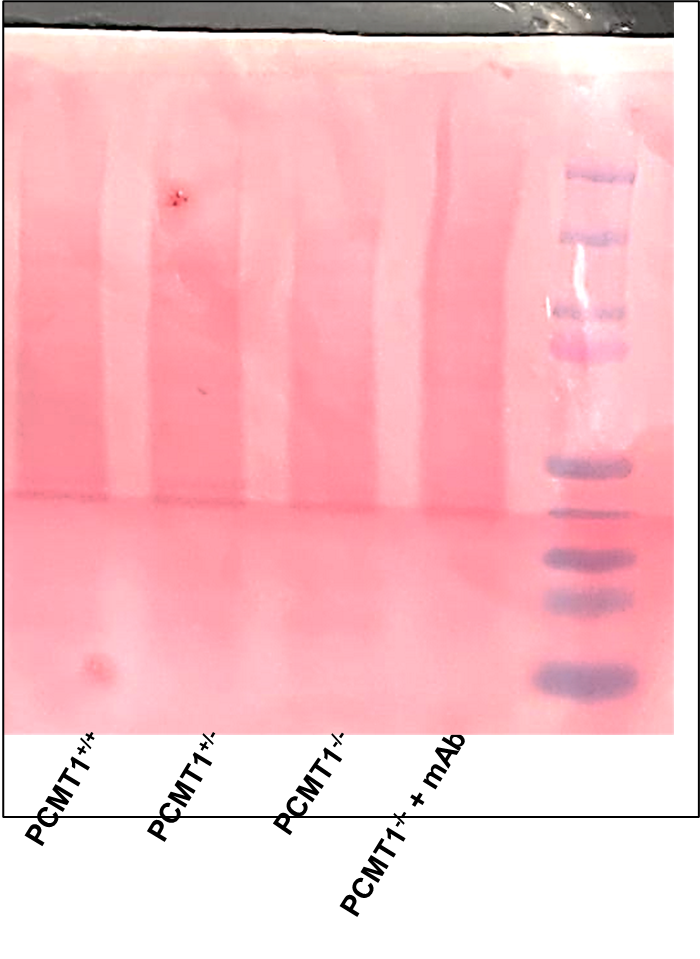

Supplement: Supplementary file 4 — Source Data for Figure 2 [file EMMM-15-e18526-s006.zip › Fig.2/Fig.2B/Fig.2B..tif]

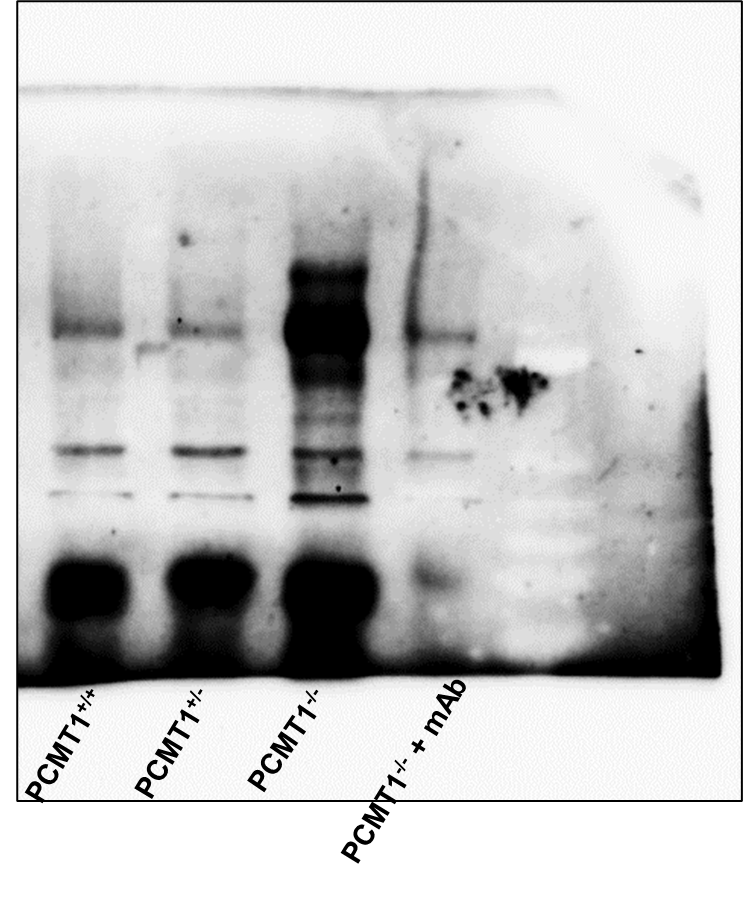

Supplement: Supplementary file 4 — Source Data for Figure 2 [file EMMM-15-e18526-s006.zip › Fig.2/Fig.2B/Fig.2B.tif]

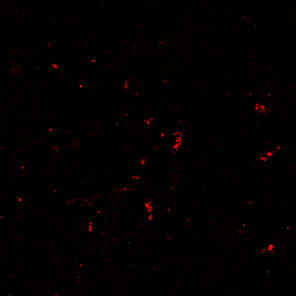

Supplement: Supplementary file 5 — Source Data for Figure 3 [file EMMM-15-e18526-s005.zip › Fig.3/Fig.3A/PCMT1++/Fig3A.CD68.tif]

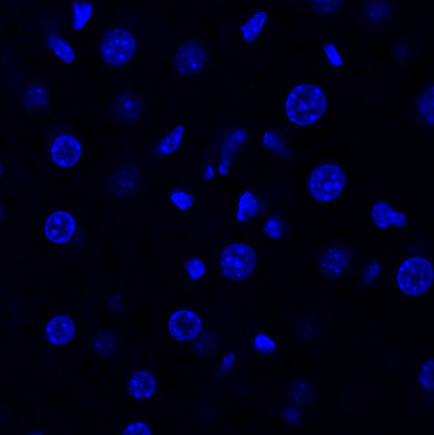

Supplement: Supplementary file 5 — Source Data for Figure 3 [file EMMM-15-e18526-s005.zip › Fig.3/Fig.3A/PCMT1++/Fig3A.DAPI.tif]

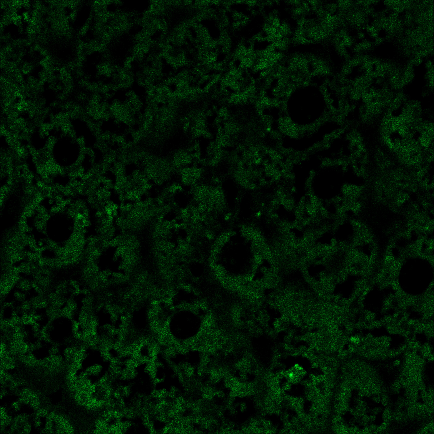

Supplement: Supplementary file 5 — Source Data for Figure 3 [file EMMM-15-e18526-s005.zip › Fig.3/Fig.3A/PCMT1++/Fig3A.IsoDGR.tif]

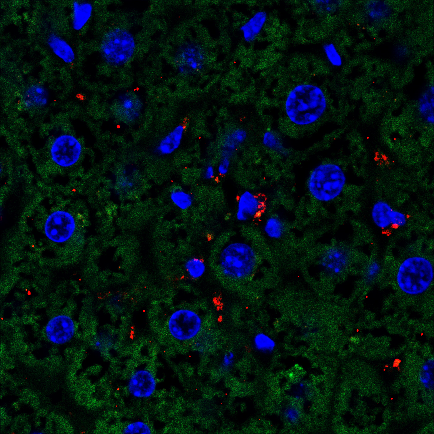

Supplement: Supplementary file 5 — Source Data for Figure 3 [file EMMM-15-e18526-s005.zip › Fig.3/Fig.3A/PCMT1++/Fig3A.Merge.tif]

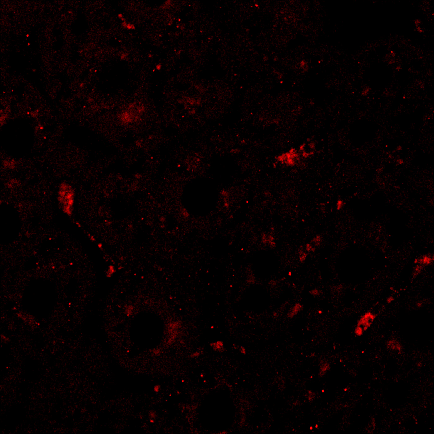

Supplement: Supplementary file 5 — Source Data for Figure 3 [file EMMM-15-e18526-s005.zip › Fig.3/Fig.3A/PCMT1+-/Fig.3A_CD68.tif]

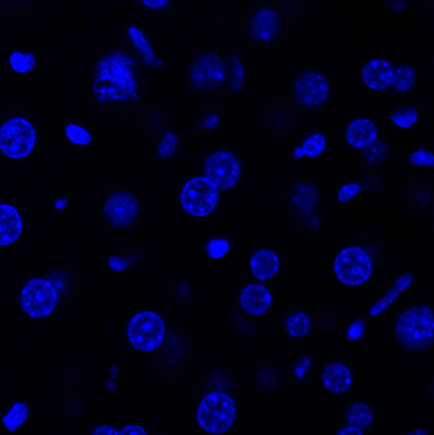

Supplement: Supplementary file 5 — Source Data for Figure 3 [file EMMM-15-e18526-s005.zip › Fig.3/Fig.3A/PCMT1+-/Fig.3A_DAPI.tif]

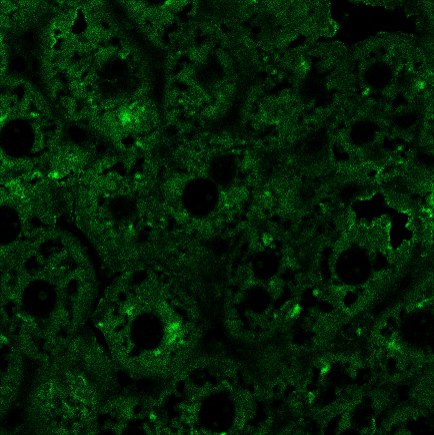

Supplement: Supplementary file 5 — Source Data for Figure 3 [file EMMM-15-e18526-s005.zip › Fig.3/Fig.3A/PCMT1+-/Fig.3A_IsoDGR.tif]

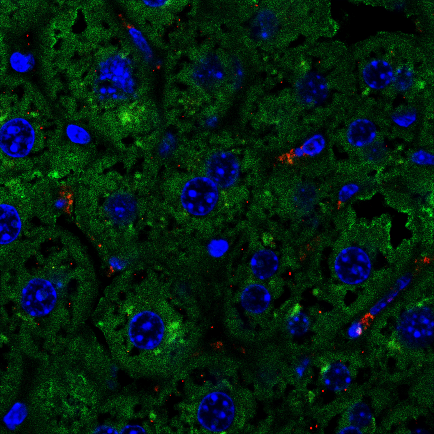

Supplement: Supplementary file 5 — Source Data for Figure 3 [file EMMM-15-e18526-s005.zip › Fig.3/Fig.3A/PCMT1+-/Fig.3A_Merge.tif]

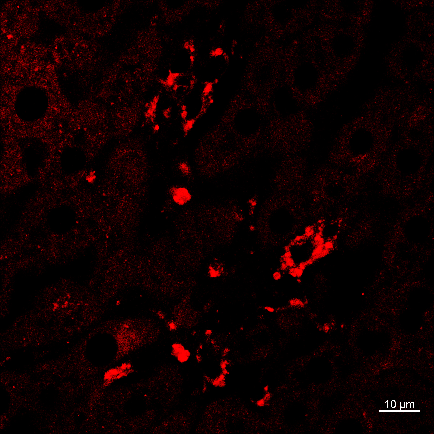

Supplement: Supplementary file 5 — Source Data for Figure 3 [file EMMM-15-e18526-s005.zip › Fig.3/Fig.3A/PCMT1--/Fig.3A_CD68.tif]

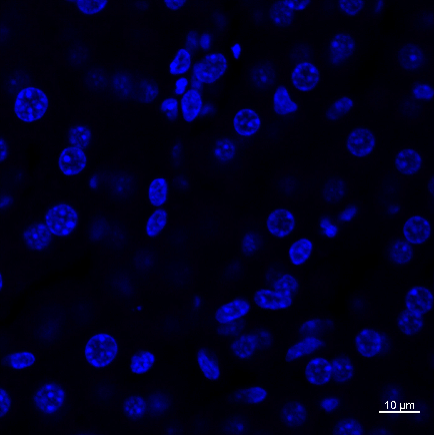

Supplement: Supplementary file 5 — Source Data for Figure 3 [file EMMM-15-e18526-s005.zip › Fig.3/Fig.3A/PCMT1--/Fig.3A_DAPI.tif]

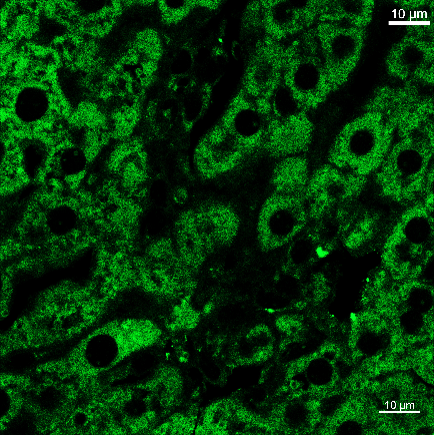

Supplement: Supplementary file 5 — Source Data for Figure 3 [file EMMM-15-e18526-s005.zip › Fig.3/Fig.3A/PCMT1--/Fig.3A_IsoDGR.tif]

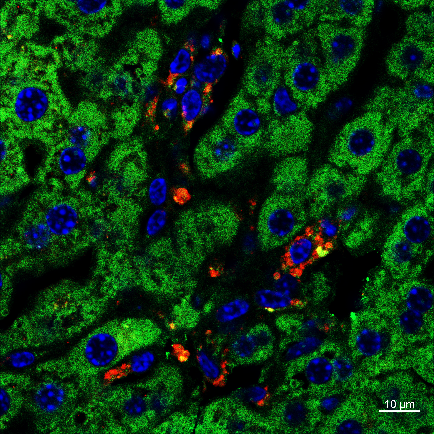

Supplement: Supplementary file 5 — Source Data for Figure 3 [file EMMM-15-e18526-s005.zip › Fig.3/Fig.3A/PCMT1--/Fig.3A_Merge.tif]

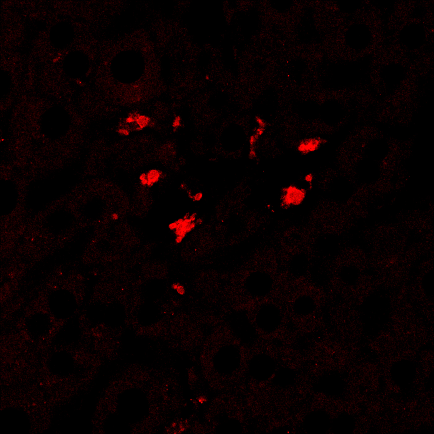

Supplement: Supplementary file 5 — Source Data for Figure 3 [file EMMM-15-e18526-s005.zip › Fig.3/Fig.3A/PCMT1--+mAb/Fig.1A_CD68.tif]

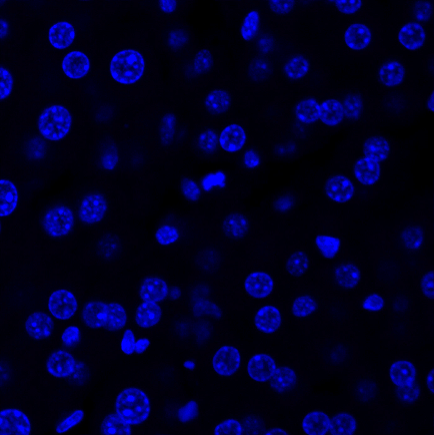

Supplement: Supplementary file 5 — Source Data for Figure 3 [file EMMM-15-e18526-s005.zip › Fig.3/Fig.3A/PCMT1--+mAb/Fig.1A_DAPI.tif]

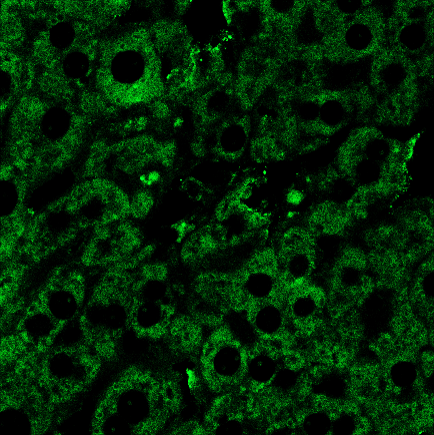

Supplement: Supplementary file 5 — Source Data for Figure 3 [file EMMM-15-e18526-s005.zip › Fig.3/Fig.3A/PCMT1--+mAb/Fig.1A_IsoDGR.tif]

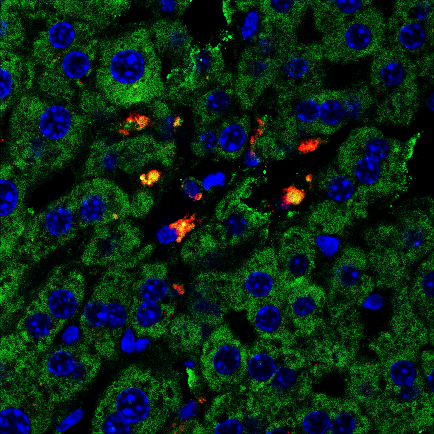

Supplement: Supplementary file 5 — Source Data for Figure 3 [file EMMM-15-e18526-s005.zip › Fig.3/Fig.3A/PCMT1--+mAb/Fig.1A_Merge.tif]

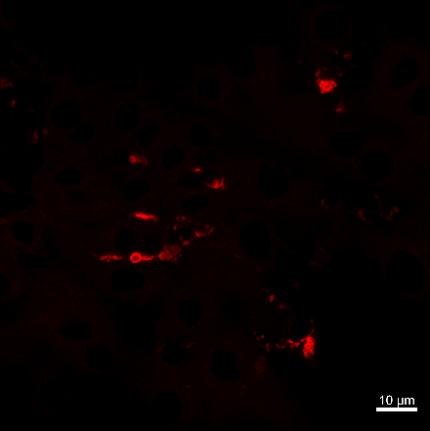

Supplement: Supplementary file 7 — Source Data for Figure 5 [file EMMM-15-e18526-s003.zip › Fig.5/Fig.5A/15months/PCMT1++/Fig.5A_CD68.tif]

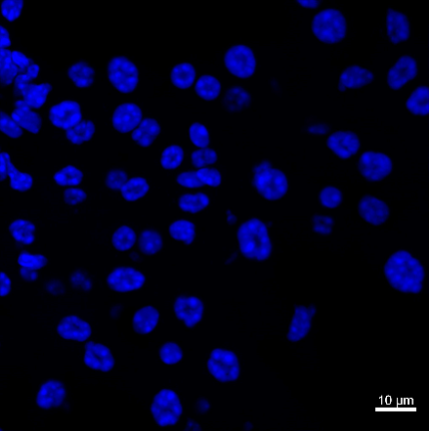

Supplement: Supplementary file 7 — Source Data for Figure 5 [file EMMM-15-e18526-s003.zip › Fig.5/Fig.5A/15months/PCMT1++/Fig.5A_DAPI.tif]

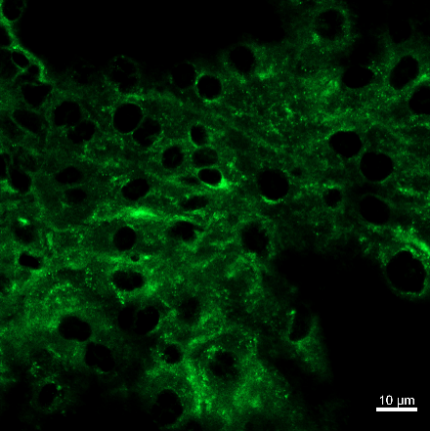

Supplement: Supplementary file 7 — Source Data for Figure 5 [file EMMM-15-e18526-s003.zip › Fig.5/Fig.5A/15months/PCMT1++/Fig.5A_IsoDGR.tif]

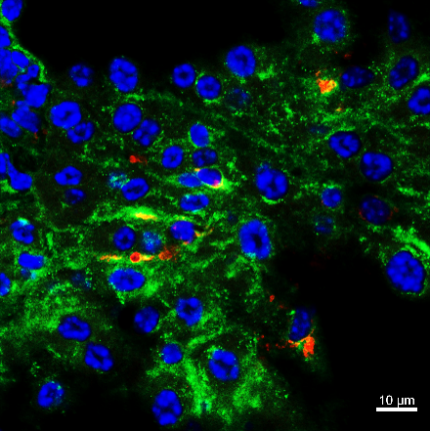

Supplement: Supplementary file 7 — Source Data for Figure 5 [file EMMM-15-e18526-s003.zip › Fig.5/Fig.5A/15months/PCMT1++/Fig.5A_Merge.tif]

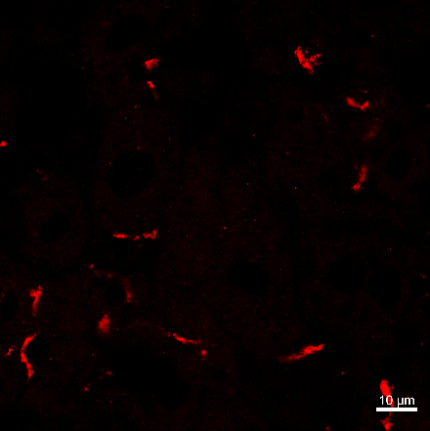

Supplement: Supplementary file 7 — Source Data for Figure 5 [file EMMM-15-e18526-s003.zip › Fig.5/Fig.5A/15months/PCMT1+-/Fig.5A_CD68.tif]

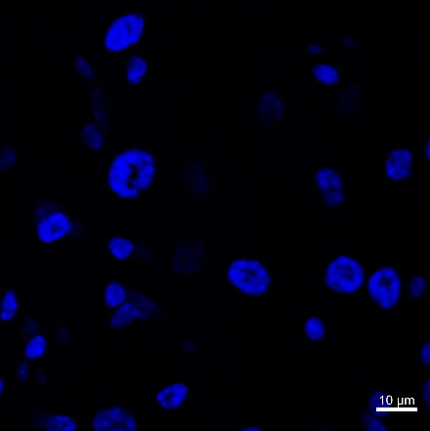

Supplement: Supplementary file 7 — Source Data for Figure 5 [file EMMM-15-e18526-s003.zip › Fig.5/Fig.5A/15months/PCMT1+-/Fig.5A_DAPI.tif]

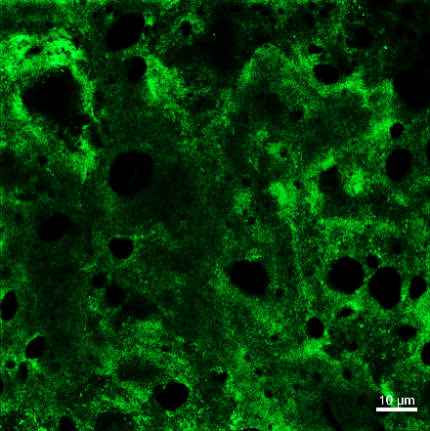

Supplement: Supplementary file 7 — Source Data for Figure 5 [file EMMM-15-e18526-s003.zip › Fig.5/Fig.5A/15months/PCMT1+-/Fig.5A_IsoDGR.tif]

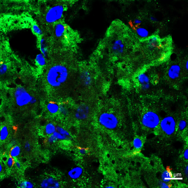

Supplement: Supplementary file 7 — Source Data for Figure 5 [file EMMM-15-e18526-s003.zip › Fig.5/Fig.5A/15months/PCMT1+-/Fig.5A_Merge.tif]

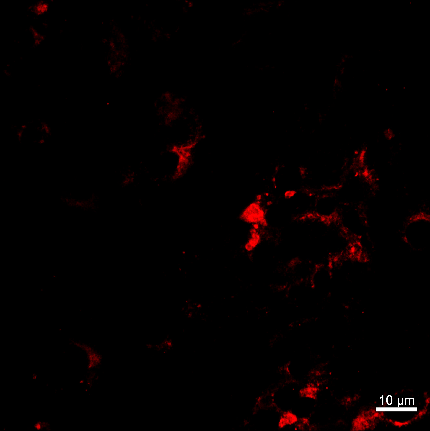

Supplement: Supplementary file 7 — Source Data for Figure 5 [file EMMM-15-e18526-s003.zip › Fig.5/Fig.5A/24Months/PCMT1++/Fig.5A_CD68.tif]

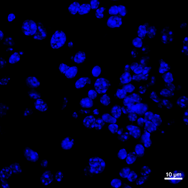

Supplement: Supplementary file 7 — Source Data for Figure 5 [file EMMM-15-e18526-s003.zip › Fig.5/Fig.5A/24Months/PCMT1++/Fig.5A_DAPI.tif]

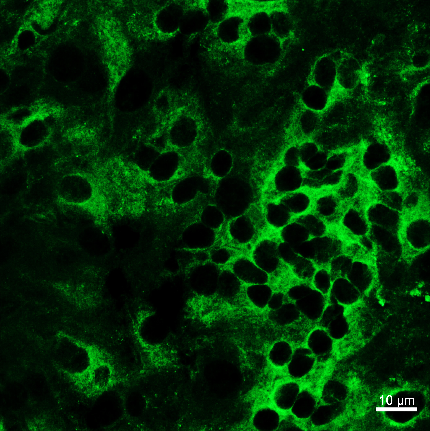

Supplement: Supplementary file 7 — Source Data for Figure 5 [file EMMM-15-e18526-s003.zip › Fig.5/Fig.5A/24Months/PCMT1++/Fig.5A_IsoDGR.tif]

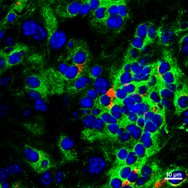

Supplement: Supplementary file 7 — Source Data for Figure 5 [file EMMM-15-e18526-s003.zip › Fig.5/Fig.5A/24Months/PCMT1++/Fig.5A_Merge.tif]

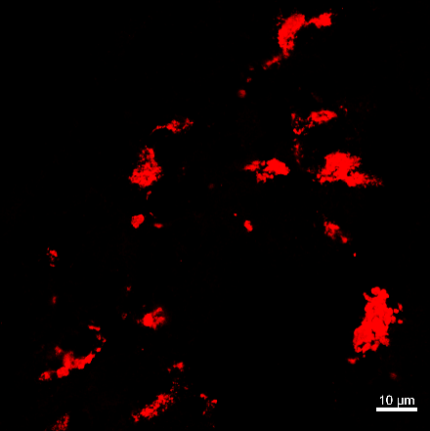

Supplement: Supplementary file 7 — Source Data for Figure 5 [file EMMM-15-e18526-s003.zip › Fig.5/Fig.5A/24Months/PCMT1+-/Fig.5A_CD68.tif]

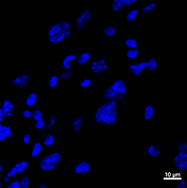

Supplement: Supplementary file 7 — Source Data for Figure 5 [file EMMM-15-e18526-s003.zip › Fig.5/Fig.5A/24Months/PCMT1+-/Fig.5A_DAPI.tif]

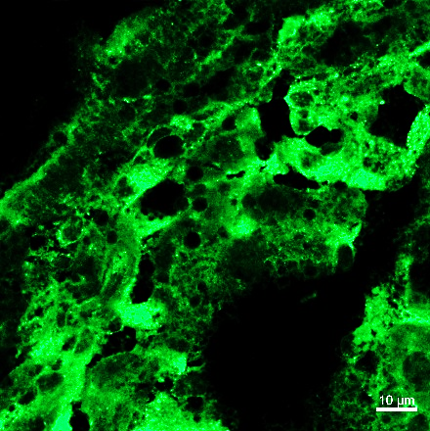

Supplement: Supplementary file 7 — Source Data for Figure 5 [file EMMM-15-e18526-s003.zip › Fig.5/Fig.5A/24Months/PCMT1+-/Fig.5A_IsoDGR.tif]

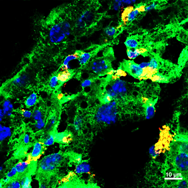

Supplement: Supplementary file 7 — Source Data for Figure 5 [file EMMM-15-e18526-s003.zip › Fig.5/Fig.5A/24Months/PCMT1+-/Fig.5A_Merge.tif]

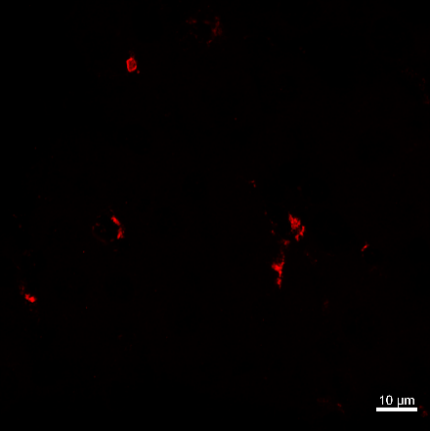

Supplement: Supplementary file 7 — Source Data for Figure 5 [file EMMM-15-e18526-s003.zip › Fig.5/Fig.5A/4months/PCMT1++/Fig.5A_CD68.tif]

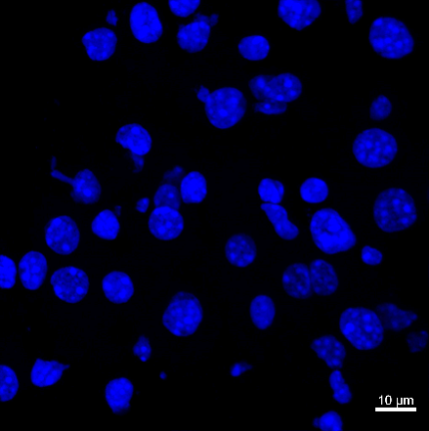

Supplement: Supplementary file 7 — Source Data for Figure 5 [file EMMM-15-e18526-s003.zip › Fig.5/Fig.5A/4months/PCMT1++/Fig.5A_DAPI.tif]

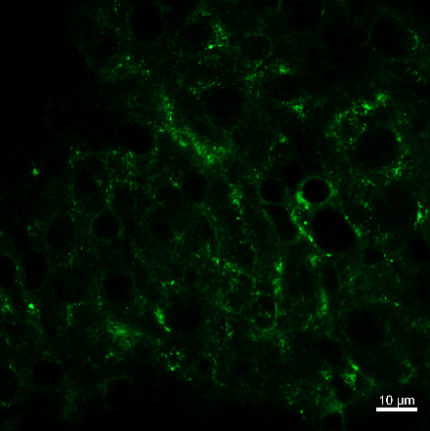

Supplement: Supplementary file 7 — Source Data for Figure 5 [file EMMM-15-e18526-s003.zip › Fig.5/Fig.5A/4months/PCMT1++/Fig.5A_IsoDGR.tif]

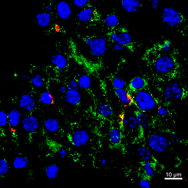

Supplement: Supplementary file 7 — Source Data for Figure 5 [file EMMM-15-e18526-s003.zip › Fig.5/Fig.5A/4months/PCMT1++/Fig.5A_Merge.tif]

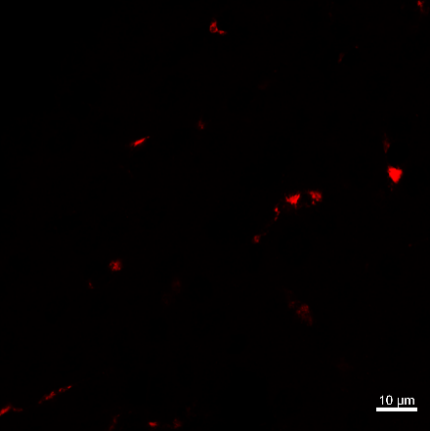

Supplement: Supplementary file 7 — Source Data for Figure 5 [file EMMM-15-e18526-s003.zip › Fig.5/Fig.5A/4months/PCMT1+-/Fig.1A_CD68.tif]

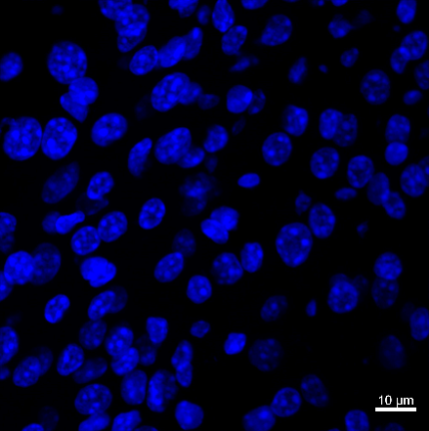

Supplement: Supplementary file 7 — Source Data for Figure 5 [file EMMM-15-e18526-s003.zip › Fig.5/Fig.5A/4months/PCMT1+-/Fig.1A_DAPI.tif]

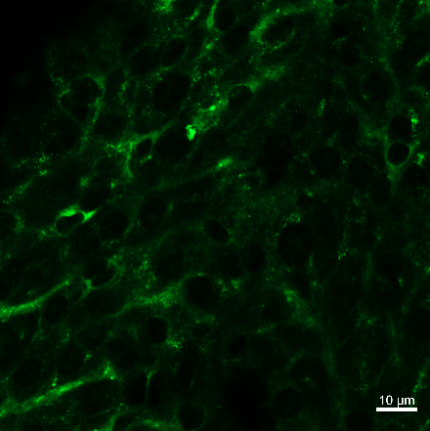

Supplement: Supplementary file 7 — Source Data for Figure 5 [file EMMM-15-e18526-s003.zip › Fig.5/Fig.5A/4months/PCMT1+-/Fig.1A_IsoDGR.tif]

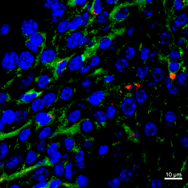

Supplement: Supplementary file 7 — Source Data for Figure 5 [file EMMM-15-e18526-s003.zip › Fig.5/Fig.5A/4months/PCMT1+-/Fig.1A_Merge.tif]
