# Supplementary figures and images for: Immunotherapy targeting isoDGR‐protein damage extends lifespan in a mouse model of protein deamidation (part 1 of 2)
Source: EMBO Mol Med. 2023 Nov 16;15(12):e18526. doi: 10.15252/emmm.202318526 (PMC10701600; doi:10.15252/emmm.202318526)

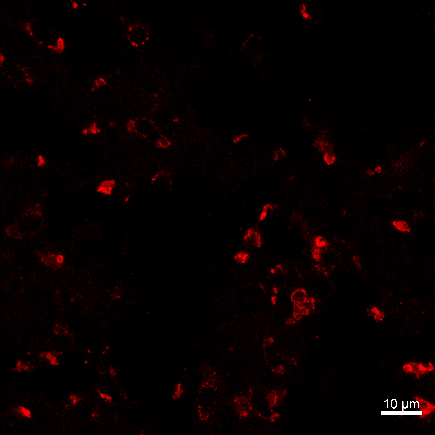

Supplement: Supplementary file 2 — Source Data for Appendix [file EMMM-15-e18526-s002.zip › Fig.S1/Fig.S1A/PCMT1++/Fig.S1_CD68.tif]

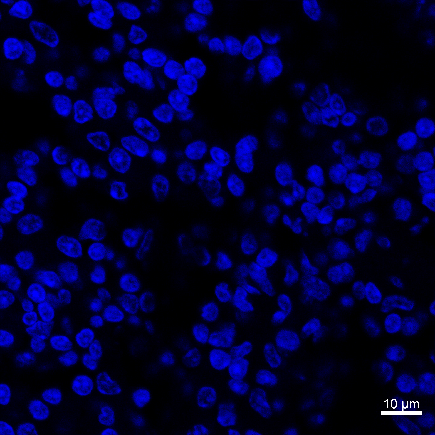

Supplement: Supplementary file 2 — Source Data for Appendix [file EMMM-15-e18526-s002.zip › Fig.S1/Fig.S1A/PCMT1++/Fig.S1_DAPI.tif]

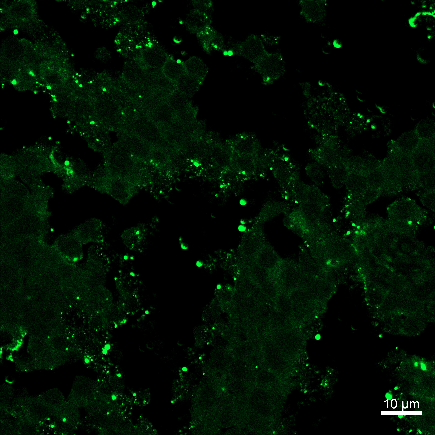

Supplement: Supplementary file 2 — Source Data for Appendix [file EMMM-15-e18526-s002.zip › Fig.S1/Fig.S1A/PCMT1++/Fig.S1_IsoDGR.tif]

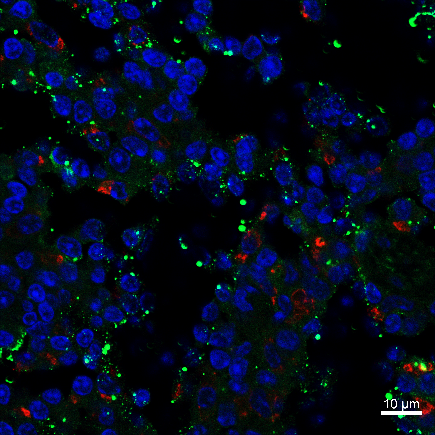

Supplement: Supplementary file 2 — Source Data for Appendix [file EMMM-15-e18526-s002.zip › Fig.S1/Fig.S1A/PCMT1++/Fig.S1_Merge.tif]

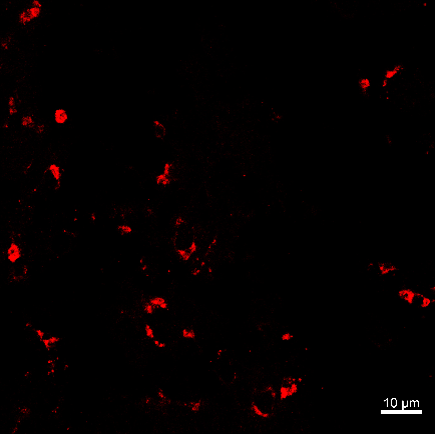

Supplement: Supplementary file 2 — Source Data for Appendix [file EMMM-15-e18526-s002.zip › Fig.S1/Fig.S1A/PCMT1+-/Fig.S1_CD68.tif]

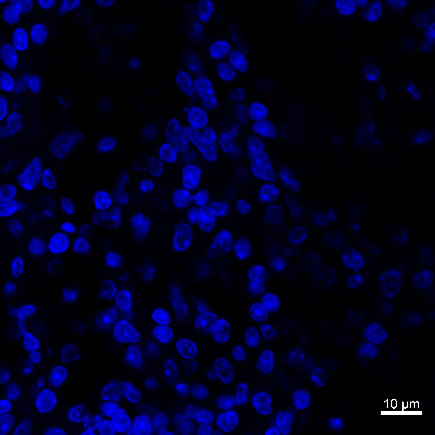

Supplement: Supplementary file 2 — Source Data for Appendix [file EMMM-15-e18526-s002.zip › Fig.S1/Fig.S1A/PCMT1+-/Fig.S1_DAPI.tif]

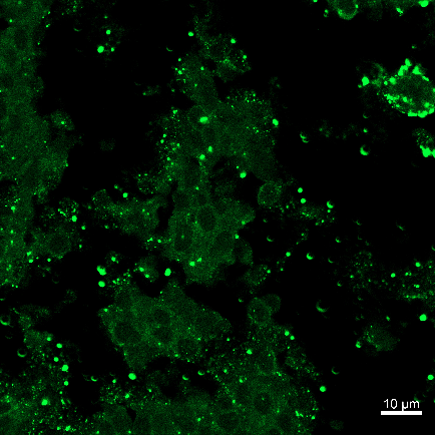

Supplement: Supplementary file 2 — Source Data for Appendix [file EMMM-15-e18526-s002.zip › Fig.S1/Fig.S1A/PCMT1+-/Fig.S1_IsoDGR.tif]

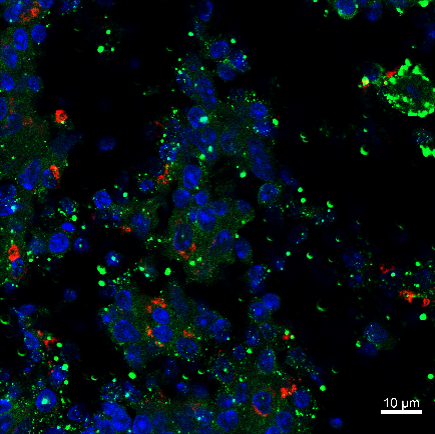

Supplement: Supplementary file 2 — Source Data for Appendix [file EMMM-15-e18526-s002.zip › Fig.S1/Fig.S1A/PCMT1+-/Fig.S1_Merge.tif]

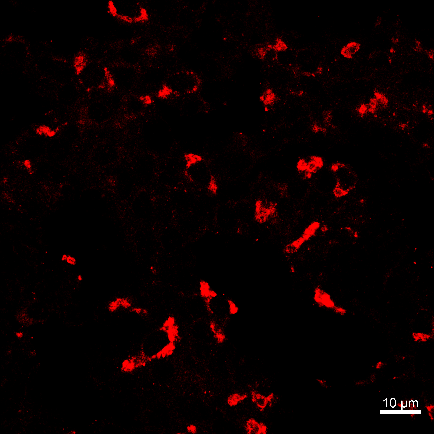

Supplement: Supplementary file 2 — Source Data for Appendix [file EMMM-15-e18526-s002.zip › Fig.S1/Fig.S1A/PCMT1--/Fig.S1_CD68.tif]

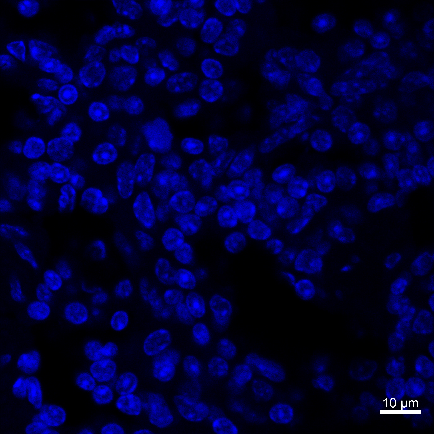

Supplement: Supplementary file 2 — Source Data for Appendix [file EMMM-15-e18526-s002.zip › Fig.S1/Fig.S1A/PCMT1--/Fig.S1_DAPI.tif]

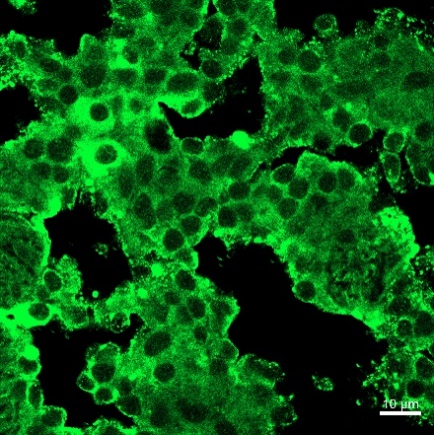

Supplement: Supplementary file 2 — Source Data for Appendix [file EMMM-15-e18526-s002.zip › Fig.S1/Fig.S1A/PCMT1--/Fig.S1_IsoDGR.tif]

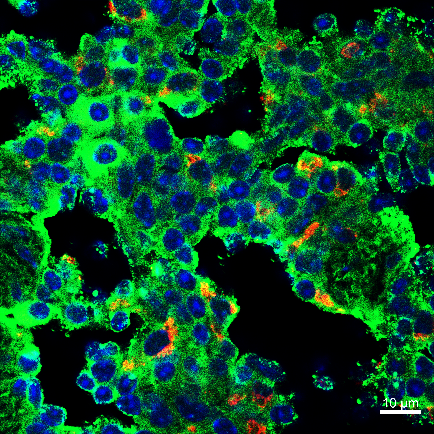

Supplement: Supplementary file 2 — Source Data for Appendix [file EMMM-15-e18526-s002.zip › Fig.S1/Fig.S1A/PCMT1--/Fig.S1_Merge.tif]

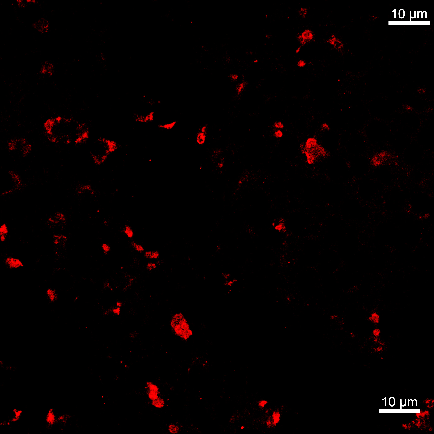

Supplement: Supplementary file 2 — Source Data for Appendix [file EMMM-15-e18526-s002.zip › Fig.S1/Fig.S1A/PCMT1--+_mAb/Fig.S1CD68.tif]

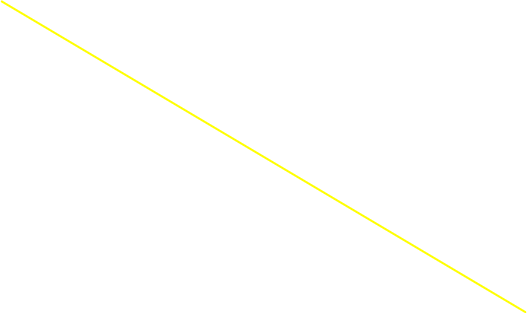

Supplement: Supplementary file 2 — Source Data for Appendix [file EMMM-15-e18526-s002.zip › Fig.S1/Fig.S1A/PCMT1--+_mAb/Fig.S1_CD68.tif]

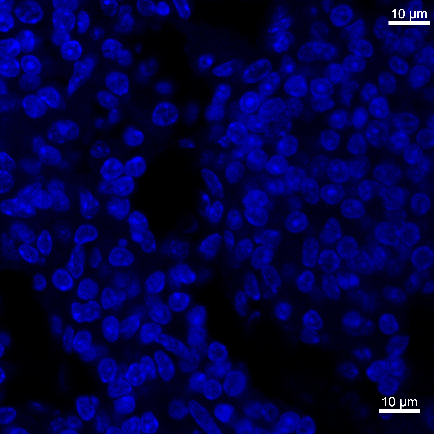

Supplement: Supplementary file 2 — Source Data for Appendix [file EMMM-15-e18526-s002.zip › Fig.S1/Fig.S1A/PCMT1--+_mAb/Fig.S1_DAPI.tif]

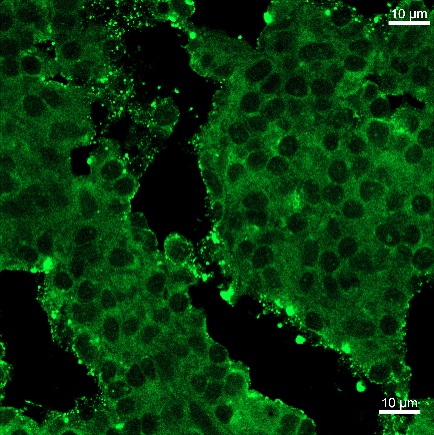

Supplement: Supplementary file 2 — Source Data for Appendix [file EMMM-15-e18526-s002.zip › Fig.S1/Fig.S1A/PCMT1--+_mAb/Fig.S1_IsoDGR.tif]

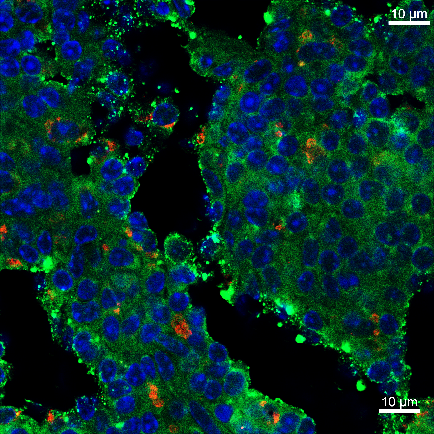

Supplement: Supplementary file 2 — Source Data for Appendix [file EMMM-15-e18526-s002.zip › Fig.S1/Fig.S1A/PCMT1--+_mAb/Fig.S1_Merge.tif]

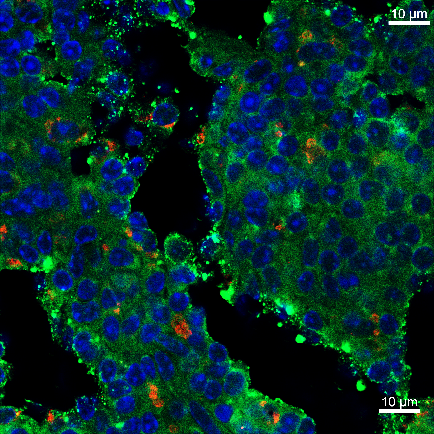

Supplement: Supplementary file 2 — Source Data for Appendix [file EMMM-15-e18526-s002.zip › Fig.S1/Fig.S1A/PCMT1--+_mAb/Fig.S1_Merge.tif.png]

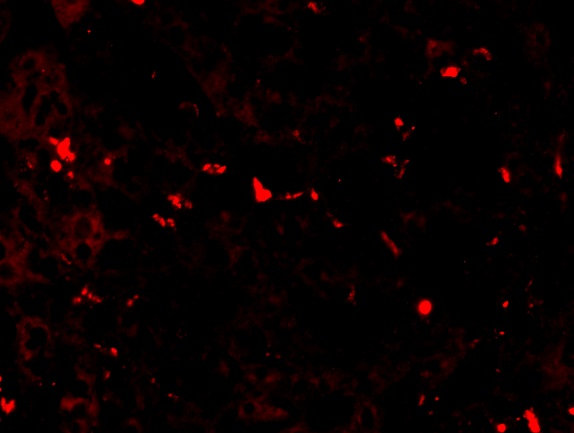

Supplement: Supplementary file 2 — Source Data for Appendix [file EMMM-15-e18526-s002.zip › Fig.S10/Fig.S10A/17_months_IgG/Fig.S10_CD68.tif]

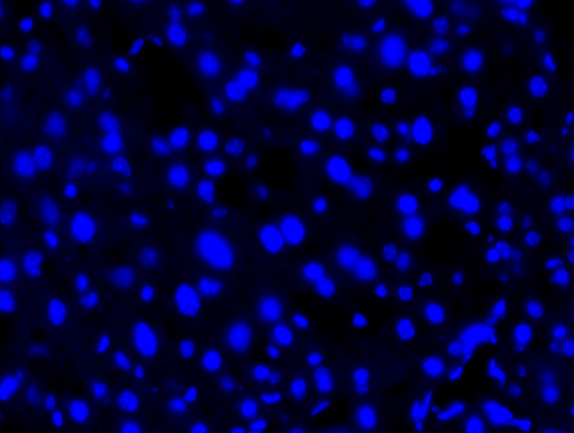

Supplement: Supplementary file 2 — Source Data for Appendix [file EMMM-15-e18526-s002.zip › Fig.S10/Fig.S10A/17_months_IgG/Fig.S10_DAPI.tif]

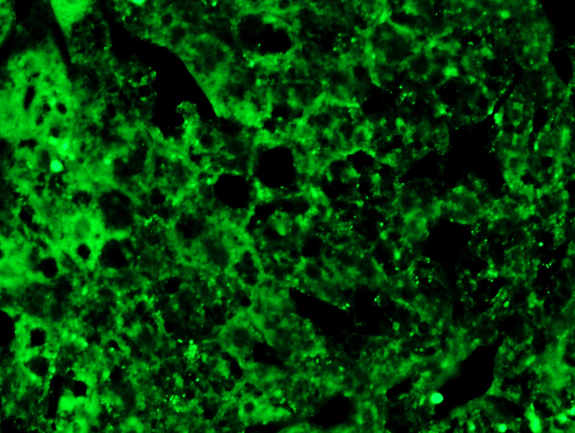

Supplement: Supplementary file 2 — Source Data for Appendix [file EMMM-15-e18526-s002.zip › Fig.S10/Fig.S10A/17_months_IgG/Fig.S10_IsoDGR.tif]

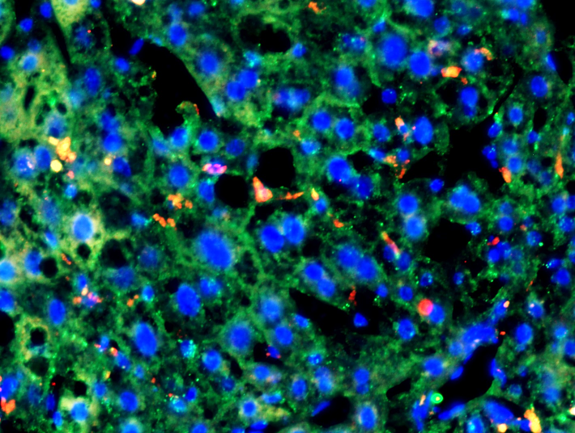

Supplement: Supplementary file 2 — Source Data for Appendix [file EMMM-15-e18526-s002.zip › Fig.S10/Fig.S10A/17_months_IgG/Fig.S10_Merge.tif]

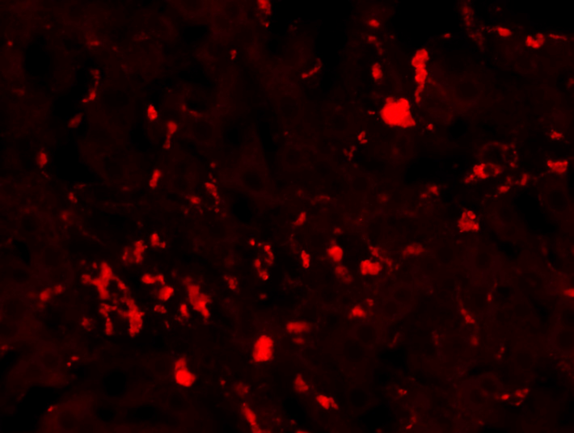

Supplement: Supplementary file 2 — Source Data for Appendix [file EMMM-15-e18526-s002.zip › Fig.S10/Fig.S10A/17_months_PBS/Fig.S10_CD68.png]

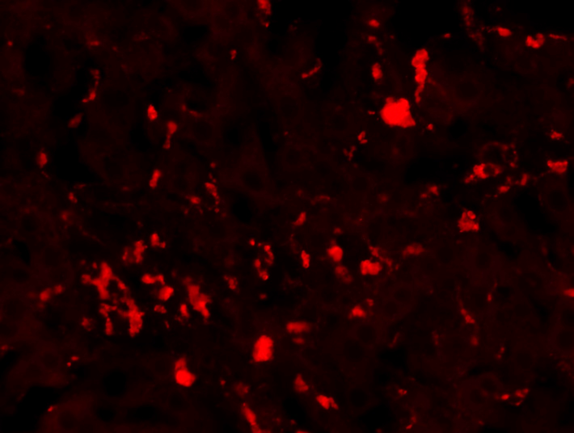

Supplement: Supplementary file 2 — Source Data for Appendix [file EMMM-15-e18526-s002.zip › Fig.S10/Fig.S10A/17_months_PBS/Fig.S10_CD68.tif]

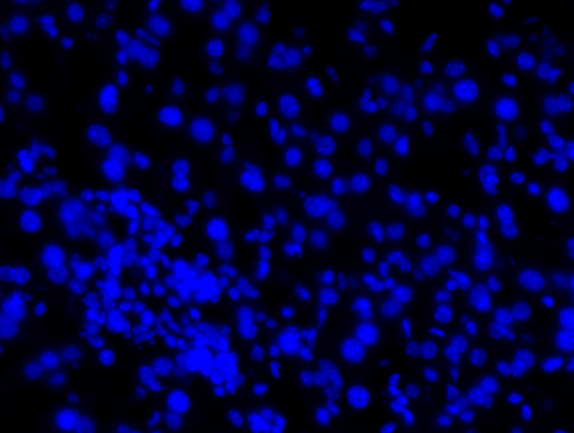

Supplement: Supplementary file 2 — Source Data for Appendix [file EMMM-15-e18526-s002.zip › Fig.S10/Fig.S10A/17_months_PBS/Fig.S10_DAPI.tif]

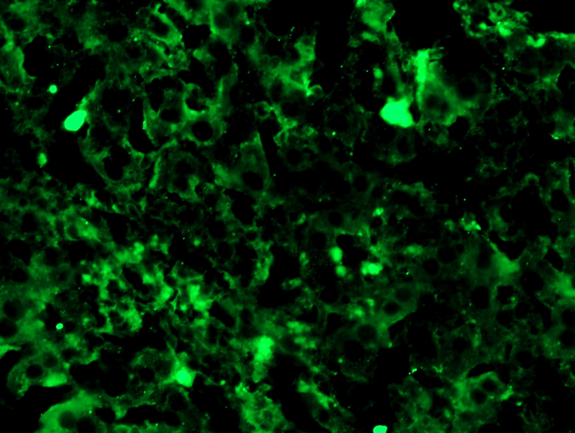

Supplement: Supplementary file 2 — Source Data for Appendix [file EMMM-15-e18526-s002.zip › Fig.S10/Fig.S10A/17_months_PBS/Fig.S10_IsoDGR.png]

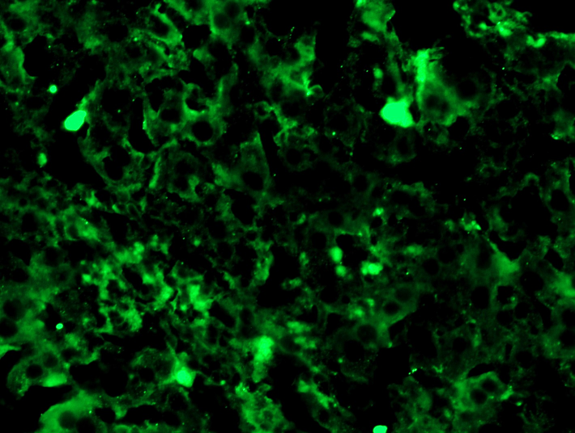

Supplement: Supplementary file 2 — Source Data for Appendix [file EMMM-15-e18526-s002.zip › Fig.S10/Fig.S10A/17_months_PBS/Fig.S10_IsoDGR.tif]

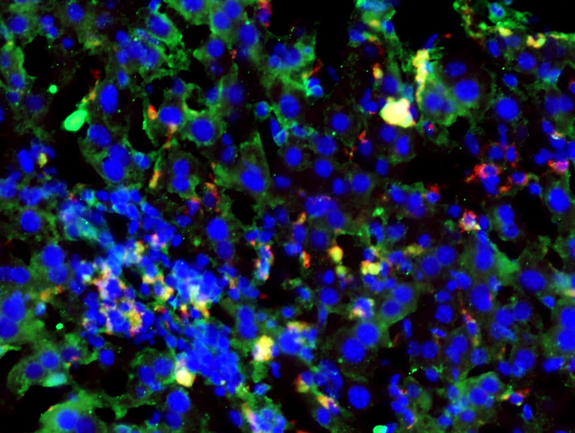

Supplement: Supplementary file 2 — Source Data for Appendix [file EMMM-15-e18526-s002.zip › Fig.S10/Fig.S10A/17_months_PBS/Fig.S10_Merge.tif]

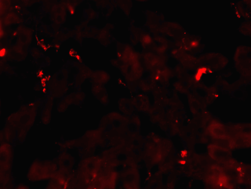

Supplement: Supplementary file 2 — Source Data for Appendix [file EMMM-15-e18526-s002.zip › Fig.S10/Fig.S10A/17_month_mAb/Fig.S10_CD68.tif]

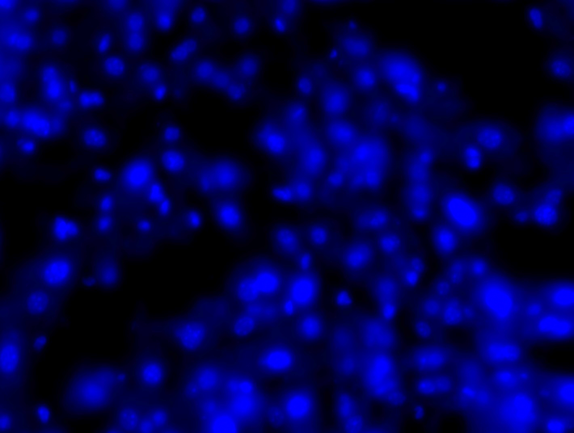

Supplement: Supplementary file 2 — Source Data for Appendix [file EMMM-15-e18526-s002.zip › Fig.S10/Fig.S10A/17_month_mAb/Fig.S10_DAPI.tif]

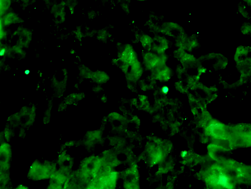

Supplement: Supplementary file 2 — Source Data for Appendix [file EMMM-15-e18526-s002.zip › Fig.S10/Fig.S10A/17_month_mAb/Fig.S10_IsoDGR.tif]

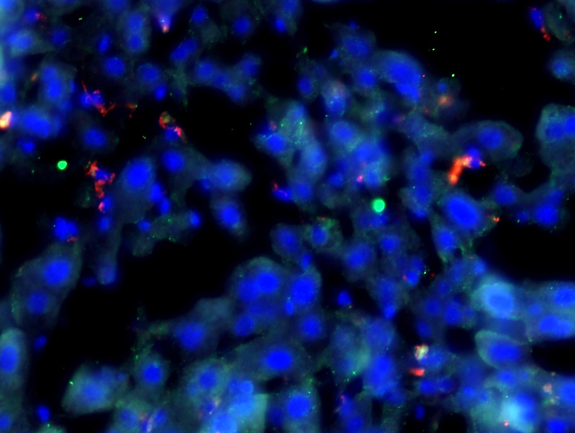

Supplement: Supplementary file 2 — Source Data for Appendix [file EMMM-15-e18526-s002.zip › Fig.S10/Fig.S10A/17_month_mAb/Fig.S10_Merge.tif]

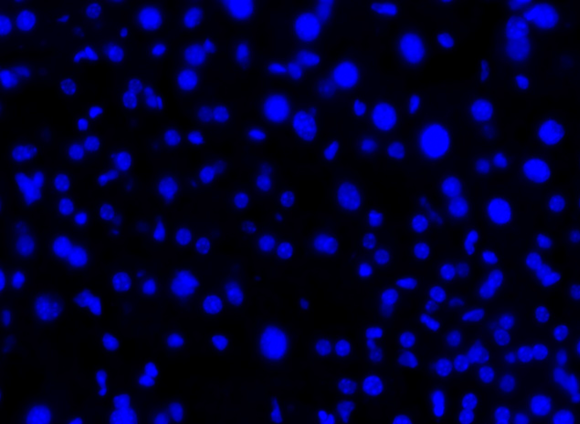

Supplement: Supplementary file 2 — Source Data for Appendix [file EMMM-15-e18526-s002.zip › Fig.S11/Fig.S11A/17months_(IgG)/Fig.S11_DAPI.tif]

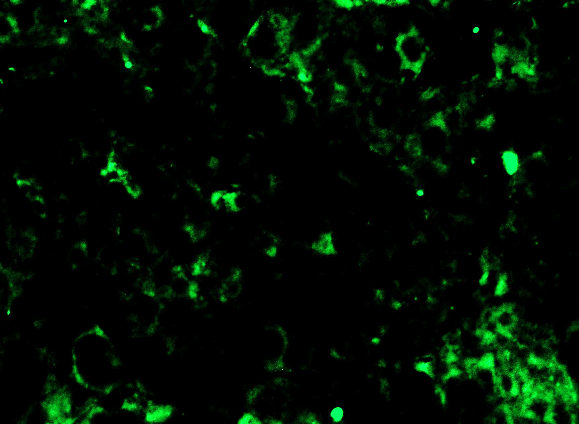

Supplement: Supplementary file 2 — Source Data for Appendix [file EMMM-15-e18526-s002.zip › Fig.S11/Fig.S11A/17months_(IgG)/Fig.S11_F4_80.tif]

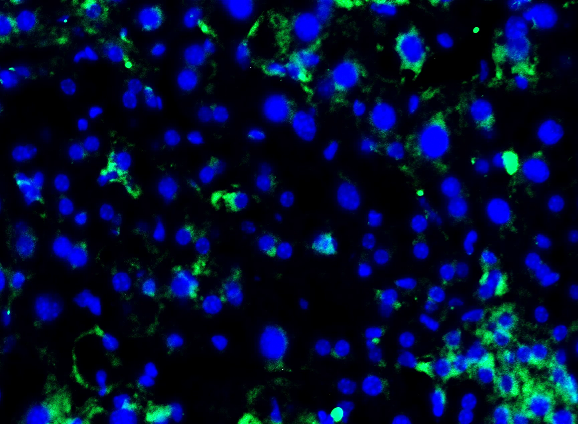

Supplement: Supplementary file 2 — Source Data for Appendix [file EMMM-15-e18526-s002.zip › Fig.S11/Fig.S11A/17months_(IgG)/Fig.S11_Merge.tif]

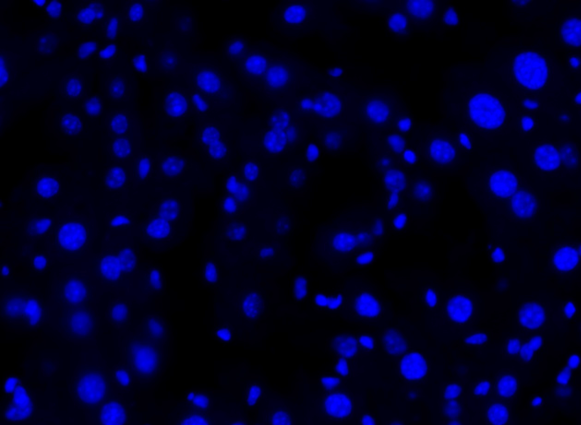

Supplement: Supplementary file 2 — Source Data for Appendix [file EMMM-15-e18526-s002.zip › Fig.S11/Fig.S11A/17months_(mAb)/Fig.S11_DAPI.tif]

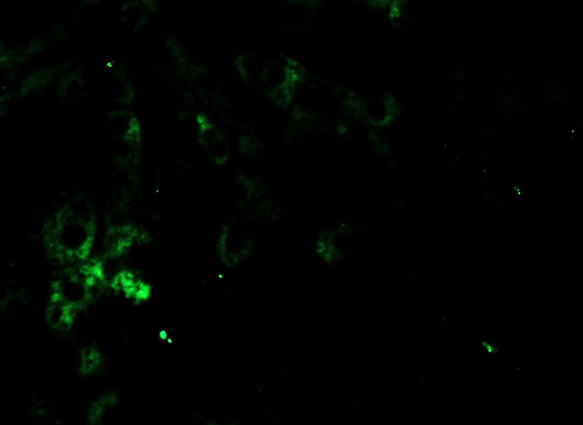

Supplement: Supplementary file 2 — Source Data for Appendix [file EMMM-15-e18526-s002.zip › Fig.S11/Fig.S11A/17months_(mAb)/Fig.S11_F4_80.tif]

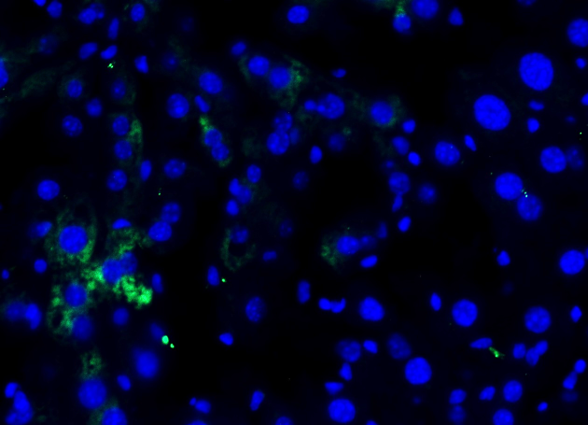

Supplement: Supplementary file 2 — Source Data for Appendix [file EMMM-15-e18526-s002.zip › Fig.S11/Fig.S11A/17months_(mAb)/Fig.S11_Merge.tif]

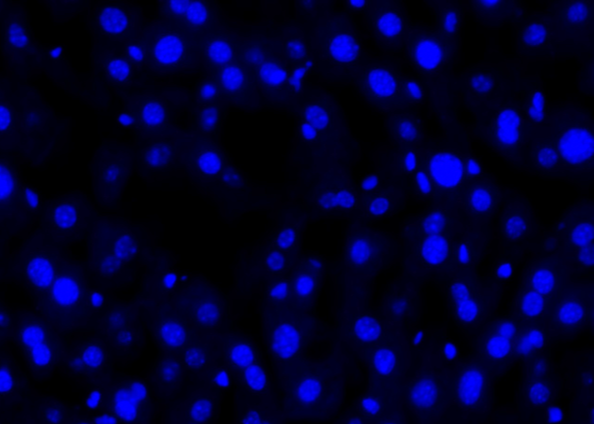

Supplement: Supplementary file 2 — Source Data for Appendix [file EMMM-15-e18526-s002.zip › Fig.S11/Fig.S11A/17months_(PBS)/Fig.S11_DAPI.tif]

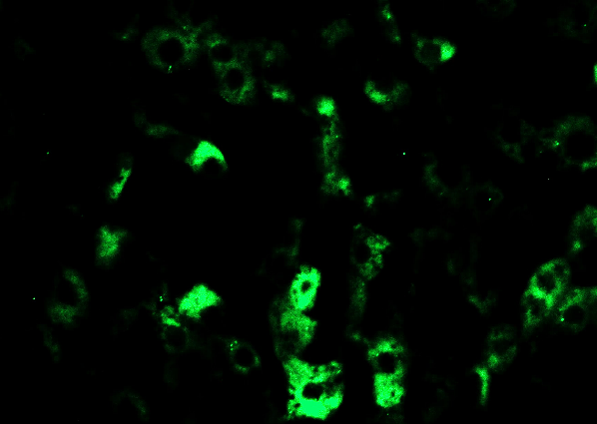

Supplement: Supplementary file 2 — Source Data for Appendix [file EMMM-15-e18526-s002.zip › Fig.S11/Fig.S11A/17months_(PBS)/Fig.S11_F4_80.tif]

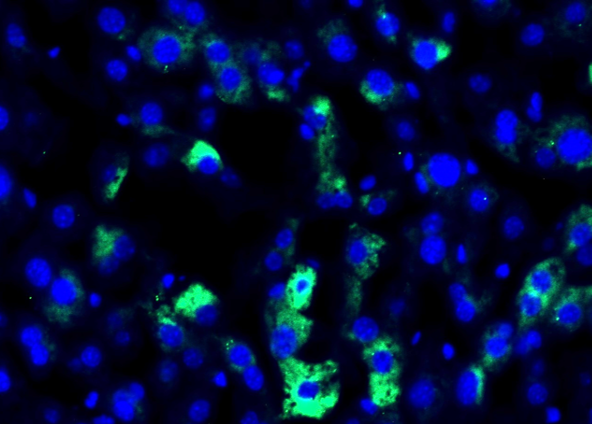

Supplement: Supplementary file 2 — Source Data for Appendix [file EMMM-15-e18526-s002.zip › Fig.S11/Fig.S11A/17months_(PBS)/Fig.S11_Merge.tif]

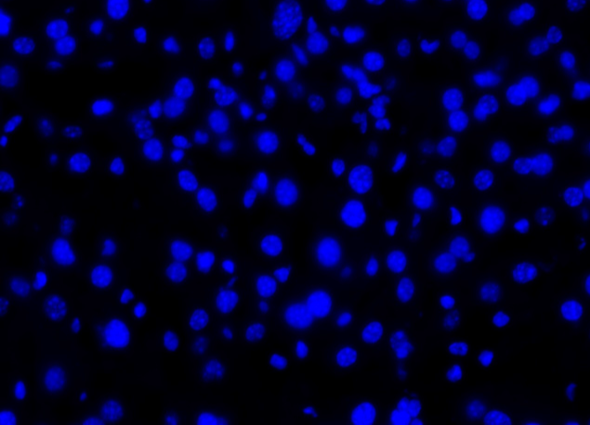

Supplement: Supplementary file 2 — Source Data for Appendix [file EMMM-15-e18526-s002.zip › Fig.S11/Fig.S11A/3months/Fig.S11_DAPI.tif]

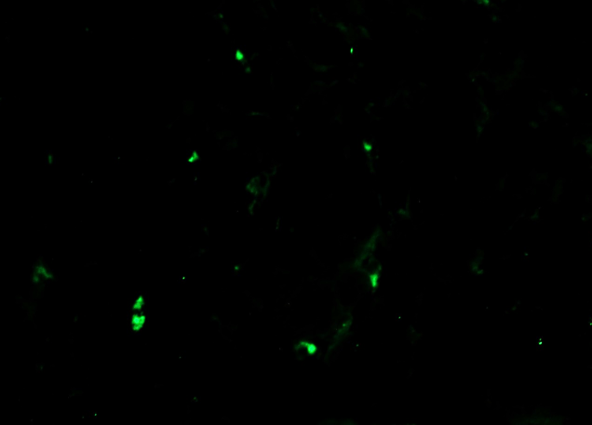

Supplement: Supplementary file 2 — Source Data for Appendix [file EMMM-15-e18526-s002.zip › Fig.S11/Fig.S11A/3months/Fig.S11_F4_80.tif]

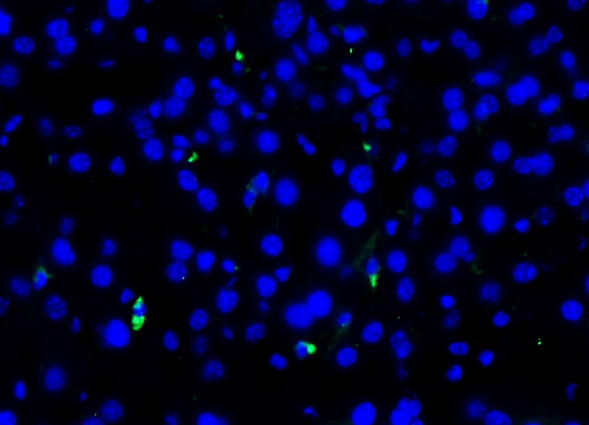

Supplement: Supplementary file 2 — Source Data for Appendix [file EMMM-15-e18526-s002.zip › Fig.S11/Fig.S11A/3months/Fig.S11_Merge.tif]

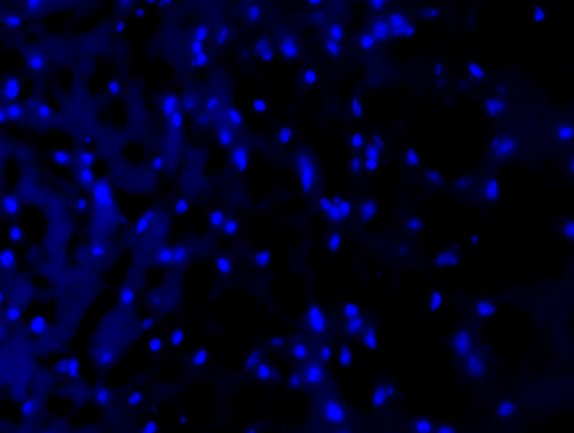

Supplement: Supplementary file 2 — Source Data for Appendix [file EMMM-15-e18526-s002.zip › Fig.S12/Fig.S12A/17month(mAb)/Fig.S12_DAPI.tif]

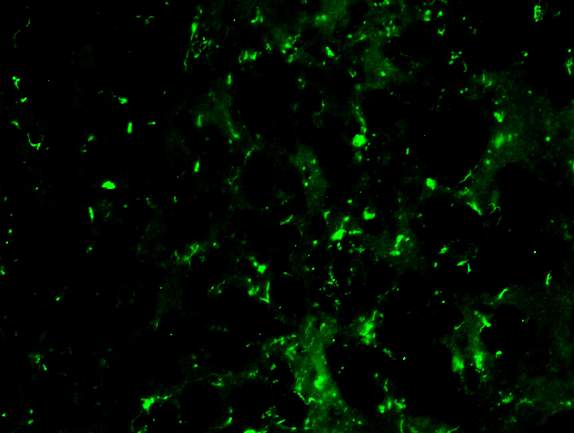

Supplement: Supplementary file 2 — Source Data for Appendix [file EMMM-15-e18526-s002.zip › Fig.S12/Fig.S12A/17month(mAb)/Fig.S12_IBA1.tif]

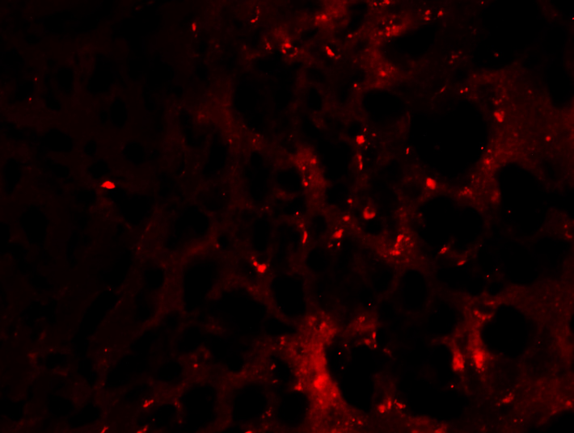

Supplement: Supplementary file 2 — Source Data for Appendix [file EMMM-15-e18526-s002.zip › Fig.S12/Fig.S12A/17month(mAb)/Fig.S12_IsoDGR.tif]

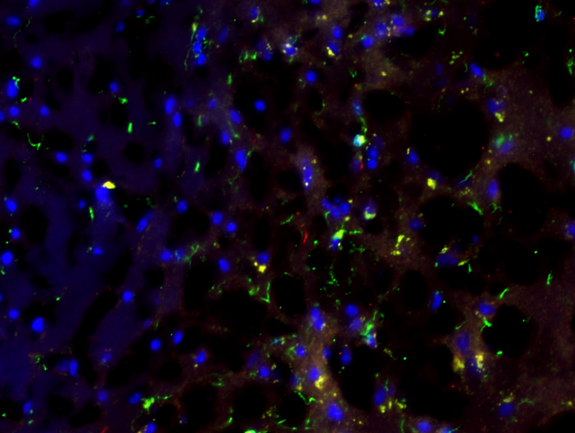

Supplement: Supplementary file 2 — Source Data for Appendix [file EMMM-15-e18526-s002.zip › Fig.S12/Fig.S12A/17month(mAb)/Fig.S12_Merge.tif]

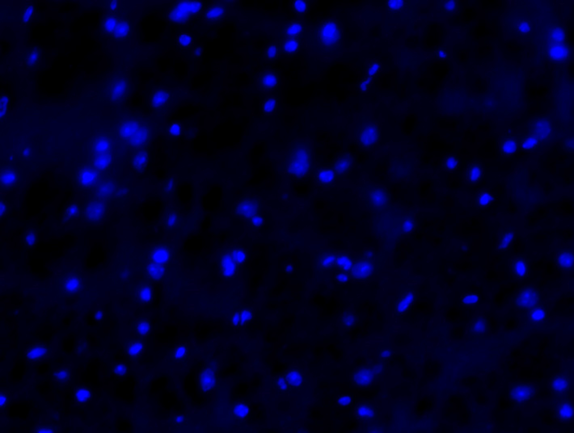

Supplement: Supplementary file 2 — Source Data for Appendix [file EMMM-15-e18526-s002.zip › Fig.S12/Fig.S12A/17month(PBS)/Fig.S12_DAPI.tif]

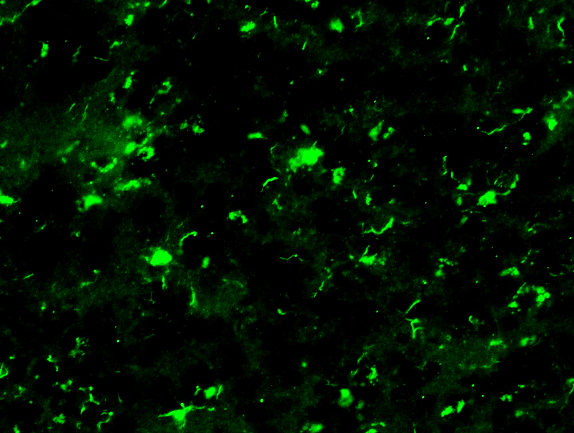

Supplement: Supplementary file 2 — Source Data for Appendix [file EMMM-15-e18526-s002.zip › Fig.S12/Fig.S12A/17month(PBS)/Fig.S12_IBA1.tif]

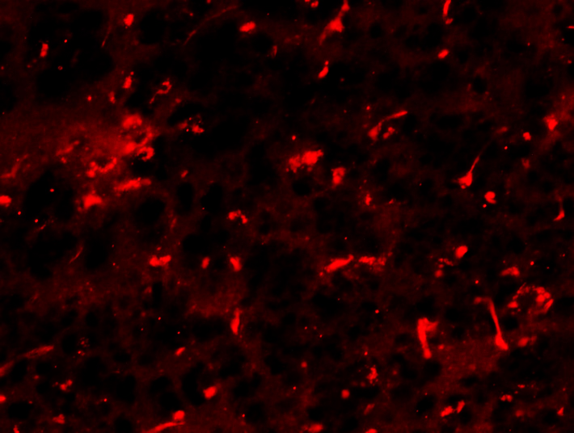

Supplement: Supplementary file 2 — Source Data for Appendix [file EMMM-15-e18526-s002.zip › Fig.S12/Fig.S12A/17month(PBS)/Fig.S12_IsoDGR.png]

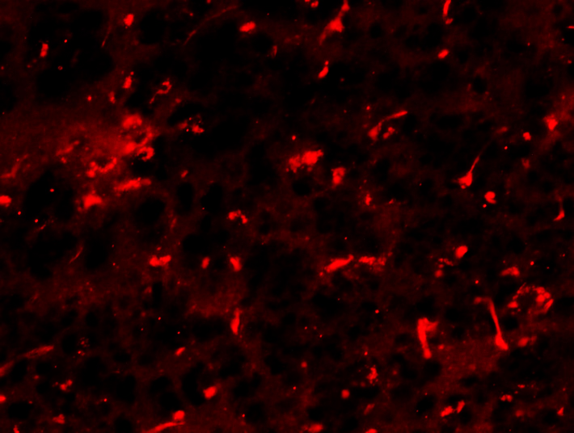

Supplement: Supplementary file 2 — Source Data for Appendix [file EMMM-15-e18526-s002.zip › Fig.S12/Fig.S12A/17month(PBS)/Fig.S12_IsoDGR.tif]

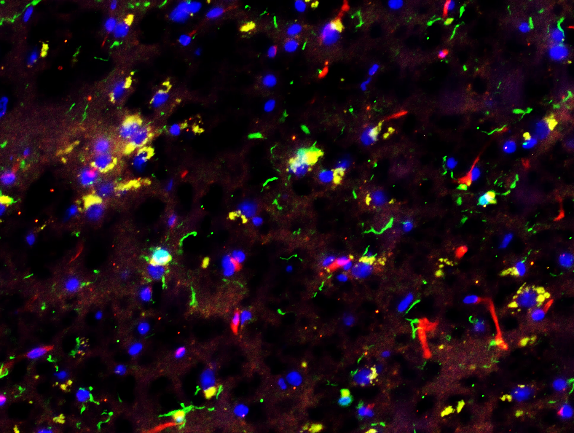

Supplement: Supplementary file 2 — Source Data for Appendix [file EMMM-15-e18526-s002.zip › Fig.S12/Fig.S12A/17month(PBS)/Fig.S12_Merge.tif]

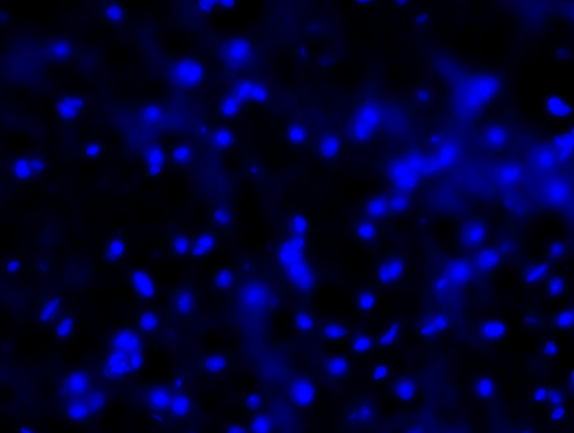

Supplement: Supplementary file 2 — Source Data for Appendix [file EMMM-15-e18526-s002.zip › Fig.S12/Fig.S12A/17month_(IgG)/Fig.S12_DAPI.tif]

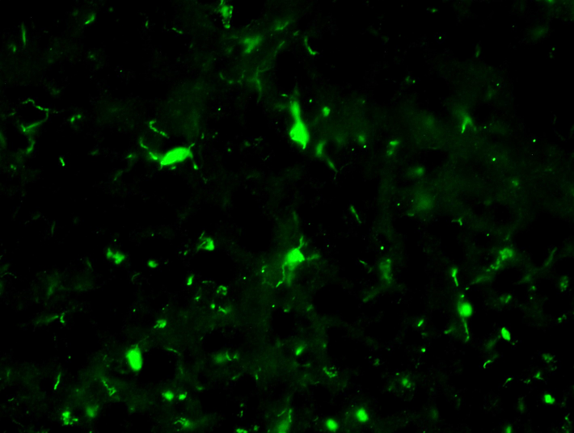

Supplement: Supplementary file 2 — Source Data for Appendix [file EMMM-15-e18526-s002.zip › Fig.S12/Fig.S12A/17month_(IgG)/Fig.S12_IBA1.tif]

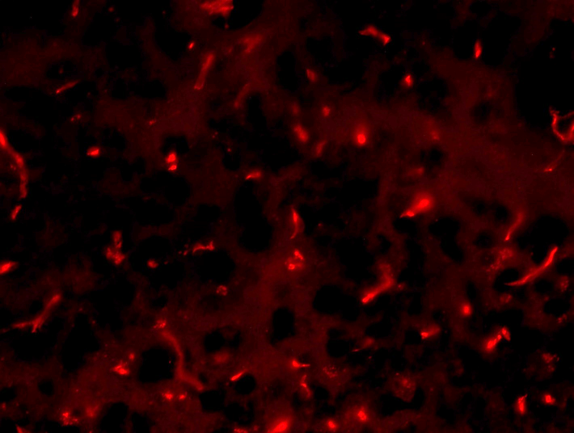

Supplement: Supplementary file 2 — Source Data for Appendix [file EMMM-15-e18526-s002.zip › Fig.S12/Fig.S12A/17month_(IgG)/Fig.S12_IsoDGR.png]

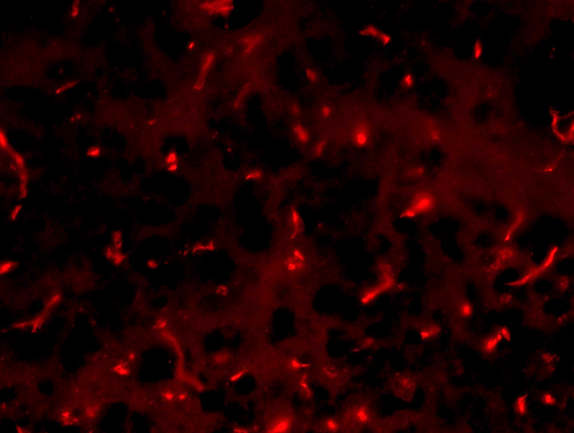

Supplement: Supplementary file 2 — Source Data for Appendix [file EMMM-15-e18526-s002.zip › Fig.S12/Fig.S12A/17month_(IgG)/Fig.S12_IsoDGR.tif]

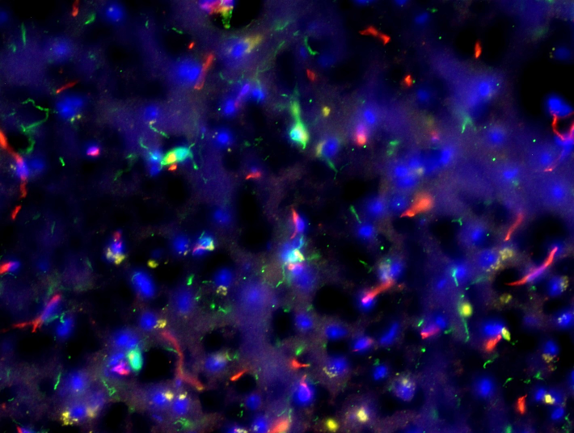

Supplement: Supplementary file 2 — Source Data for Appendix [file EMMM-15-e18526-s002.zip › Fig.S12/Fig.S12A/17month_(IgG)/Fig.S12_Merge.tif]

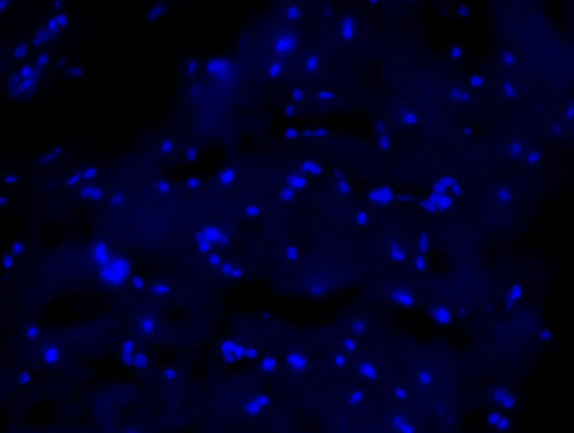

Supplement: Supplementary file 2 — Source Data for Appendix [file EMMM-15-e18526-s002.zip › Fig.S12/Fig.S12A/3month/Fig.S12_DAPI.tif]

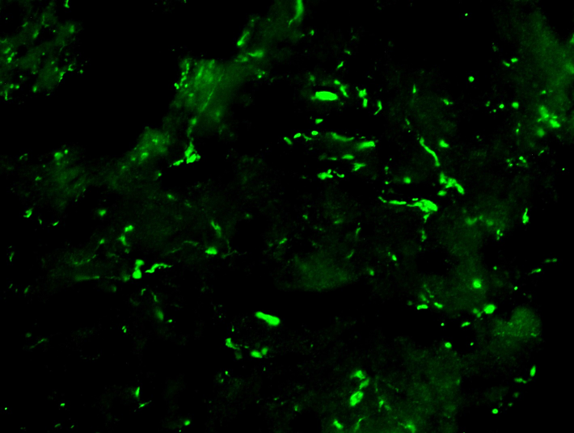

Supplement: Supplementary file 2 — Source Data for Appendix [file EMMM-15-e18526-s002.zip › Fig.S12/Fig.S12A/3month/Fig.S12_IBA1.tif]

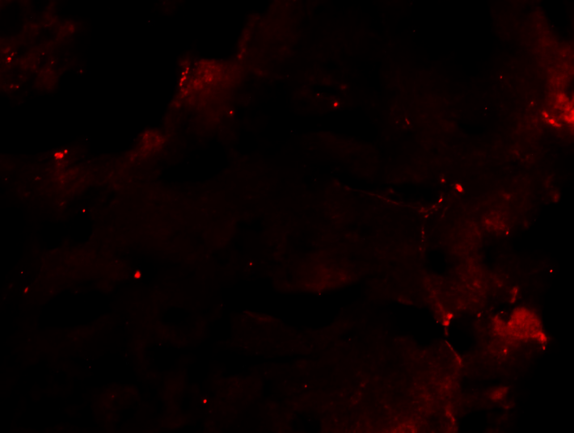

Supplement: Supplementary file 2 — Source Data for Appendix [file EMMM-15-e18526-s002.zip › Fig.S12/Fig.S12A/3month/Fig.S12_IsoDGR.tif]

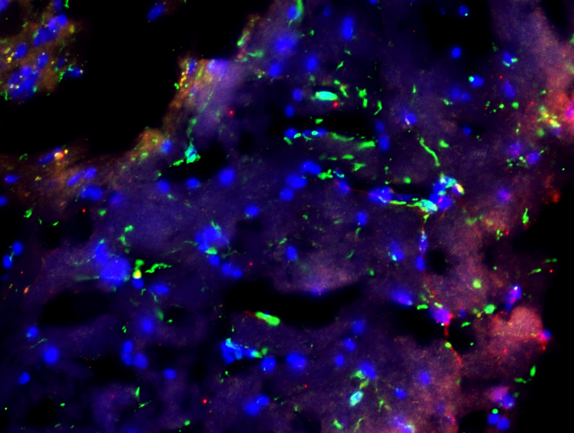

Supplement: Supplementary file 2 — Source Data for Appendix [file EMMM-15-e18526-s002.zip › Fig.S12/Fig.S12A/3month/Fig.S12_Merge.tif]

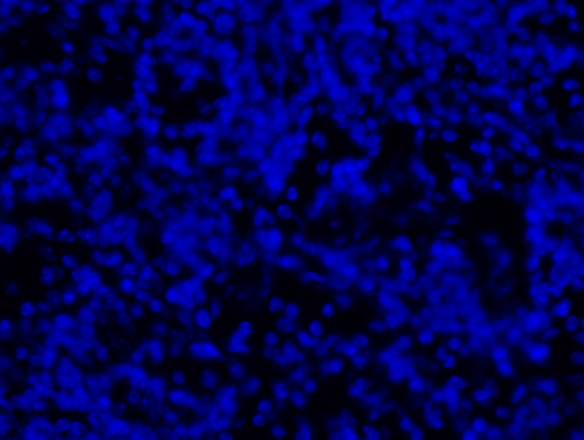

Supplement: Supplementary file 2 — Source Data for Appendix [file EMMM-15-e18526-s002.zip › Fig.S2/Fig.S2A/PCMT1++/Fig.S2_DAPI.tif]

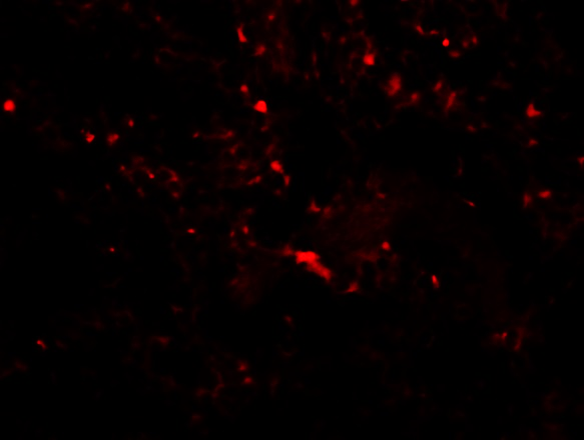

Supplement: Supplementary file 2 — Source Data for Appendix [file EMMM-15-e18526-s002.zip › Fig.S2/Fig.S2A/PCMT1++/Fig.S2_F4_80.tif]

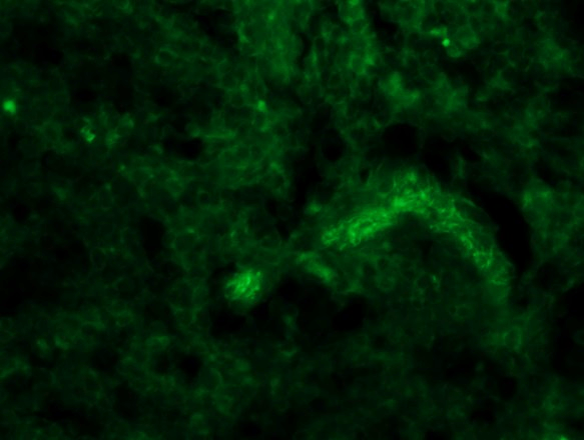

Supplement: Supplementary file 2 — Source Data for Appendix [file EMMM-15-e18526-s002.zip › Fig.S2/Fig.S2A/PCMT1++/Fig.S2_IsoDGR.tif]

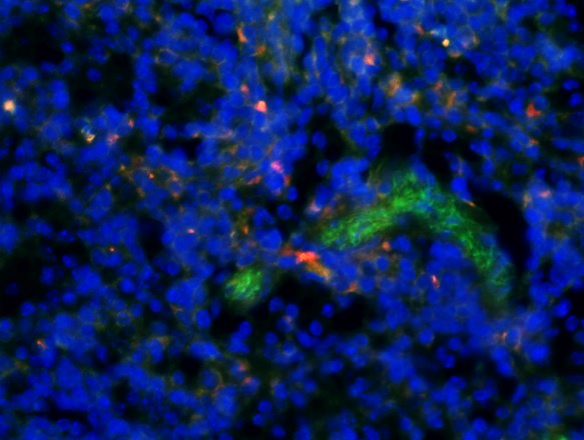

Supplement: Supplementary file 2 — Source Data for Appendix [file EMMM-15-e18526-s002.zip › Fig.S2/Fig.S2A/PCMT1++/Fig.S2_Merge.tif]

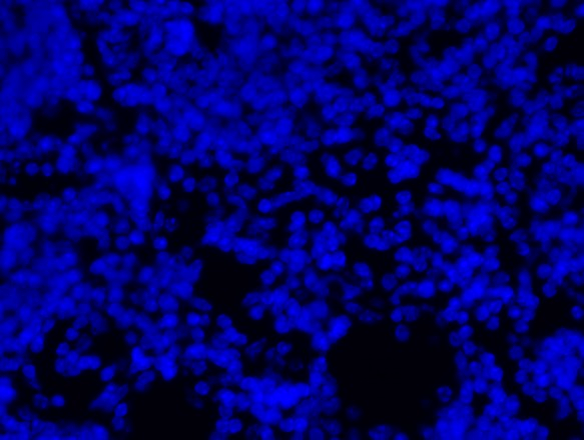

Supplement: Supplementary file 2 — Source Data for Appendix [file EMMM-15-e18526-s002.zip › Fig.S2/Fig.S2A/PCMT1--/Fig.S2_DAPI.tif]

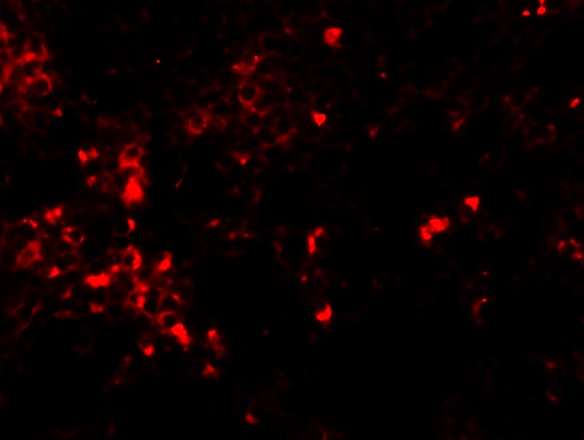

Supplement: Supplementary file 2 — Source Data for Appendix [file EMMM-15-e18526-s002.zip › Fig.S2/Fig.S2A/PCMT1--/Fig.S2_F4_80.tif]

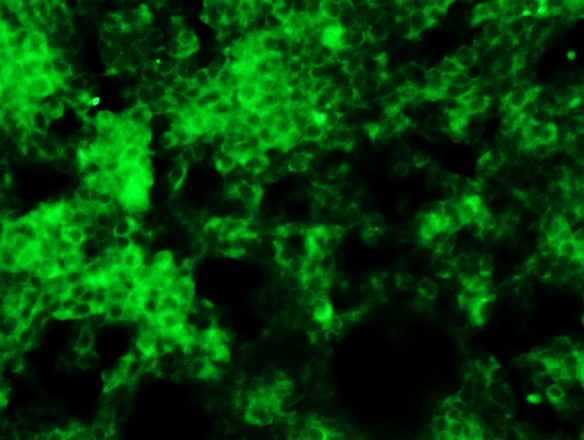

Supplement: Supplementary file 2 — Source Data for Appendix [file EMMM-15-e18526-s002.zip › Fig.S2/Fig.S2A/PCMT1--/Fig.S2_IsoDGR.tif]

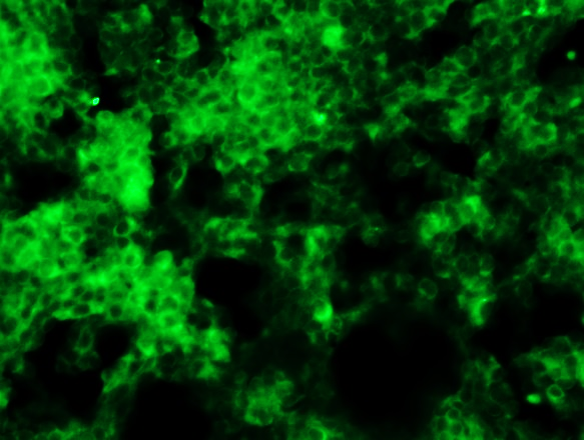

Supplement: Supplementary file 2 — Source Data for Appendix [file EMMM-15-e18526-s002.zip › Fig.S2/Fig.S2A/PCMT1--/Fig.S2_IsoDGR.tif.png]

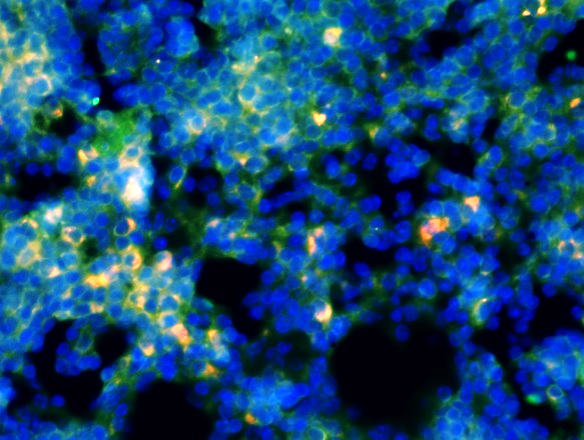

Supplement: Supplementary file 2 — Source Data for Appendix [file EMMM-15-e18526-s002.zip › Fig.S2/Fig.S2A/PCMT1--/Fig.S2_Merge.tif]

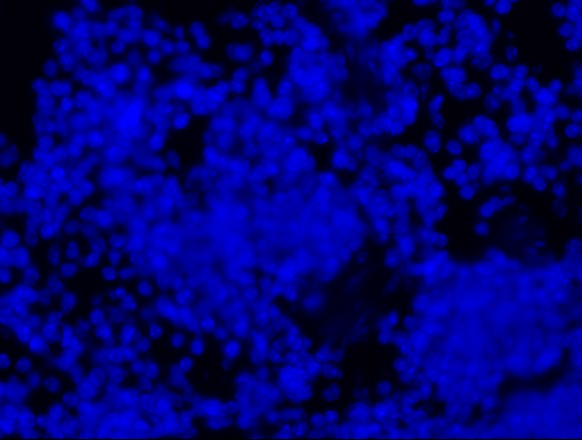

Supplement: Supplementary file 2 — Source Data for Appendix [file EMMM-15-e18526-s002.zip › Fig.S2/Fig.S2A/PCMT1--+mAb/Fig.S2._DAPI.tif]

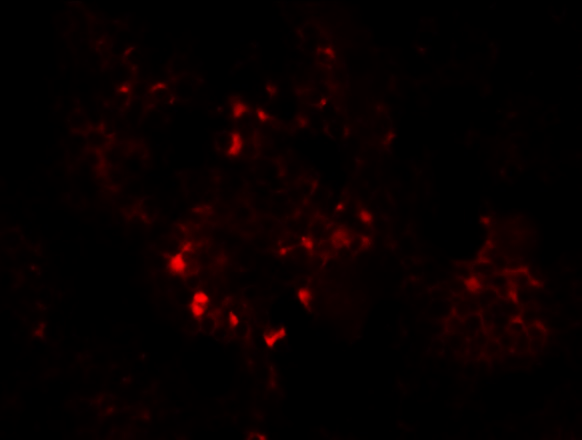

Supplement: Supplementary file 2 — Source Data for Appendix [file EMMM-15-e18526-s002.zip › Fig.S2/Fig.S2A/PCMT1--+mAb/Fig.S2_F4_80.tif]

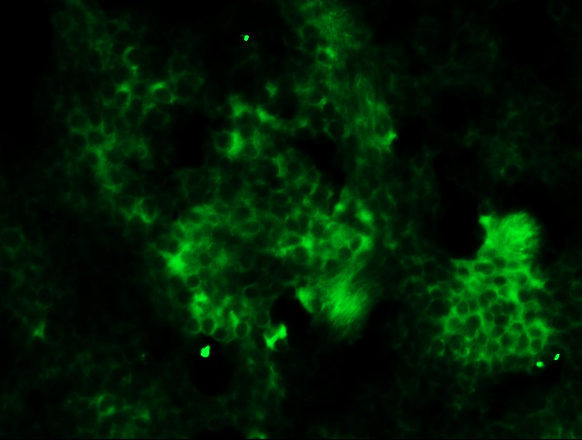

Supplement: Supplementary file 2 — Source Data for Appendix [file EMMM-15-e18526-s002.zip › Fig.S2/Fig.S2A/PCMT1--+mAb/Fig.S2_IsoDGR.tif]

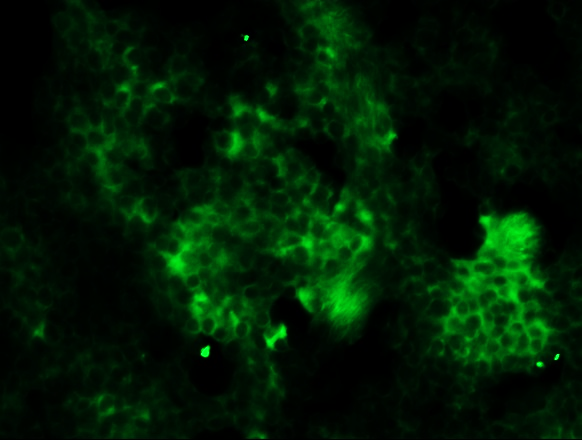

Supplement: Supplementary file 2 — Source Data for Appendix [file EMMM-15-e18526-s002.zip › Fig.S2/Fig.S2A/PCMT1--+mAb/Fig.S2_IsoDGR.tif.png]

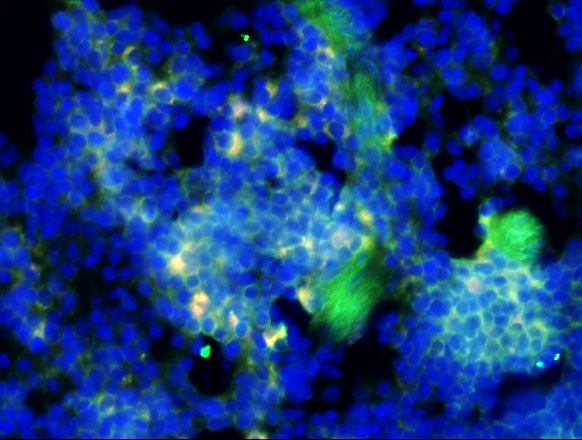

Supplement: Supplementary file 2 — Source Data for Appendix [file EMMM-15-e18526-s002.zip › Fig.S2/Fig.S2A/PCMT1--+mAb/Fig.S2_Merge.tif]

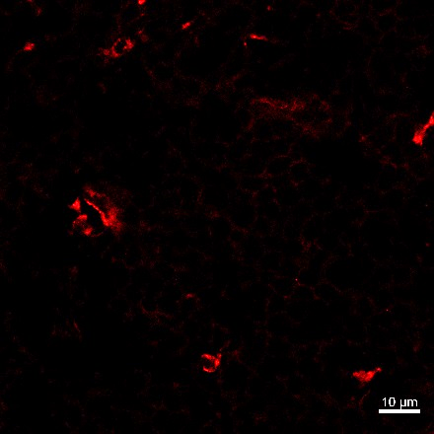

Supplement: Supplementary file 2 — Source Data for Appendix [file EMMM-15-e18526-s002.zip › Fig.S3/Fig.S3C_CD68.tif]

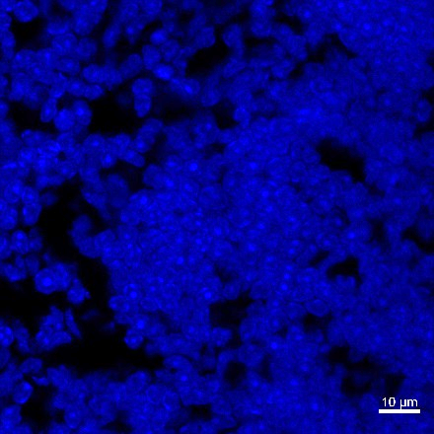

Supplement: Supplementary file 2 — Source Data for Appendix [file EMMM-15-e18526-s002.zip › Fig.S3/Fig.S3A/Fig.S3A_DAPI.tif]

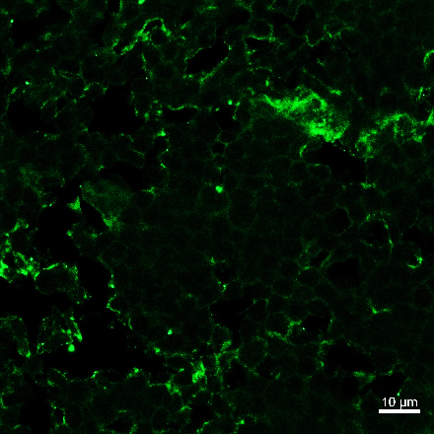

Supplement: Supplementary file 2 — Source Data for Appendix [file EMMM-15-e18526-s002.zip › Fig.S3/Fig.S3A/Fig.S3A_IsoDGR.tif]

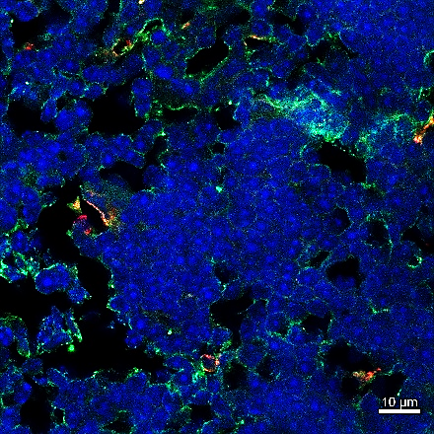

Supplement: Supplementary file 2 — Source Data for Appendix [file EMMM-15-e18526-s002.zip › Fig.S3/Fig.S3A/Fig.S3A_Merge.tif]

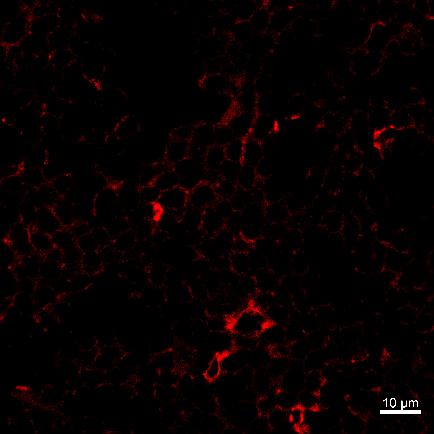

Supplement: Supplementary file 2 — Source Data for Appendix [file EMMM-15-e18526-s002.zip › Fig.S3/Fig.S3A/PCMT1+-/Fig.S3_CD68.tif]

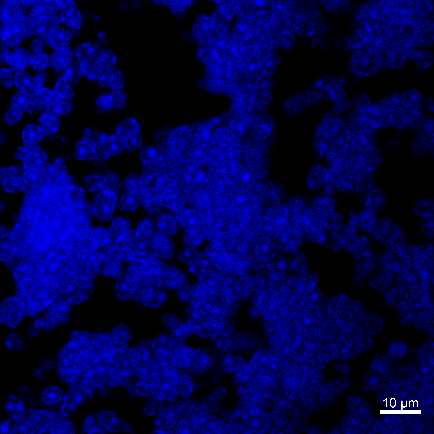

Supplement: Supplementary file 2 — Source Data for Appendix [file EMMM-15-e18526-s002.zip › Fig.S3/Fig.S3A/PCMT1+-/Fig.S3_DAPI.tif]

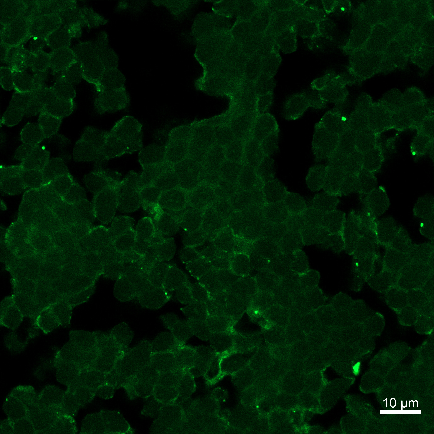

Supplement: Supplementary file 2 — Source Data for Appendix [file EMMM-15-e18526-s002.zip › Fig.S3/Fig.S3A/PCMT1+-/Fig.S3_IsoDGR.tif]

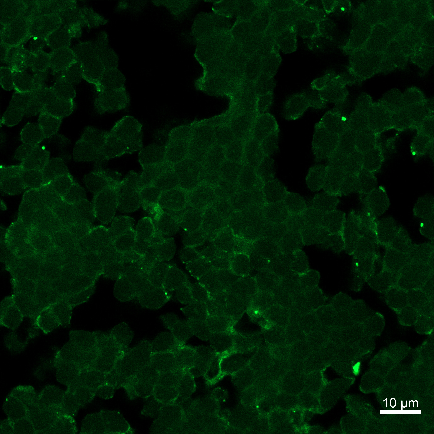

Supplement: Supplementary file 2 — Source Data for Appendix [file EMMM-15-e18526-s002.zip › Fig.S3/Fig.S3A/PCMT1+-/Fig.S3_IsoDGR.tif.png]

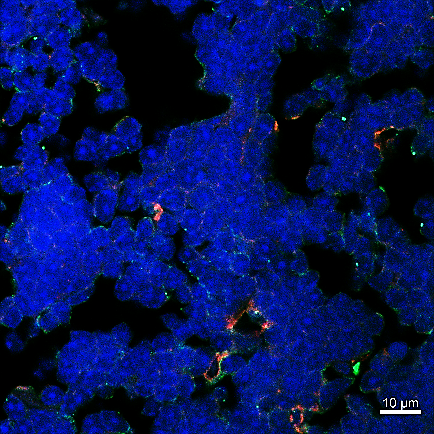

Supplement: Supplementary file 2 — Source Data for Appendix [file EMMM-15-e18526-s002.zip › Fig.S3/Fig.S3A/PCMT1+-/Fig.S3_Merge.tif]

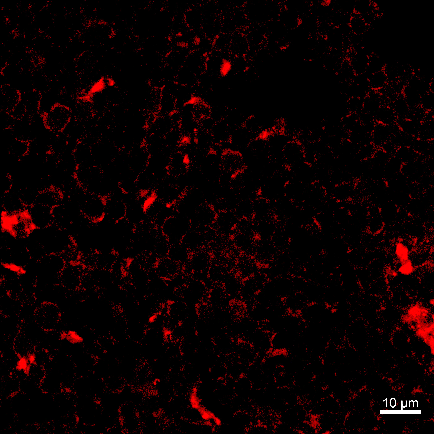

Supplement: Supplementary file 2 — Source Data for Appendix [file EMMM-15-e18526-s002.zip › Fig.S3/Fig.S3A/PCMT1--/Fig.S3_CD68.tif]

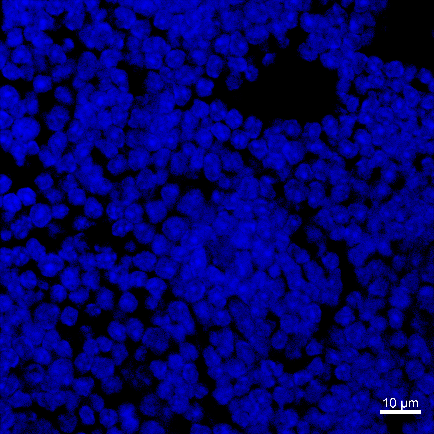

Supplement: Supplementary file 2 — Source Data for Appendix [file EMMM-15-e18526-s002.zip › Fig.S3/Fig.S3A/PCMT1--/Fig.S3_DAPI.tif]

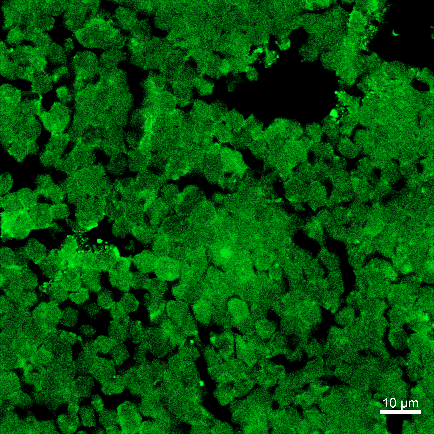

Supplement: Supplementary file 2 — Source Data for Appendix [file EMMM-15-e18526-s002.zip › Fig.S3/Fig.S3A/PCMT1--/Fig.S3_IsoDGR.tif]

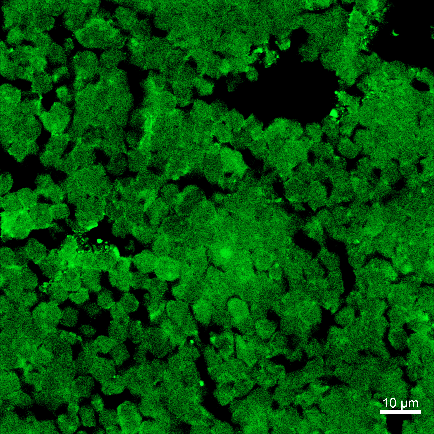

Supplement: Supplementary file 2 — Source Data for Appendix [file EMMM-15-e18526-s002.zip › Fig.S3/Fig.S3A/PCMT1--/Fig.S3_IsoDGR.tif.png]

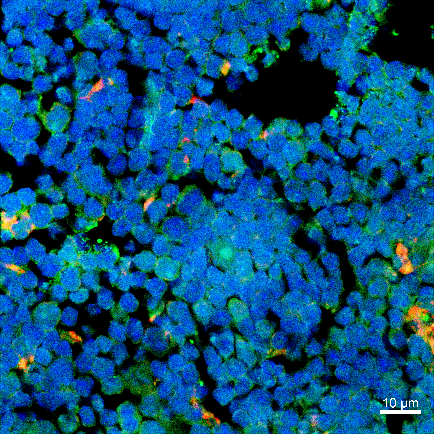

Supplement: Supplementary file 2 — Source Data for Appendix [file EMMM-15-e18526-s002.zip › Fig.S3/Fig.S3A/PCMT1--/Fig.S3_Merge.tif]

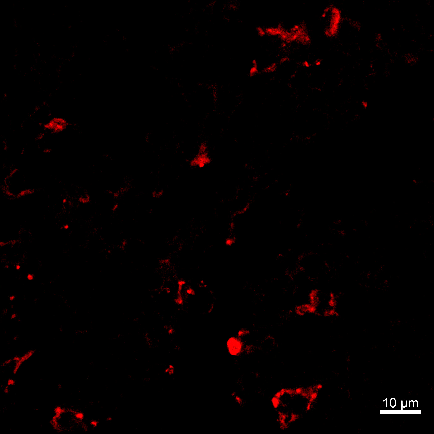

Supplement: Supplementary file 2 — Source Data for Appendix [file EMMM-15-e18526-s002.zip › Fig.S3/Fig.S3A/PCMT1--+mAb/Fig.S3_CD68.tif]

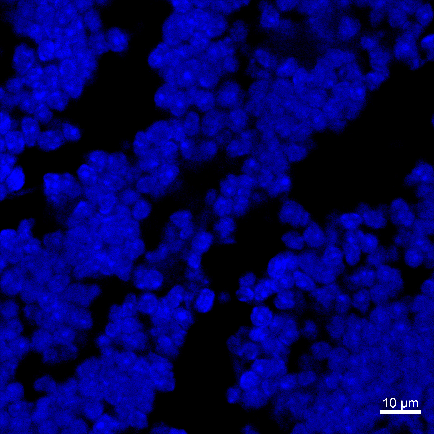

Supplement: Supplementary file 2 — Source Data for Appendix [file EMMM-15-e18526-s002.zip › Fig.S3/Fig.S3A/PCMT1--+mAb/Fig.S3_DAPI.tif]

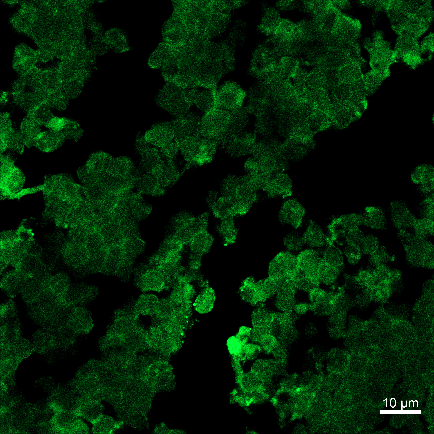

Supplement: Supplementary file 2 — Source Data for Appendix [file EMMM-15-e18526-s002.zip › Fig.S3/Fig.S3A/PCMT1--+mAb/Fig.S3_IsoDGR.tif]

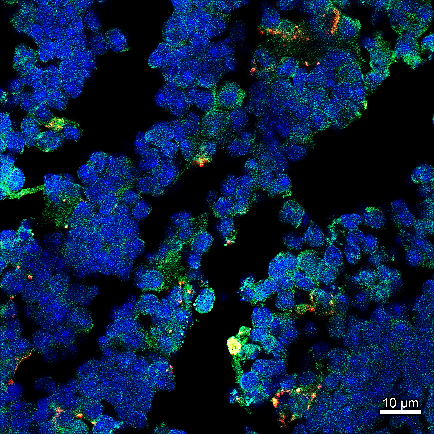

Supplement: Supplementary file 2 — Source Data for Appendix [file EMMM-15-e18526-s002.zip › Fig.S3/Fig.S3A/PCMT1--+mAb/Fig.S3_Merge.tif]

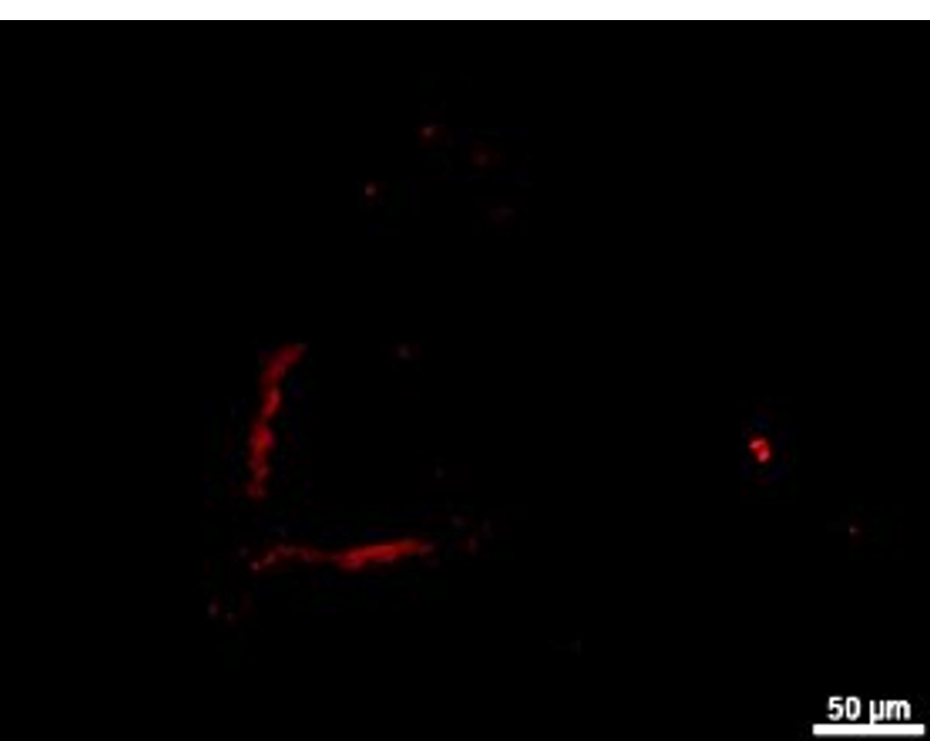

Supplement: Supplementary file 2 — Source Data for Appendix [file EMMM-15-e18526-s002.zip › Fig.S4/Fig.S4B/Fig.4B_isoDGR_modified_Plasma/Fig.S4B_CD68.tif]

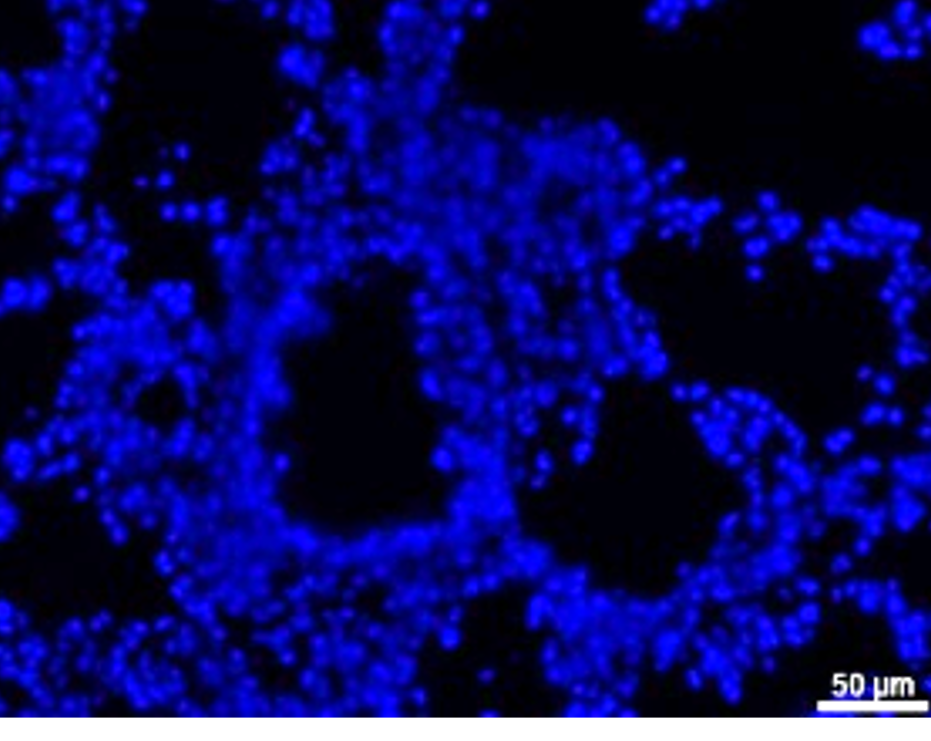

Supplement: Supplementary file 2 — Source Data for Appendix [file EMMM-15-e18526-s002.zip › Fig.S4/Fig.S4B/Fig.4B_isoDGR_modified_Plasma/Fig.S4B_DAPI.tif]

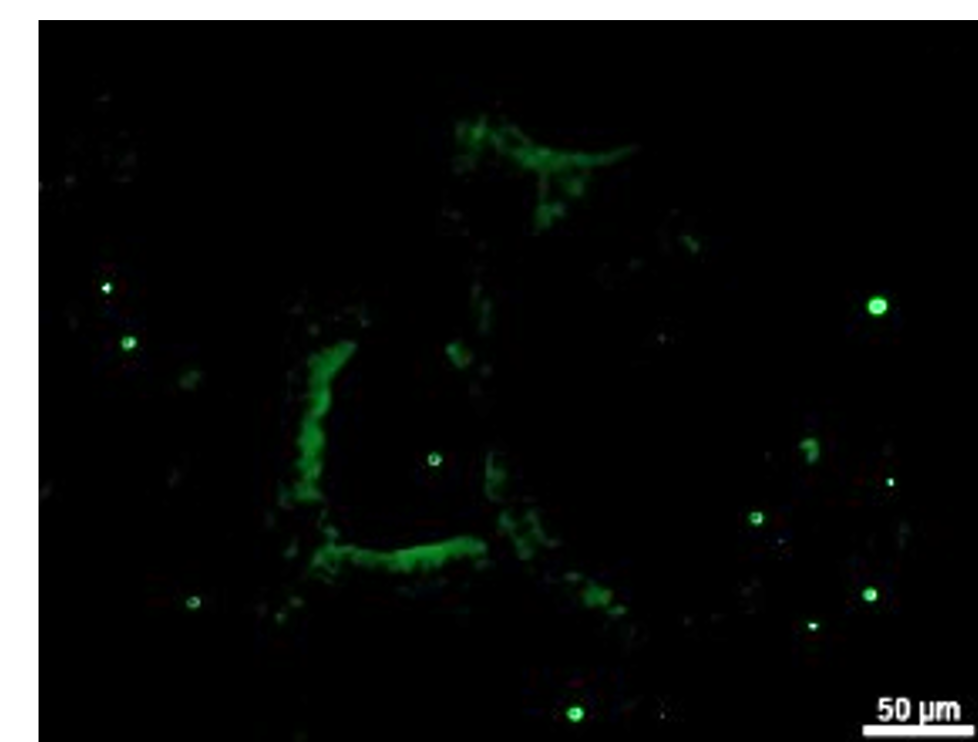

Supplement: Supplementary file 2 — Source Data for Appendix [file EMMM-15-e18526-s002.zip › Fig.S4/Fig.S4B/Fig.4B_isoDGR_modified_Plasma/Fig.S4B_IsoDGR.tif]

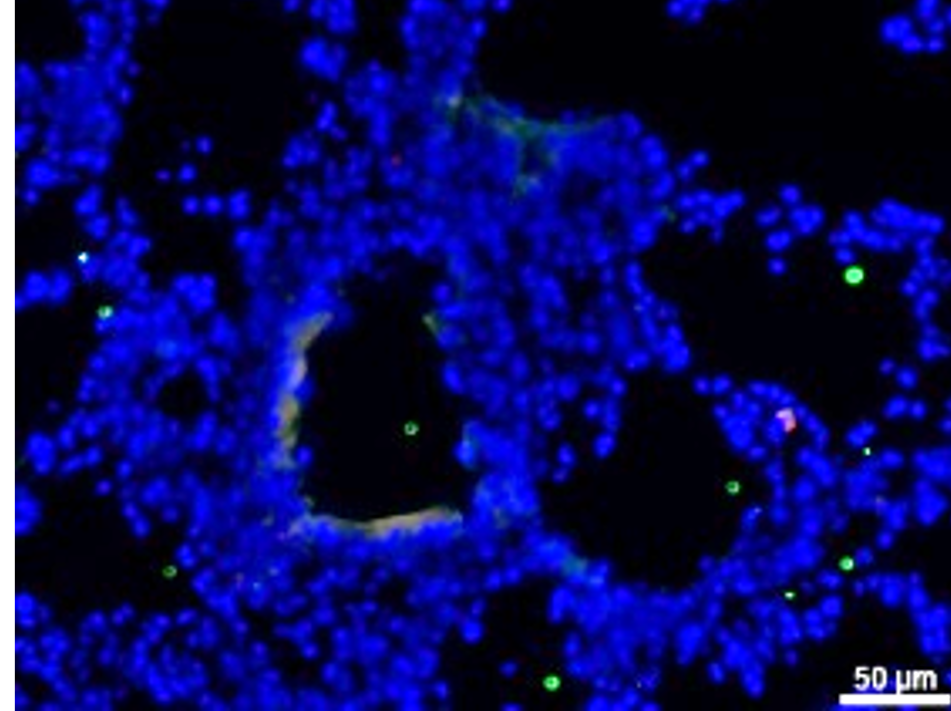

Supplement: Supplementary file 2 — Source Data for Appendix [file EMMM-15-e18526-s002.zip › Fig.S4/Fig.S4B/Fig.4B_isoDGR_modified_Plasma/Fig.S4B_Merge.tif]

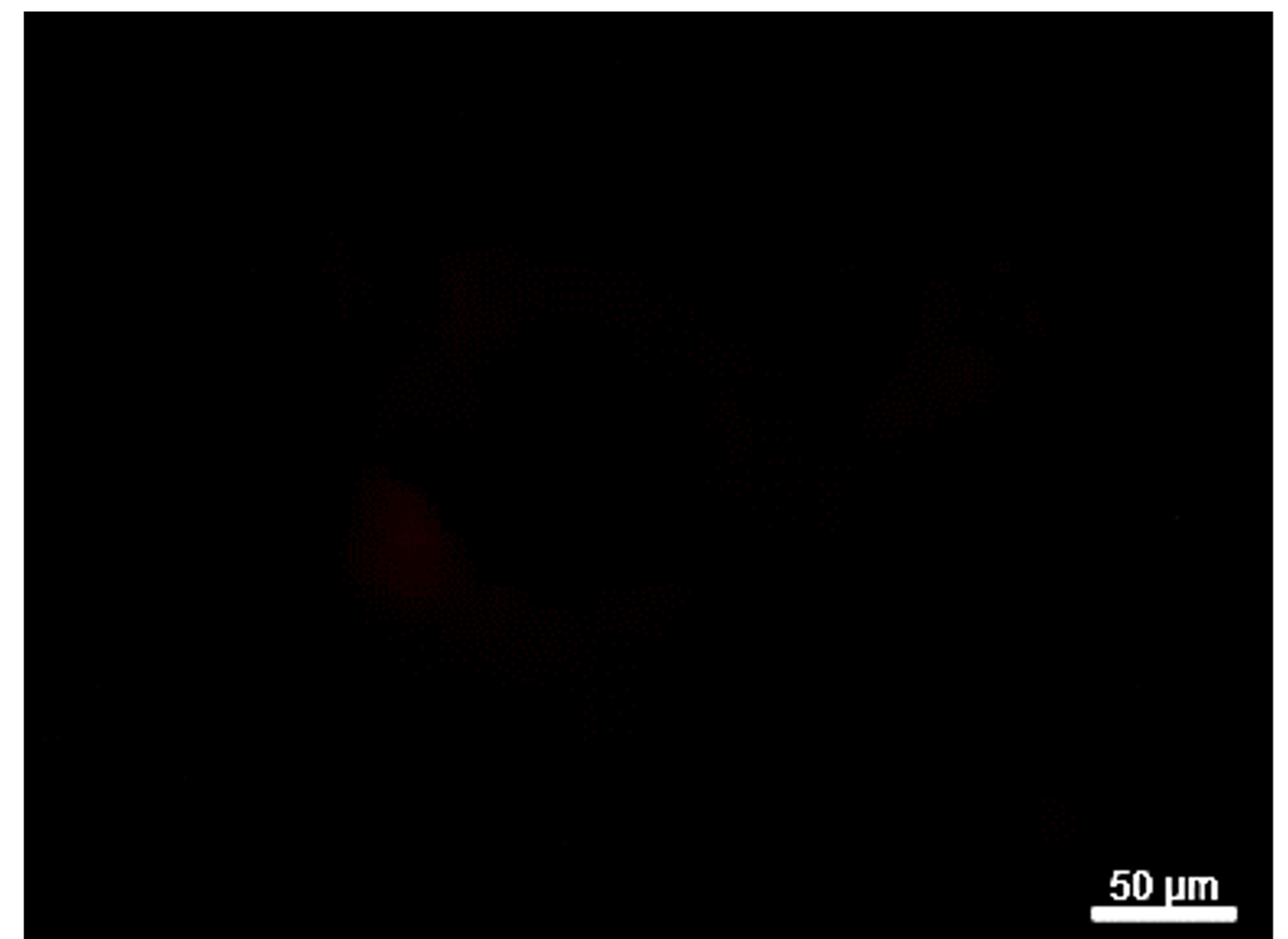

Supplement: Supplementary file 2 — Source Data for Appendix [file EMMM-15-e18526-s002.zip › Fig.S4/Fig.S4B/Fig.4B_WT_plasma/Fig.S4B_CD68.tif]

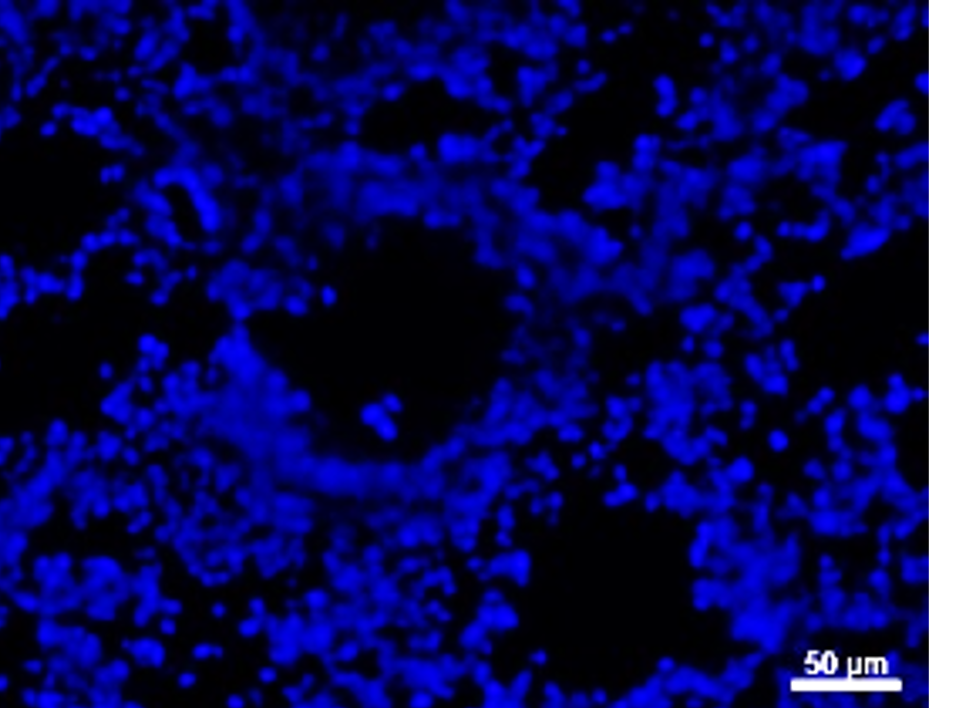

Supplement: Supplementary file 2 — Source Data for Appendix [file EMMM-15-e18526-s002.zip › Fig.S4/Fig.S4B/Fig.4B_WT_plasma/Fig.S4B_DAPI.tif]
