# Supplementary material for: Immunotherapy targeting isoDGR‐protein damage extends lifespan in a mouse model of protein deamidation
Source: EMBO Mol Med. 2023 Nov 16;15(12):e18526. doi: 10.15252/emmm.202318526 (PMC10701600; doi:10.15252/emmm.202318526)
Supplement: Supplementary file 10 [file EMMM-15-e18526-s008.pdf]

EMBO Molecular Medicine

Immunotherapy targeting isoDGR-protein damage Extends Lifespan in a Mouse Model of Protein Deamidation

Siu Kwan Sze, Pazhanichamy Kalailingam, Khalilatul-Hanisah Mohd-Kahliab, SoFong Ngan, Ranjith Iyappan, Evelin Melekh, Tian Lu, Gan Wei Zien, Bhargy Sharma, Tiannan Guo, Adam MacNeil, Rebecca MacPherson, Evangelia Tsiani, Deborah O'Leary, Kah Leong Lim, I-hsin Su, Yong-Gui Gao, Arthur Richards, Raj Kalaria, Christopher Chen, and Neil McCarthy
DOI: 10.15252/emmm.202318526

Corresponding author: Siu Kwan Sze (nsze@brocku.ca)

Review Timeline:

Submission Date:	17th Aug 23
Editorial Decision:	12th Sep 23
Revision Received:	17th Sep 23
Editorial Decision:	18th Oct 23
Revision Received:	21st Oct 23
Accepted:	31st Oct 23

Editor: Poonam Bheda

Transaction Report:

(Note: With the exception of the correction of typographical or spelling errors that could be a source of ambiguity, letters and reports are not edited. Depending on transfer agreements, referee reports obtained elsewhere may or may not be included in this compilation. Referee reports are anonymous unless the Referee chooses to sign their reports.)

12th Sep 2023

Dear Prof. Sze,

Thank you for submitting your work to EMBO Molecular Medicine. We have now heard back from the referees who agreed to evaluate your manuscript, including Reviewer 2 who previously reviewed the manuscript, and a new Reviewer 1. As you will see below, while the reviewers acknowledge the potential interest of the work, there remain substantial concerns that need to be addressed in full for further consideration of the manuscript. In particular, the development and validation of the isoDGR antibody for specificity should be clear and included in the study. In addition, the claims on the effect of the isoDGR antibody on extending lifespan will need to be toned down in the title and abstract to reflect the fact that Pcmt1 knockout mice have a substantial decrease in lifespan.

Addressing the reviewers' concerns in full will be necessary for further considering the manuscript in our journal, and acceptance of the manuscript will entail a second round of review. EMBO Molecular Medicine encourages a single round of revision only and therefore, acceptance or rejection of the manuscript will depend on the completeness of your responses included in the next, final version of the manuscript. For this reason, and to save you from any frustrations in the end, I would strongly advise against returning an incomplete revision.

If you would like to discuss further the points raised by the referees, I am available to do so via email or video. Let me know if you are interested in this option.

We are expecting your revised manuscript within three months, if you anticipate any delay, please contact us.

When submitting your revised manuscript, please carefully review the instructions that follow below. We perform an initial quality control of all revised manuscripts before re-review; failure to include requested items will delay the evaluation of your revision.

We require:

1) A .docx formatted version of the manuscript text (including legends for main figures, EV figures and tables). Please make sure that the changes are highlighted to be clearly visible.

2) Individual production quality figure files as .eps, .tif, .jpg (one file per figure). For guidance, download the 'Figure Guide PDF' (https://www.embopress.org/page/journal/17574684/authorguide#figureformat).

3) At EMBO Press we ask authors to provide source data for the main figures. Our source data coordinator will contact you to discuss which figure panels we would need source data for and will also provide you with helpful tips on how to upload and organize the files.

4) A .docx formatted letter INCLUDING the reviewers' reports and your detailed point-by-point responses to their comments. As part of the EMBO Press transparent editorial process, the point-by-point response is part of the Review Process File (RPF), which will be published alongside your paper.

5) A complete author checklist, which you can download from our author guidelines (https://www.embopress.org/page/journal/17574684/authorguide#submissionofrevisions). Please insert information in the checklist that is also reflected in the manuscript. The completed author checklist will also be part of the RPF.

6) Please note that all corresponding authors are required to supply an ORCID ID for their name upon submission of a revised manuscript.

7) It is mandatory to include a 'Data Availability' section after the Materials and Methods. Before submitting your revision, primary datasets produced in this study need to be deposited in an appropriate public database, and the accession numbers and database listed under 'Data Availability'. Please remember to provide a reviewer password if the datasets are not yet public (see https://www.embopress.org/page/journal/17574684/authorguide#dataavailability).

In case you have no data that requires deposition in a public database, please state so in this section. Note that the Data Availability Section is restricted to new primary data that are part of this study.
This study includes no data deposited in external repositories.

8) For data quantification: please specify the name of the statistical test used to generate error bars and P values, the number (n) of independent experiments (specify technical or biological replicates) underlying each data point and the test used to calculate p-values in each figure legend. The figure legends should contain a basic description of n, P and the test applied. Graphs must include a description of the bars and the error bars (s.d., s.e.m.). Please provide exact p values.

9) Our journal encourages inclusion of *data citations in the reference list* to directly cite datasets that were re-used and obtained from public databases. Data citations in the article text are distinct from normal bibliographical citations and should directly link to the database records from which the data can be accessed. In the main text, data citations are formatted as follows: "Data ref: Smith et al, 2001" or "Data ref: NCBI Sequence Read Archive PRJNA342805, 2017". In the Reference list, data citations must be labeled with "[DATASET]". A data reference must provide the database name, accession number/identifiers and a resolvable link to the landing page from which the data can be accessed at the end of the reference. Further instructions are available at .

10) We replaced Supplementary Information with Expanded View (EV) Figures and Tables that are collapsible/expandable online. A maximum of 5 EV Figures can be typeset. EV Figures should be cited as 'Figure EV1, Figure EV2" etc... in the text and their respective legends should be included in the main text after the legends of regular figures.
- For the figures that you do NOT wish to display as Expanded View figures, they should be bundled together with their legends in a single PDF file called *Appendix*, which should start with a short Table of Content. Appendix figures should be referred to in the main text as: "Appendix Figure S1, Appendix Figure S2" etc.
- Additional Tables/Datasets should be labeled and referred to as Table EV1, Dataset EV1, etc. Legends have to be provided in a separate tab in case of .xls files. Alternatively, the legend can be supplied as a separate text file (README) and zipped together with the Table/Dataset file.
See detailed instructions here:

11) The paper explained: EMBO Molecular Medicine articles are accompanied by a summary of the articles to emphasize the major findings in the paper and their medical implications for the non-specialist reader. Please provide a draft summary of your article highlighting
- the medical issue you are addressing,
- the results obtained and
- their clinical impact.
This may be edited to ensure that readers understand the significance and context of the research. Please refer to any of our published articles for an example.

12) For more information: There is space at the end of each article to list relevant web links for further consultation by our readers. Could you identify some relevant ones and provide such information as well? Some examples are patient associations, relevant databases, OMIM/proteins/genes links, author's websites, etc...

13) Author contributions: CRediT has replaced the traditional author contributions section because it offers a systematic machine readable author contributions format that allows for more effective research assessment. Please remove the Authors Contributions from the manuscript and use the free text boxes beneath each contributing author's name in our system to add specific details on the author's contribution. More information is available in our guide to authors.

14) Disclosure statement and competing interests: We updated our journal's competing interests policy in January 2022 and request authors to consider both actual and perceived competing interests. Please review the policy https://www.embopress.org/competing-interests and update your competing interests if necessary.

15) Every published paper now includes a 'Synopsis' to further enhance discoverability. Synopses are displayed on the journal webpage and are freely accessible to all readers. They include a short stand first (maximum of 300 characters, including space) as well as 2-5 one-sentences bullet points that summarizes the paper. Please write the bullet points to summarize the key NEW findings. They should be designed to be complementary to the abstract - i.e. not repeat the same text. We encourage inclusion of key acronyms and quantitative information (maximum of 30 words / bullet point). Please use the passive voice. Please attach these in a separate file or send them by email, we will incorporate them accordingly.

Please also suggest a striking image or visual abstract to illustrate your article as a PNG file 550 px wide x 300-600 px high.
Share synopsis text and image, as well as eTOC:
Please note that these would be the final versions and changes during proofing are usually not allowed

16) As part of the EMBO Publications transparent editorial process initiative (see our Editorial at http://embomolmed.embopress.org/content/2/9/329), EMBO Molecular Medicine will publish online a Review Process File (RPF) to accompany accepted manuscripts.
In the event of acceptance, this file will be published in conjunction with your paper and will include the anonymous referee reports, your point-by-point response and all pertinent correspondence relating to the manuscript. Let us know whether you agree with the publication of the RPF and as here, if you want to remove or not any figures from it prior to publication.
Please note that the Authors checklist will be published at the end of the RPF.

EMBO Molecular Medicine has a "scooping protection" policy, whereby similar findings that are published by others during

review or revision are not a criterion for rejection. Should you decide to submit a revised version, I do ask that you get in touch after three months if you have not completed it, to update us on the status.

I look forward to receiving your revised manuscript.

Yours sincerely,

Poonam Bheda

Poonam Bheda, PhD
Scientific Editor
EMBO Molecular Medicine

***** Reviewer's comments *****

Referee #1 (Comments on Novelty/Model System for Author):

see review

Referee #1 (Remarks for Author):

This revised manuscript reports that antibody targeting in pcmt1 deficient mice reduces inflammation and doubles lifespan. Unfortunately, many of the claims relate to highly complicated systems that are not adequately disentangled to support the conclusions. For example, the authors refer to isoAsp, which is an unfortunately vague term. Deamidation generates four isomeric forms of L-Asn, L/D-Asp and L/D-betaAsp. Presumably the authors refer to L-betaAsp as the 'isoAsp' as it is the major form, but this issue is not addressed here or in their previous papers. In relation to this, there is no description of how the 'isoDGR' antibody was developed. I found no description of its validation or methods for its creation in the previous literature either. It is therefore quite unclear what exactly this antibody targets. In much of the data in the present work, the same antibody is used for both detection and as the removal agent. Such experiments offer very little information. If the antibody is used to lower 'isoDGR', a different method should be used to evaluate the extent to which isoDGR levels were affected, since presumable the antibody recognizes its target (although we don't know what that target is). Similarly, the pcmt1 deficiency leads to a great number of pathological complications, which may be interrelated, and make drawing independent conclusions very difficult. Doubling the lifespan is very interesting, but what exactly is the origin of that? It may have very little to do with reduced inflammation, but rather extension of neuronal integrity or many other possibilities. The answers likely are not to be had by wholescale examination of tissues by IF. There is a similar lack of attention to detail in many aspects of the work. For example, proteins were incubated at pH 9 for deamidation. This could also lead to truncation, aggregation, or other modifications. These issues were not addressed. Given all of the uncertainties associated with this entire system, the conclusions are simply not supported.

Referee #2 (Remarks for Author):

The authors have made a significant effort to address all my criticisms, especially those regarding the necessity of testing the effect of an IgG antibody in vivo. I realize it was a significant amount of work but I do think that was essential to strengthen the conclusions of the manuscript. As such, I would like to thank the authors for their effort.
All other comments I raised, including testing the effect of the antibody in naturally aged mice, have also been upgraded in this new version. Therefore, I only have a very minor comment before fully endorsing the publication of this manuscript.

In response to my comment #6 the authors replied:

Answer: We apologize if we were not sufficiently clear when explaining our approach to quantifying immunofluorescence images. The readings were acquired by Image J software using images from n=5 mice. The software randomly samples 50 readings in different locations per image. This is a commonly used method of quantifying immunofluorescent signals. We actually compared the average of all counted fields for every animal (n=5). Most published papers use n=5 animals for this type of approach (similar to our own strategy) then 8 report average fluorescent intensity. In the revised manuscript, all analyses of this type report data from at least n=5 mice.

Could the authors include the bellow sentence (extracted for their response) in the materials and methods section?:
"The readings were acquired by Image J software using images from n=5 mice. The software randomly samples 50 readings in

different locations per image."

Response to Reviewers' Comments:
We would like to thank the editor and reviewers for providing comments and feedback to improve our manuscript. We have carefully addressed each point as detailed below in blue font. Individual changes have also been highlighted in the revised manuscript.

Referee #1 (Remarks for Author):

This revised manuscript reports that antibody targeting in pcmt1 deficient mice reduces inflammation and doubles lifespan. Unfortunately, many of the claims relate to highly complicated systems that are not adequately disentangled to support the conclusions. For example, the authors refer to isoAsp, which is an unfortunately vague term. Deamidation generates four isomeric forms of L-Asn, L/D-Asp and L/D-betaAsp. Presumably the authors refer to L-betaAsp as the 'isoAsp' as it is the major form, but this issue is not addressed here or in their previous papers.

Answer: We appreciate the reviewer's questions and apologize for any confusion. Due to space constraints, we are unable to provide a detailed account of our decade-long research that forms the foundation of this manuscript. Our long-term commitment is to explore the impact of protein aging, specifically protein deamidation, on human health and disease. To detect naturally occurring protein deamidations in human tissues, we have developed a range of proteomics sample preparation and LC-MS/MS proteomic methods designed to prevent artificial deamidation during sample processing. These methods have allowed us to accurately identify endogenous deamidation products, as documented in the following papers.

Hao, P., Ren, Y., Alpert, A. J. & Sze, S. K. Detection, evaluation and minimization of nonenzymatic deamidation in proteomic sample preparation. Molecular & cellular proteomics : MCP 10, O111.009381, doi:10.1074/mcp.O111.009381 (2011).

Hao, P., Ren, Y., Datta, A., Tam, J. P. & Sze, S. K. Evaluation of the effect of trypsin digestion buffers on artificial deamidation. Journal of proteome research 14, 1308-1314, doi:10.1021/pr500903b (2015).

To comprehensively characterize the deamidation products, we have enhanced a mixed-mode chromatography method. This improvement involves utilizing ion-exchange chromatography in HILIC (Hydrophilic Interaction Liquid Chromatography) mode to separate the trio of deamidation products, which includes peptides containing Asn, L/D-Asp, and L/D-betaAsp (L/D-isoAsp) as reported in

Hao, P. et al. Enhanced separation and characterization of deamidated peptides with RP-ERLIC-based multidimensional chromatography coupled with tandem mass spectrometry. Journal of proteome research 11, 1804-1811, doi:10.1021/pr201048c (2012).

Serra, A., Gallart-Palau, X., Wei, J. & Sze, S. K. Characterization of Glutamine Deamidation by Long-Length Electrostatic Repulsion-Hydrophilic Interaction Chromatography-Tandem Mass Spectrometry (LERLIC-MS/MS) in Shotgun Proteomics. Analytical chemistry 88, 10573-10582, doi:10.1021/acs.analchem.6b02688 (2016).

By employing the advanced LC-MS/MS-based proteomics methods to analyze human aortic tissues and atherosclerotic plaque samples, we successfully identified numerous protein deamidations within the patients' tissues. The comprehensive nature of our deamidation identification enabled us to conduct a motif analysis, revealing the accumulation of

deamidation products associated with the NGR sequence motif in the patients' tissues. These findings were presented at the 23rd American Peptide Symposium.

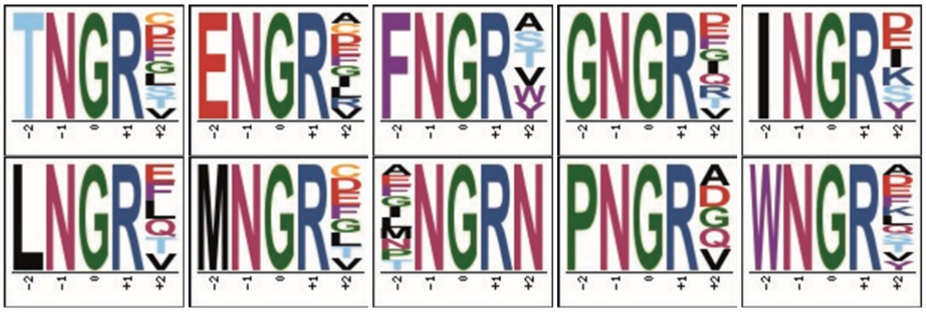

Fig. 2. Some of the NGR containing motifs identified in the deamidated peptides in the cardiovascular system by motif-x program.

Cheow, E. S. H. et al. The Role of Protein Deamidation in Cardiovascular Disease. Proceedings of the 23rd American Peptide Symposium 17, 212-213 (2013).

Through a literature search, we discovered that the deamidation product of NGR, known as isoDGR, has been previously identified as binding to integrin receptors through phage display experiments. This interaction between isoDGR and integrin was subsequently validated through various methods, including X-ray crystallography and biochemical assays as reported in the following papers.

Curnis, F. et al. Spontaneous Formation of L-Isoaspartate and Gain of Function in Fibronectin. Journal of Biological Chemistry 281, 36466-36476, doi:10.1074/jbc.M604812200 (2006).

Spitaleri, A. et al. Structural basis for the interaction of isoDGR with the RGD-binding site of alphavbeta3 integrin. The Journal of biological chemistry 283, 19757-19768, doi:10.1074/jbc.M710273200 (2008).

Corti, A. & Curnis, F. Isoaspartate-dependent molecular switches for integrin-ligand recognition. Journal of cell science 124, 515-522, doi:10.1242/jcs.077172 (2011).

Consequently, we undertook a detailed investigation into the accumulation of isoDGR in human atherosclerotic plaques. Our research revealed that several long-lived extracellular matrix (ECM) proteins, including Fibronectin, Laminin, Tenascin, and others, displayed extensive isoDGR accumulation within patient tissues. To confirm that these isoDGR-modified proteins interacted with integrins expressed in immune cells, we observed that these modified proteins indeed bound to integrins found in monocyte-macrophages. To provide further evidence, we isolated peripheral blood mononuclear cells (PBMCs) from human blood and demonstrated the binding of isoDGR-modified proteins to human immune cells. Notably, these interactions were completely inhibited by integrin inhibitors and integrin-specific antibodies. These findings strongly suggest that ECM proteins damaged due to aging, bearing the isoDGR motif, play a role in binding to and recruiting immune cells, potentially representing one of the pathological mechanisms underlying atherosclerosis. The results were reported in

Dutta, B. et al. Monocyte adhesion to atherosclerotic matrix proteins is enhanced by Asn-Gly-Arg deamidation. Scientific reports 7, 5765, doi:10.1038/s41598-017-06202-2 (2017).

To confirm the role of isoDGR in cardiovascular diseases, we developed isoDGR-specific monoclonal antibodies (mAbs) through a collaboration with GeneScript, a research contract organization. We used (L-isoD)GR-containing peptides to immunize Balb/c mice and collected spleens for isolating spleenocytes. These cells were fused with myeloma cells to create 10 clones of hybridoma cells secreting isoDGR monoclonal antibodies. After rigorous screening and biochemical assays, we identified two clones, 6E11 and 14G7, with high specificity and affinity for isoDGR peptides and isoDGR-modified proteins. Then, we scaled up the production of these mAbs for immunoassays, functional studies of isoDGR-modified proteins, and potential immunotherapy in mouse models.

Both mAbs exhibited high specificity for isoDGR-modified peptides/proteins when tested against synthetic peptides containing isoDGR, as opposed to control NGR, DGR, and RGD motifs in ELISA assays and immunofluorescence imaging (please see results and figures in *Atherosclerosis 324, 58-68, doi:10.1016/j.atherosclerosis.2021.03.020, 2021*).

we further assessed the avidity of these two mAbs to isoDGR peptides using the surface plasmon resonance (SPR) method. In these assays, 14G7 exhibited a 1000-fold higher binding activity compared to 6E11, by using a range of isoDGR peptides, including L-isoDGR, acetylated-L-IsoDGR, and biotin-L-IsoDGR, as well as DGR, NGR, and RDG controls. Our findings showed that 14G7 strongly bound to all isoDGR-peptides but did not interact with DGR, NGR, or RGD peptides. Conversely, 6E11 exhibited weaker binding to all isoDGR-modified molecules and did not display non-specific binding to DGR, NGR, or RGD.

In the report, we also discovered that isoDGR peptides bind to integrins on macrophages, leading to the stimulation of macrophages to secrete proinflammatory cytokines, as assessed using the LEGENDplex™ multiplex mouse cytokines assay kits (Biolegend, San Diego, CA).

Building on the foundation work described above, in this manuscript we reported the immunotherapy targeting isoDGR-damaged proteins extended the lifespan in Pcmt1-/- mice. When we applied these mAbs in immunotherapy experiments on Pcmt1-/- mice, we observed that 14G7 significantly extended the lifespan of the mice. These results indicate that our 14G7 monoclonal antibody treatment effectively and selectively induced the immune clearance of isoDGR-modified protein accumulation in blood vessels and various tissues, while also reducing pro-inflammatory cytokines in naturally aging and Pcmt1-/- mice.

In relation to this, there is no description of how the 'isoDGR' antibody was developed. I found no description of its validation or methods for its creation in the previous literature either. It is therefore quite unclear what exactly this antibody targets.

Answer: On page 14 of the manuscript, we specified that "The monoclonal antibody specifically developed to target (L-isoD)GR motif had been previously reported.(reference to Atherosclerosis 324, 58-68) Pcmt1$^{-/-}$ pups were intraperitoneally (i.p.) injected with 1mg/kg/week isoDGR-specific mAb starting from 1 week old until use in the study." The development of isoDGR antibodies has been documented in the supplementary methods of Atherosclerosis 324, 58-68. We regret any inconvenience caused to the reviewer, and we now provide a reproduction of the relevant description for clarity:

Generation of monoclonal antibody against isoDGR motif

Hybridoma cell line that secretes isoDGR-specific monoclonal antibody motif was generated by GenScript Corporation (Piscataway, NJ, USA). Two synthetic peptides, C(isoD)GRCK and (isoD)GR conjugated to keyhole limpet hemocyanin (KLH) were used to immunize Balb/c mice. Positive clones were selected by a series of dot blot and ELISA screenings against the isoDGR-containing antigens. We then used a combination of peptide-based ELISA and dot blot assays to test reactivity of the selected hybridoma cells against four peptides containing either isoDGR or the dummy sequences DGR, RGD, and NGR. isoDGR-specific antibodies in hybridoma culture supernatants were determined by indirect ELISA (Fig. 1a). Briefly, Nunc Maxisorp 96 well plates were coated overnight at 4°C with 100 μL/well of each of the peptides (2 μg/mL), and blocked with 5% BSA. The culture supernatants (1:500) were added and incubated at 37°C for 2 hr before adding the secondary antibody. The isoDGR motifs were detected by using horseradish peroxidase (HRP) goat anti-mouse antibodies (R&D systems, Minneapolis, USA) and TMB as a substrate. Absorbance was read at 450nm. Based on the results of these assays, we selected a single hybridoma that displayed high specificity for the isoDGR motif but lacked reactivity to DGR, RGD, or NGR (Fig. 1a). Monoclonal antibody was purified from the supernatant of hybridoma cell cultures using protein A/G agarose columns according to the manufacturer's instructions (Thermo Fisher Scientific, San Jose, USA).

In much of the data in the present work, the same antibody is used for both detection and as the removal agent. Such experiments offer very little information. If the antibody is used to lower 'isoDGR', a different method should be used to evaluate the extent to which isoDGR levels were affected, since presumable the antibody recognizes its target (although we don't know what that target is).

Answer: As described in the Atherosclerosis 324, 58-68 report, in addition to the various immunoassays and immunofluorescence imaging and other assays, we also used ERLIC-MS/MS proteomics methods to confirm the presence of isoDGR motif in target proteins. Multiple biophysical and biochemical methods have been used to firmly confirm the specificity of the mAbs to isoDGR-modified proteins. The 14G7 monoclonal antibody has demonstrated exceptional specificity, with a strong binding affinity KD = $3.4X10^{-9}$, toward (L-isoD)GR-modified proteins as determined using SPR method.

Similarly, the pcmt1 deficiency leads to a great number of pathological complications, which may be interrelated, and make drawing independent conclusions very difficult. Doubling the lifespan is very interesting, but what exactly is the origin of that? It may have very little to do with reduced inflammation, but rather extension of neuronal integrity or many other possibilities. The answers likely are not to be had by wholescale examination of tissues by IF.

Answer: As previously mentioned, the isoDGR motif's ability to bind to integrins has been well-established through phage display experiments and various biophysical and biochemical methods, both by our research team and others. Furthermore, our research has revealed that the isoDGR motif interacts with integrins on macrophages, leading to the initiation of proinflammatory signaling pathways, resulting in the secretion of pro-inflammatory cytokines and the induction of chronic inflammation. This manuscript aligns with previous findings, demonstrating a significant increase in inflammatory cytokines in Pcmt1-/- mice bearing the accumulation of isoDGR motif. Importantly, we have shown that this effect can be reversed through the injection of therapeutic isoDGR-specific monoclonal antibodies.

In summary, the cumulative evidence strongly supports the notion that immune clearance of isoDGR-modified proteins extends the lifespan of Pcmt1 mice by mitigating chronic inflammation. It is well-demonstrated that isoDGR-modified proteins bind to integrin receptors, triggering the secretion of pro-inflammatory cytokines and initiating systemic chronic inflammation, which leads to a complex pathology affecting multiple organs. Immunotherapeutic targeting of isoDGR-modified proteins with specific monoclonal antibodies promotes the immune clearance of damaged isoDGR proteins in vivo and, consequently, extends the lifespan of Pcmt1-/- mice.

There is a similar lack of attention to detail in many aspects of the work. For example, proteins were incubated at pH 9 for deamidation. This could also lead to truncation, aggregation, or other modifications. These issues were not addressed. Given all of the uncertainties associated with this entire system, the conclusions are simply not supported.

Answer: The induction of protein deamidation under alkaline conditions is a widely recognized mechanism. All of our in-vitro experiments for preparing isoDGR-modified proteins were conducted under mild alkaline condition. To thoroughly assess their modifications, truncations, and aggregation, we conducted comprehensive analyses using both mass spectrometry, SDS-PAGE and WB analysis. In fact, as detailed in our last response to the reviewer's comments, we clearly demonstrated that this condition generates isoDGR-modified proteins without inducing truncation, aggregation, or other modifications. This was evidenced by Ponceau S staining as showed in the images below. In addition, it has been confirmed by mass spectrometry analysis, as reported in our studies published in Scientific Reports 7, 5765 and Atherosclerosis 324, 58-68.

Fibronectin aging in 100 mM $NaHCO_3$ pH 8.5

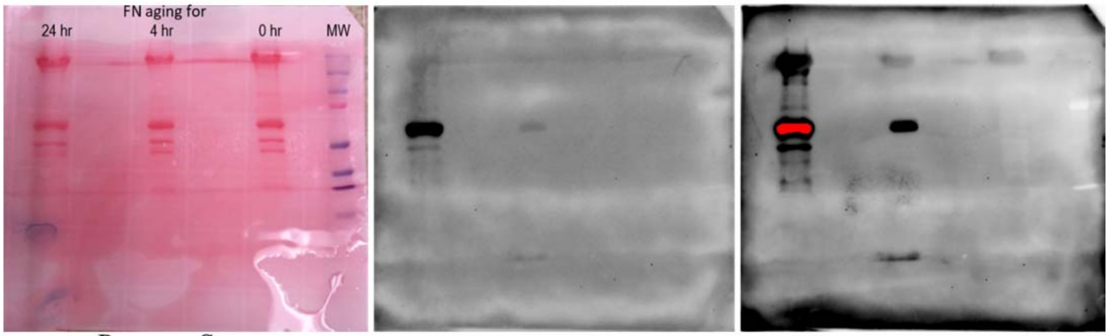

Ponceau S WB 1 min WB 5 min

We are grateful for the reviewer's feedback, and we have taken the opportunity to include a comprehensive narrative description of the foundational work in this response. We trust that this detailed account of our research history will serve to clarify any confusion experienced by the reviewer.

Referee #2 (Remarks for Author):

The authors have made a significant effort to address all my criticisms, especially those regarding the necessity of testing the effect of an IgG antibody in vivo. I realize it was a significant amount of work but I do think that was essential to strengthen the conclusions of

the manuscript. As such, I would like to thank the authors for their effort.
All other comments I raised, including testing the effect of the antibody in naturally aged mice, have also been upgraded in this new version. Therefore, I only have a very minor comment before fully endorsing the publication of this manuscript.

In response to my comment #6 the authors replied:

Answer: We apologize if we were not sufficiently clear when explaining our approach to quantifying immunofluorescence images. The readings were acquired by Image J software using images from n=5 mice. The software randomly samples 50 readings in different locations per image. This is a commonly used method of quantifying immunofluorescent signals. We actually compared the average of all counted fields for every animal (n=5). Most published papers use n=5 animals for this type of approach (similar to our own strategy) then 8 report average fluorescent intensity. In the revised manuscript, all analyses of this type report data from at least n=5 mice.

Could the authors include the bellow sentence (extracted for their response) in the materials and methods section?:
"The readings were acquired by Image J software using images from n=5 mice. The software randomly samples 50 readings in different locations per image."

Answer: We appreciate the positive feedback from the reviewers. In accordance with the reviewer's suggestion, we have included the sentence “The readings were acquired using Image J software, utilizing images from n=5 mice. The software randomly sampled 50 readings from different locations per image.” in the materials and methods section as per the reviewer's recommendation.

18th Oct 2023

Dear Prof. Sze,

Thank you for the submission of your revised manuscript to EMBO Molecular Medicine. We have now received the enclosed report from referee 1 that was asked to re-assess it. I am pleased to inform you that we will be able to accept your manuscript pending the following final amendments:

1) Please check "Author Checklist" carefully and complete all relevant questions. Currently information about XX is missing (studies involving experimental animals is missing).
2) Source Data: Please ensure that a completed Source Data checklist is uploaded, along with a single source data file (zipped) per figure, with the panels clearly visible in the folder structure. There seems to have been a mislabeling for Fig 1A/Fig3A/Fig5A, please check. Source Data for Fig 5E also seems to be missing.
3) In the main manuscript file, please do the following:
- Reduce keywords to max. 5.
- Data availability: Please move the Data Availability section to the end of the Materials and Methods section
- Please note that funding information should be given in the "Acknowledgements" section (not in its own separate section).
- Please rename "Competing Interest" to "Disclosure and competing interests statement". We updated our journal's competing interests policy in January 2022 and request authors to consider both actual and perceived competing interests. Please review the policy https://www.embopress.org/competing-interests and update your competing interests if necessary.
- Author contributions: Please remove it from the manuscript and specify author contributions in our submission system. CRediT has replaced the traditional author contributions section because it offers a systematic machine-readable author contributions format that allows for more effective research assessment. You are encouraged to use the free text boxes beneath each contributing author's name to add specific details on the author's contribution. More information is available in our guide to authors: https://www.embopress.org/page/journal/17574684/authorguide#authorshipguidelines
- Please correct the reference citation in the reference list. Where there are more than 10 authors on a paper, note that only 10 will be listed, followed by "et al.". Please check "Author Guidelines" for more information. https://www.embopress.org/page/journal/17574684/authorguide#referencesformat
- Data not shown: We do not allow statements/conclusions with "data not shown". As per our guidelines, on "Unpublished Data" the journal does not permit citation of "Data not shown". All data referred to in the paper should be displayed in the main or Expanded View figures. Please remove from pages 5 and 10.
4) Please place individual sections of the manuscript in the following order: Title page - Abstract & Keywords - Introduction - Results - Discussion - Materials & Methods - Data Availability - Acknowledgements - Disclosure and Competing Interests Statement - References - Figure Legends - Expanded View Figure Legends.
5) For the figures and figure legends, please take care of the following:
- Please make sure to update the callouts of all figures in the main manuscript text (currently figure callouts are missing for Figure 4B, Figure 7 is called out before Figure 5. Panel callouts are missing for Figure 7 and Appendix Figure S5).
- Please note that the figure legend style does not comply with the journal guidelines i.e. all the figure legends are in a run-on style.
- Please note that a separate 'Data Information' section is required in the legends of all figures.
- Please note that information related to n is missing in the legend of Figure 3D, 5D, and 5E.
- Please note that we require exact p-values to be reported. Currently exact p-values are not provided.
- Please ensure that data points are defined as technical or biological replicates. Currently it is unclear in Figure 2c and 2d whether the individual points represent individual mice, or biological or technical replicates of mixed samples.
6) In the Materials and Methods, please take care of the following:
- Cell lines: Please include all information requested in the author checklist for cell lines used in the manuscript including accession number in repository or supplier name, catalog number, clone number, and/or RRID). Please also be sure to include a sentence in the Materials and Methods as to whether or not the cell lines were recently authenticated and tested for mycoplasma contamination.
- Please ensure that a statement on whether or not blinding was done is included in the Materials and Methods even if no blinding was done.
7) The Paper Explained: Please edit the claim on doubling lifespan (as was previously suggested for the title) to more simply extending the lifespan.
8) Synopsis: Please check your synopsis text and image before submission with your revised manuscript. Please be aware that in the proof stage minor corrections only are allowed (e.g., typos).
- Synopsis image: Please remove it from the manuscript and upload it as a high-resolution jpeg file 550 pixels wide x (250-400) pixels high. Please also remove the legend. Please also simplify the synopsis image. Currently the p-value and additional text will be difficult to read in a small format. Moreover, we would advise a graphic that summarises the main findings of the manuscript on a glance.
- Synopsis text: Please remove the synopsis from the main manuscript file and upload it as a separate file. Please also modify the text in point 3 (as was previously suggested for the title) to more simply extending the lifespan in Pcmt1 KO mice (as opposed to "doubles lifespan").

9) For more information: This space should be used to list relevant web links for further consultation by our readers. Could you identify some relevant ones and provide such information as well? Some examples are patient associations, relevant databases, OMIM/proteins/genes links, author's websites, etc...
10) Appendix file: Please upload the Appendix as a single PDF (no separate image files are needed) and add page numbers to the Table of Contents.
11) As part of the EMBO Publications transparent editorial process initiative (see our Editorial at http://embomolmed.embopress.org/content/2/9/329), EMBO Molecular Medicine will publish online a Review Process File (RPF) to accompany accepted manuscripts. This file will be published in conjunction with your paper and will include the anonymous referee reports, your point-by-point response and all pertinent correspondence relating to the manuscript. Let us know whether you agree with the publication of the RPF and as here, if you want to remove or not any figures from it prior to publication. Please note that the Authors checklist will be published at the end of the RPF.
12) Please provide a point-by-point letter INCLUDING my comments as well as the reviewer's reports and your detailed responses (as Word file).

I look forward to reading a new revised version of your manuscript as soon as possible.

Yours sincerely,

Poonam Bheda

Poonam Bheda, PhD
Scientific Editor
EMBO Molecular Medicine

***** Reviewer's comments *****

Referee #1 (Comments on Novelty/Model System for Author):

noted previously

Referee #1 (Remarks for Author):

The authors have provided adequate responses and the framing in the title and elsewhere are a better match for the results. The information about the antibody validation that is provided in the response is not provided in the citation that they list. That information should be included in the present manuscript as it is crucial for anyone to reproduce the results.

Referee #1 (Comments on Novelty/Model System for Author):

noted previously

Referee #1 (Remarks for Author):

The authors have provided adequate responses and the framing in the title and elsewhere are a better match for the results. The information about the antibody validation that is provided in the response is not provided in the citation that they list. That information should be included in the present manuscript as it is crucial for anyone to reproduce the results.

Response: We appreciate the reviewer's feedback. The validation of the antibody has been previously documented in Atherosclerosis (2021) with the publication details available under doi:10.1016/j.atherosclerosis.2021.03.020. In this publication, Figure 1A and Supplementary Figure S1 illustrate the validation method and binding specificity of the isoDGR-specific monoclonal antibody. Additionally, the Supplementary Methods section, titled "Generation of Monoclonal Antibody against isoDGR Motifs," provides a detailed account of the antibody generation process.

31st Oct 2023

Dear Prof. Sze,

Please find enclosed the final reports on your manuscript. We are pleased to inform you that your manuscript is accepted for publication and is now being sent to our publisher to be included in the next available issue of EMBO Molecular Medicine.

Please read below for additional IMPORTANT information regarding your article, its publication and the production process.

Congratulations on your interesting work,

Poonam Bheda

Poonam Bheda, PhD
Scientific Editor
EMBO Molecular Medicine

Follow us on Twitter @EmboMolMed
Sign up for eTOCs at embopress.org/alertsfeeds

*** *** *** IMPORTANT INFORMATION *** *** ***

SPEED OF PUBLICATION
The journal aims for rapid publication of papers, using using the advance online publication "Early View" to expedite the process: A properly copy-edited and formatted version will be published as "Early View" after the proofs have been corrected. Please help the Editors and publisher avoid delays by providing e-mail address(es), telephone and fax numbers at which author(s) can be contacted.

Should you be planning a Press Release on your article, please get in contact with embomolmed@wiley.com as early as possible, in order to coordinate publication and release dates.

LICENSE AND PAYMENT:

All articles published in EMBO Molecular Medicine are fully open access: immediately and freely available to read, download and share.

EMBO Molecular Medicine charges an article processing charge (APC) to cover the publication costs. You, as the corresponding author for this manuscript, should have already received a quote with the article processing fee separately. Please let us know in case this quote has not been received.

Once your article is at Wiley for editorial production you will receive an email from Wiley's Author Services system, which will ask you to log in and will present you with the publication license form for completion. Within the same system the publication fee can be paid by credit card, an invoice, pro forma invoice or purchase order can be requested.

Payment of the publication charge and the signed Open Access Agreement form must be received before the article can be published online.

PROOFS

You will receive the proofs by e-mail approximately 2 weeks after all relevant files have been sent o our Production Office. Please return them within 48 hours and if there should be any problems, please contact the production office at embopressproduction@wiley.com.

Please inform us if there is likely to be any difficulty in reaching you at the above address at that time. Failure to meet our deadlines may result in a delay of publication.

All further communications concerning your paper proofs should quote reference number EMM-2023-18526-V3 and be directed to the production office at embopressproduction@wiley.com.

Thank you,

Poonam Bheda, PhD
Scientific Editor
EMBO Molecular Medicine

EMBO Press Author Checklist

Corresponding Author Name: Siu Kwan Sze	
Journal Submitted to: EMBO Molecular Medicine	
Manuscript Number: EMM-2023-18526	

USEFUL LINKS FOR COMPLETING THIS FORM
The EMBO Journal - Author Guidelines
EMBO Reports - Author Guidelines
Molecular Systems Biology - Author Guidelines
EMBO Molecular Medicine - Author Guidelines

Reporting Checklist for Life Science Articles (updated January
This checklist is adapted from Materials Design Analysis Reporting (MDAR) Checklist for Authors. MDAR establishes a minimum set of requirements in transparent reporting in the life sciences (see Statement of Task: 10.31222/osf.io/9sm4x). Please follow the journal's guidelines in preparing your
Please note that a copy of this checklist will be published alongside your article.

Abridged guidelines for figures
1. Data
The data shown in figures should satisfy the following conditions:
- the data were obtained and processed according to the field's best practice and are presented to reflect the results of the experiments in an accurate and unbiased manner.
- ideally, figure panels should include only measurements that are directly comparable to each other and obtained with the same assay.
- plots include clearly labeled error bars for independent experiments and sample sizes. Unless justified, error bars should not be shown for technical
- if n<5, the individual data points from each experiment should be plotted. Any statistical test employed should be justified.
- Source Data should be included to report the data underlying figures according to the guidelines set out in the authorship guidelines on Data

2. Captions
Each figure caption should contain the following information, for each panel where they are relevant:
- a specification of the experimental system investigated (eg cell line, species name).
- the assay(s) and method(s) used to carry out the reported observations and measurements.
- an explicit mention of the biological and chemical entity(ies) that are being measured.
- an explicit mention of the biological and chemical entity(ies) that are altered/varied/perturbed in a controlled manner.
- the exact sample size (n) for each experimental group/condition, given as a number, not a range;
- a description of the sample collection allowing the reader to understand whether the samples represent technical or biological replicates (including how many animals, litters, cultures, etc.).
- a statement of how many times the experiment shown was independently replicated in the laboratory.
- definitions of statistical methods and measures:
  - common tests, such as t-test (please specify whether paired vs. unpaired), simple χ2 tests, Wilcoxon and Mann-Whitney tests, can be unambiguously identified by name only, but more complex techniques should be described in the methods section;
  - are tests one-sided or two-sided?
  - are there adjustments for multiple comparisons?
  - exact statistical test results, e.g., P values = x but not P values < x;
  - definition of 'center values' as median or average;
  - definition of error bars as s.d. or s.e.m.

Please complete ALL of the questions below.
Select "Not Applicable" only when the requested information is not relevant for your study.

Materials

Newly Created Materials	Information included in the manuscript?	In which section is the information available? (Reagents and Tools Table, Materials and Methods, Figures, Data Availability Section)
New materials and reagents need to be available; do any restrictions apply?	Not Applicable	

Antibodies	Information included in the manuscript?	In which section is the information available? (Reagents and Tools Table, Materials and Methods, Figures, Data Availability Section)
For **antibodies** provide the following information:<br>- Commercial antibodies: RRID (if possible) or supplier name, catalogue number and or/clone number<br>- Non-commercial: RRID or citation	Yes	Materials and Methods

DNA and RNA sequences	Information included in the manuscript?	In which section is the information available? (Reagents and Tools Table, Materials and Methods, Figures, Data Availability Section)
**Short novel DNA or RNA including primers, probes**: provide the sequences.	Yes	Supplementary Tables S1 and S2

Cell materials	Information included in the manuscript?	In which section is the information available? (Reagents and Tools Table, Materials and Methods, Figures, Data Availability Section)
**Cell lines:** Provide species information, strain. Provide accession number in repository **OR** supplier name, catalog number, clone number, and/**OR** RRID.	Yes	Materials and Methods
**Primary cultures:** Provide species, strain, sex of origin, genetic modification status.	Not Applicable	
Report if the cell lines were recently **authenticated** (e.g., by STR profiling) and tested for mycoplasma contamination.	Yes	Materials and Methods

Experimental animals	Information included in the manuscript?	In which section is the information available? (Reagents and Tools Table, Materials and Methods, Figures, Data Availability Section)
**Laboratory animals or Model organisms:** Provide species, strain, sex, age, genetic modification status. Provide accession number in repository **OR** supplier name, catalog number, clone number, **OR** RRID.	Yes	Materials and Methods
**Animal observed in or captured from the field:** Provide species, sex, and age where possible.	Not Applicable	
Please detail **housing and husbandry conditions**.	Yes	Materials and Methods

Plants and microbes	Information included in the manuscript?	In which section is the information available? (Reagents and Tools Table, Materials and Methods, Figures, Data Availability Section)
**Plants:** provide species and strain, ecotype and cultivar where relevant, unique accession number if available, and source (including location for collected wild specimens).	Not Applicable	
**Microbes:** provide species and strain, unique accession number if available, and source.	Not Applicable	

Human research participants	Information included in the manuscript?	In which section is the information available? (Reagents and Tools Table, Materials and Methods, Figures, Data Availability Section)
If collected and within the bounds of privacy constraints report on age, sex and gender or ethnicity for all study participants.	Not Applicable	

Core facilities	Information included in the manuscript?	In which section is the information available? (Reagents and Tools Table, Materials and Methods, Figures, Data Availability Section)
If your work benefited from core facilities, was their service mentioned in the acknowledgments section?	Not Applicable	

Design

Study protocol	Information included in the manuscript?	In which section is the information available? (Reagents and Tools Table, Materials and Methods, Figures, Data Availability Section)
If study protocol has been **pre-registered, provide DOI in the manuscript**. For clinical trials, provide the trial registration number **OR** cite DOI.	Not Applicable	
Report the **clinical trial registration number** (at ClinicalTrials.gov or equivalent), where applicable.	Not Applicable	

Laboratory protocol	Information included in the manuscript?	In which section is the information available? (Reagents and Tools Table, Materials and Methods, Figures, Data Availability Section)
Provide DOI OR other citation details if **external detailed step-by-step protocols** are available.	Not Applicable	

Experimental study design and statistics	Information included in the manuscript?	In which section is the information available? (Reagents and Tools Table, Materials and Methods, Figures, Data Availability Section)
Include a statement about **sample size** estimate even if no statistical methods were used.	Yes	Materials and Methods, Figures
Were any steps taken to minimize the effects of subjective bias when allocating animals/samples to treatment (e.g. **randomization procedure**)? If yes, have they been described?	Yes	Materials and Methods
Include a statement about **blinding** even if no blinding was done.	Not Applicable	Materials and Methods
Describe **inclusion/exclusion criteria** if samples or animals were excluded from the analysis. Were the criteria pre-established?<br><br>If sample or data points were omitted from analysis, report if this was due to attrition or intentional exclusion and provide justification.	Not Applicable	
For every figure, are **statistical tests** justified as appropriate? Do the data meet the assumptions of the tests (e.g., normal distribution)? Describe any methods used to assess it. Is there an estimate of variation within each group of data? Is the variance similar between the groups that are being statistically compared?	Yes	Materials and Methods, Figures

Sample definition and in-laboratory replication	Information included in the manuscript?	In which section is the information available? (Reagents and Tools Table, Materials and Methods, Figures, Data Availability Section)
In the figure legends: state number of times the experiment was **replicated** in laboratory.	Yes	Materials and Methods, Figures
In the figure legends: define whether data describe **technical or biological replicates**.	Yes	Materials and Methods, Figures

Ethics

Ethics	Information included in the manuscript?	In which section is the information available? (Reagents and Tools Table, Materials and Methods, Figures, Data Availability Section)
Studies involving **human participants**: State details of **authority granting ethics approval** (IRB or equivalent committee(s), provide reference number for approval.	Not Applicable	
Studies involving **human participants**: Include a statement confirming that **informed consent** was obtained from all subjects and that the experiments conformed to the principles set out in the WMA Declaration of Helsinki and the Department of Health and Human Services Belmont Report.	Not Applicable	
Studies involving **human participants: For** publication of **patient photos**, include a statement confirming that consent to publish was obtained.	Not Applicable	
Studies involving experimental **animals**: State details of **authority granting ethics approval** (IRB or equivalent committee(s), provide reference number for approval. Include a statement of compliance with ethical regulations.	Yes	Materials and Methods
Studies involving **specimen and field samples:** State if relevant **permits** obtained, provide details of authority approving study; if none were required, explain why.	Not Applicable	

Dual Use Research of Concern (DURC)	Information included in the manuscript?	In which section is the information available? (Reagents and Tools Table, Materials and Methods, Figures, Data Availability Section)
Could your study fall under dual use research restrictions? Please check biosecurity documents and list of **select agents and toxins** (CDC): https://www.selectagents.gov/sat/list.htm	Not Applicable	
If you used a select agent, is the security level of the lab appropriate and reported in the manuscript?	Not Applicable	
If a study is subject to dual use research of concern regulations, is the name of the **authority granting approval and reference number** for the regulatory approval provided in the manuscript?	Not Applicable	

Reporting

The MDAR framework recommends adoption of discipline-specific guidelines, established and endorsed through community initiatives. Journals have their own policy about requiring specific guidelines and recommendations to complement MDAR.

Adherence to community standards	Information included in the manuscript?	In which section is the information available? (Reagents and Tools Table, Materials and Methods, Figures, Data Availability Section)
State if relevant guidelines or checklists (e.g., **ICMJE, MIBBI, ARRIVE, PRISMA**) have been followed or provided.	Not Applicable	
For **tumor marker prognostic studies,** we recommend that you follow the **REMARK** reporting guidelines (see link list at top right). See author guidelines, under 'Reporting Guidelines'. Please confirm you have followed these guidelines.	Not Applicable	
For **phase II and III randomized controlled trials**, please refer to the **CONSORT** flow diagram (see link list at top right) and submit the CONSORT checklist (see link list at top right) with your submission. See author guidelines, under 'Reporting Guidelines'. Please confirm you have submitted this list.	Not Applicable	

Data Availability

Data availability	Information included in the manuscript?	In which section is the information available? (Reagents and Tools Table, Materials and Methods, Figures, Data Availability Section)
Have **primary datasets** been deposited according to the journal's guidelines (see 'Data Deposition' section) and the respective accession numbers provided in the Data Availability Section?	Not Applicable	
Were **human clinical and genomic datasets** deposited in a public access-controlled repository in accordance to ethical obligations to the patients and to the applicable consent agreement?	Not Applicable	
Are **computational models** that are central and integral to a study available without restrictions in a machine-readable form? Were the relevant accession numbers or links provided?	Not Applicable	
If publicly available data were reused, provide the respective **data citations in the reference list**.	Not Applicable	
